# New Cyclopiane Diterpenes and Polyketide Derivatives from Marine Sediment-Derived Fungus *Penicillium antarcticum* KMM 4670 and Their Biological Activities

**DOI:** 10.3390/md21110584

**Published:** 2023-11-09

**Authors:** Anton N. Yurchenko, Olesya I. Zhuravleva, Olga O. Khmel, Galina K. Oleynikova, Alexandr S. Antonov, Natalya N. Kirichuk, Viktoria E. Chausova, Anatoly I. Kalinovsky, Dmitry V. Berdyshev, Natalya Y. Kim, Roman S. Popov, Ekaterina A. Chingizova, Artur R. Chingizov, Marina P. Isaeva, Ekaterina A. Yurchenko

**Affiliations:** 1 G.B. Elyakov Pacific Institute of Bioorganic Chemistry, Far Eastern Branch of the Russian Academy of Sciences, Prospect 100-Letiya Vladivostoka, 159, Russky Island, Vladivostok 690022, Russia; zhuravleva.oi@dvfu.ru (O.I.Z.); antonov_as@piboc.dvo.ru (A.S.A.); sheflera@bk.ru (N.N.K.); v.chausova@gmail.com (V.E.C.); kaaniv@piboc.dvo.ru (A.I.K.); berdyshev@piboc.dvo.ru (D.V.B.); kim_ny@piboc.dvo.ru (N.Y.K.); popov_rs@piboc.dvo.ru (R.S.P.); martyyas@mail.ru (E.A.C.); chingizov_ar@piboc.dvo.ru (A.R.C.); issaeva@piboc.dvo.ru (M.P.I.); 2 Institute of High Technologies and Advanced Materials, Far Eastern Federal University, 10 Ajax Bay, Russky Island, Vladivostok 690922, Russia; khmel.oo@dvfu.ru

**Keywords:** *Penicillium antarcticum*, phylogeny, re-identification, cyclopianes, polyketides, antimicrobial activity, sortase A, cytotoxicity

## Abstract

Two new cyclopiane diterpenes and a new cladosporin precursor, together with four known related compounds, were isolated from the marine sediment-derived fungus *Penicillium antarcticum* KMM 4670, which was re-identified based on phylogenetic inference from ITS, *BenA*, *CaM*, and *RPB2* gene regions. The absolute stereostructures of the isolated cyclopianes were determined using modified Mosher’s method and quantum chemical calculations of the ECD spectra. The isolation from the natural source of two biosynthetic precursors of cladosporin from a natural source has been reported for the first time. The antimicrobial activities of the isolated compounds against *Staphylococcus aureus*, *Escherichia coli*, and *Candida albicans* as well as the inhibition of staphylococcal sortase A activity were investigated. Moreover, the cytotoxicity of the compounds to mammalian cardiomyocytes H9c2 was studied. As a result, new cyclopiane diterpene 13-*epi*-conidiogenone F was found to be a sortase A inhibitor and a promising anti-staphylococcal agent.

## 1. Introduction

Marine fungi are a significant source of compounds with antimicrobial properties [1], among which sediment-derived fungi can be one of the promising sources of novel secondary metabolites [2].

The section *Canescentia* of the genus *Penicillium*, besides species *P. canescens* and *P. janczewskii*, includes such widespread species as *P. antarcticum*, *P. atrovenetum,* and *P. novae-zeelandiae* which are predominantly isolated from soil and leaf litter [3]. Difficulties in using morphological characters to identify strains make identification of this group continually problematic and induce a re-evaluation using phylogenetic data. Earlier *Penicillium attenuatum* KMM 4671, *P. ochotense* KMM 4670, and *P. piltunense* KMM 4668 were isolated from the Sea of Okhotsk and reported as new species [3], but recently Visagie and co-authors reduced these fungi to synonymy with *P. antarcticum* from series *Atroveneta* [4]. The assumption about several frameshift mutations in coding regions at sequence ends, which may imply low-quality sequence reads, needs to be verified.

Recently, seven fungal strains belonging to the *Atroveneta* series were investigated, and one or more species produced asperentins (2/7 species), atlantinones and rastins (4/7 species), patulin (3/7 species), atrovenetins (2/7 species), antarones (1/7 species), aurantiamine (1/7 species), benzomalvins (1/7 species), fischerin (1/7 species), and haenamindole (1/7 species) [4]. Various strains of *P. antarcticum* were described as producers of simple diketopiperazines *cis*-cyclo(4*R*-Hyp, *L*-Leu), *trans*-cyclo(4*R*-Hyp, *L*-Leu), *cis*-cyclo(4*R*-Hyp, *L*-Phe), cyclo-(*L*-Pro, Gly) [5], cladosporin (=asperentin) and its varied derivatives [6,7,8], antarones A and B [9], alkaloids aurantioclavine and chrysogine [6,7], itaconic acid and its derivatives [5], patulin, terrestric and violaceic acids [6,7], *cis*-4-hydroxymellein [5], and andrastin-type meroterpenoids [10]. Moreover, new carotane sesquiterpenoids piltunines A–F as well as known penigrisacid D were isolated from the fungus *Penicillium piltunense* KMM 4668 [11], which recently was suggested to be *P. antarcticum* [4]. Thus, *P. antarcticum* strains are rich sources of mainly structurally diverse polyketides, including well-known bioactive cladosporin.

Cladosporin was first isolated as an antifungal secondary metabolite from the endophytic fungus *Cladosporium cladosporioides* [12]. Later, its specific antimalarial activity-targeted *Plasmodium falciparum* cytosolic lysyl-tRNA synthetase was found [13]. Aminoacyl-tRNA synthetases (aaRSs) activate specific amino acids and attach them to tRNAs, which participate in the translation of messenger RNAs and synthesis of proteins at the ribosome. These enzymes are pivotal for protein biosynthesis and seminal targets for drug discovery and design. Currently, the IleRS inhibitor mupirocin and the LeuRS inhibitor tavaborole are cases of the successful clinical viability of aaRS inhibitors [14]. Despite the high activity and specificity against *Plasmodium falciparum* as well as its antibacterial, antifungal, and herbicidal effects [15,16], cladosporin has a high clearance rate in the body and poor oral bioavailability in an in vivo assay [17]; therefore, it has never been put on the market as a drug. The search for cladosporin derivatives with better pharmacological properties is advisable, and some results have already been obtained [18]. Moreover, a study of secondary metabolites from cladosporin-producing fungi may result in the isolation of new antibiotics.

In the current study, we re-identified the fungal strain KMM 4670, isolated its secondary metabolites, and elucidated the structures of the obtained compounds. Moreover, the antimicrobial activity of isolated compounds against *Staphylococcus aureus, Escherichia coli,* and *Candida albicans* as well as its influence on sortase A activity were evaluated.

## 2. Results

### 2.1. Molecular Re-Identification of the Fungal Strain

**Figure 1:**
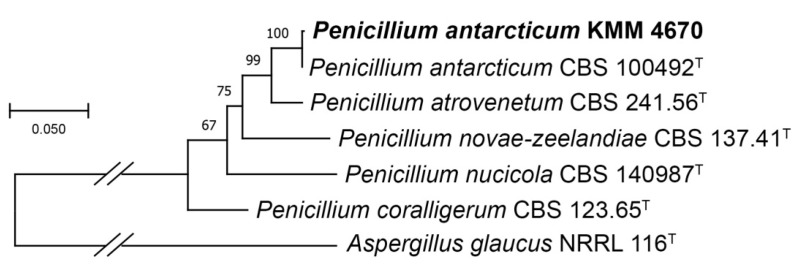
ML tree based on concatenated ITS–BenA–CaM–RPB2 nucleotide sequences showing the phylogenetic position of the strain KMM 4670 among members of genus *Penicillium* section *Canescentia*, series *Atroveneta*. Bootstrap values (%) of 1000 replications. The scale bars represent 0.05 substitutions per site.

In this study, to clarify the taxonomic position of the strain KMM 4670, we re-sequenced the molecular markers, such as ITS and partial *BenA* and *CaM* regions, and additionally sequenced the *RPB2* region. Approximately 580 bp fragments of the ITS region, about 500 bp fragments of the partial *BenA* and *CaM* regions, and 730 bp fragments of the partial *RPB2* gene were successfully amplified. A BLAST search showed that the partial *CaM* and *RPB2* gene sequences were 100% identical with the sequences of the ex-type strain *Penicillium antarcticum* CBS 100492, while the ITS and partial *BenA* regions were more than 99% identical. The phylogenetic ML tree of the concatenated ITS–*BenA*–*CaM–RPB2* gene sequences clearly showed that the strain KMM 4670 is clustered with the ex-type strain *P. antarcticum* CBS 100492 (Figure 1).

Thus, the new thorough investigation confirmed earlier conclusions that the strain KMM 4670 should be identified as *Penicillium antarcticum.*

### 2.2. Isolated Compounds

**Figure 2:**
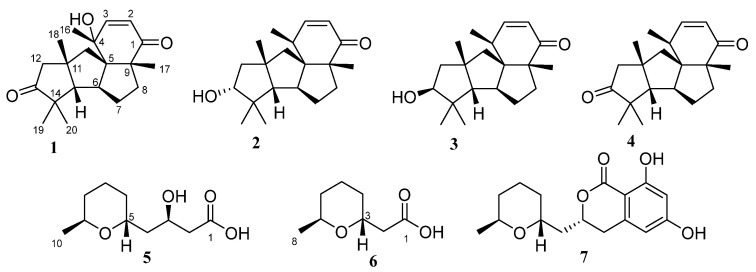
Structures of isolated compounds **1**–**7**.

From *P. antarcticum* KMM 4670, new cyclopiane diterpenes 4-hydroxyleptosphin C; (**1**), 13-*epi*-conidiogenone F (**2**), and new pentaketide derivative antaketide A (**5**) as well as known conidiogenone F (**3**) [19,20], leptosphin C; (**4**) [21], 2-((2*R*,6*S*)-6-methyltetrahydro-2H-pyran-2-yl)acetic acid (**6**) [22], and cladosporin (**7**) [12] were isolated (Figure 2).

The molecular formula of **1** was determined as C_20_H_28_O_3_ based on the analysis of the (+)-HRESIMS spectrum (Figure S38) containing the peak of the cationized molecule [M+Na]^+^ (*m*/*z* 339.1933) and was confirmed by the ^13^C NMR data. The ^1^H and ^13^C NMR spectra of compound **1** (Table 1, Figures S1 and S2) revealed the presence of five methyl groups (δ_C_ 34.3, δ_H_ 1.41; δ_C_ 30.1, δ_H_ 1.11; δ_C_ 29.1, δ_H_ 1.41; δ_C_ 21.7, δ_H_ 1.08; δ_C_ 20.6, δ_H_ 1.21), four *sp^3^*-methylene groups (δ_C_ 51.5, δ_H_ 2.85, 2.33; δ_C_ 48.8, δ_H_ 2.27, 1.87; δ_C_ 40.0, δ_H_ 2.12, 1.75; δ_C_ 34.5, δ_H_ 1.62, 1.23), two *sp^3^*-methine (δ_C_ 72.3, δ_H_ 1.86; δ_C_ 52.0, δ_H_ 2.79), and two *sp^2^*-methine (δ_C_ 157.0, δ_H_ 6.68; δ_C_ 126.1, δ_H_ 5.86) groups as well as four quaternary *sp^3^*-carbons (δ_C_ 65.1, 58.2, 50.7, 44.4), an oxygenated quaternary *sp^3^* carbon (δ_C_ 74.2), and two ketones (δ_C_ 224.9, 205.0).

**Figure 3:**
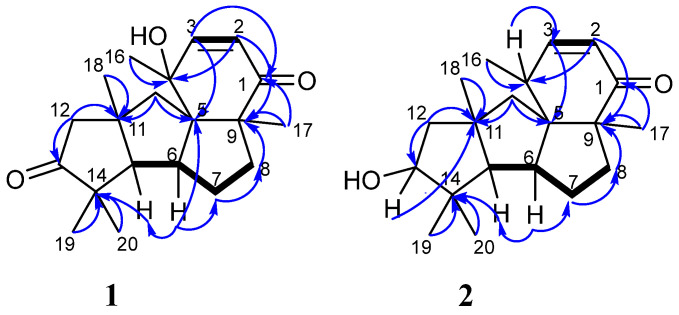
Key HMBC (arrows) and COSY (bold lines) correlations in compounds **1** and **2**.

The COSY correlations (Figure 3 and Figure S6) revealed the spin systems H(15)–H(6)–H(7)–H(8) and H(2)–H(3). The HMBC correlations (Figure 3 and Figure S5) from H-2 (δ_H_ 6.68) to C-4 (δ_C_ 74.2) and C-9 (δ_C_ 58.2); from H-3 (δ_H_ 5.86) to C-1 (δ_C_ 205.0), C-5 (δ_C_ 65.1), C-10 (δ_C_ 48.8), and C-16 (δ_C_ 29.1); from H_3_-16 (δ_H_ 1.41) to C-3 (δ_C_ 157.0), C-4, and C-5; from H_2_-8 (δ_H_ 2.12, 1.75) to C-1, C-7 (δ_C_ 34.5), and C-9; from H_2_-7 (δ_H_ 1.62, 1.23) to C-6 (δ_C_ 52.0), C-8 (δ_C_ 40.0), and C-15 (δ_C_ 72.3); from H-6 (δ_H_ 2.79) to C-4, C-5, C-7, C-14 (δ_C_ 50.7), and C-15; from H-15 (δ_H_ 1.86) to C-5, C-6, C-7, C-11 (δ_C_ 44.4), C-12 (δ_C_ 51.5), C-13 (δ_C_ 224.9), C-14 (δ_C_ 50.7), and C-20 (δ_C_ 30.1); from H_2_-10 (δ_H_ 2.27, 1.87) to C-4, C-5, C-11, and C-12; from H_2_-12 (δ_H_ 2.85, 2.33) to C-10, C-11, C-13, C-15, and C-18 (δ_C_ 34.3); from H_3_-17 (δ_H_ 1.21) to C-1, C-5, C-8, and C-9; from H_3_-18 (δ_H_ 1.41) to C-10, C-11, C-12, and C-15; and from both H_3_-19 (δ_H_ 1.08) and H_3_-20 (δ_H_ 1.11) to C-13, C-14, and C-15 established a cyclopiane-type core of **1** with keto-groups at C-1 and C-13 and a double bond between C-2 and C-3. A location of an OH-group at C-4 was suggested based on the downfield chemical shift of C-4 together with the presence of a third oxygen according to the MS data.

It should be noted that the structure and NMR data of **1** were very close to known leptosphin C (**4**) [21] with additional hydroxy group at C-4.

**Figure 4:**
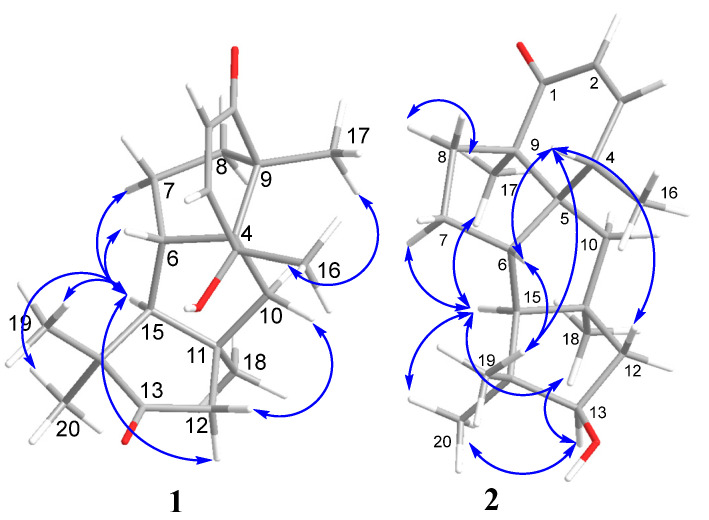
Key ROESY correlations in compounds **1** and **2**.

The relative stereoconfigurations of **1** were determined using ROESY data (Figure 4 and Figure S7). The ROESY correlations H-12α/H-10α; H-15/H-7β; H_3_-17/H-10β; H_3_-18/H-10β and H-15; H_3_-19/H-6; H_3_-20/H-12β; and H-15 revealed the same relative stereostructure with leptosphin C (**4**) [21] and other members of the cyclopiane group.

**Figure 5:**
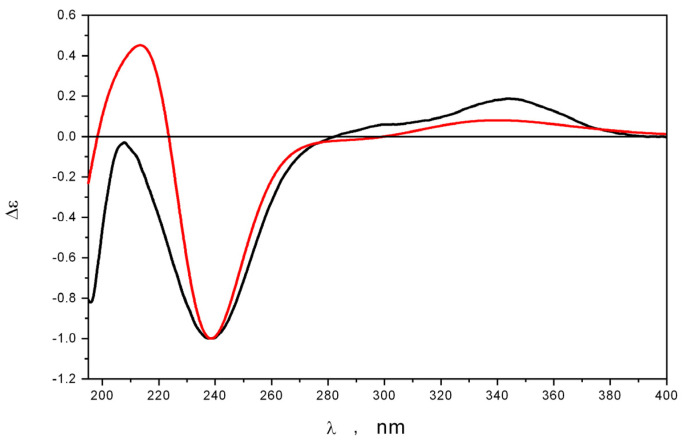
The experimental (black) and calculated (red) ECD spectra of **1**.

The absolute stereostructure of **1** was established as 4*R*,5*S,*6*S*,9*R*,11*S*,15*S* based on the comparison of experimental and calculated ECD spectra (Figure 5). Compound **1** was named 4-hydroxyleptosphin C.

The molecular formula of **2** was determined as C_20_H_30_O_2_ based on the (+)-HRESIMS spectrum (Figure S39) containing the peak of the cationized molecule [M+Na]^+^ (*m*/*z* 325.2142) and was confirmed by the ^13^C NMR data. The main features of ^1^H and ^13^C NMR spectra (Table 1, Figures S3 and S4) were close to those of known cyclopiane conidiogenone F (**3**) [19] with significant differences in some signals. The HMBC correlations (Figure 3 and Figure S12) confirmed a planar structure of **2** to be the same as conidiogenone F (**3**).

The ROESY correlations (Figure 4 and Figure S14) H-15 (δ_H_ 1.51)/H_3_-20 (δ_H_ 1.05), H_3_-18 (δ_H_ 1.22), H_3_-17 (δ_H_ 1.21), and H-7β (δ_H_ 1.19); H-13 (δ_H_ 3.92)/H_3_-20 and H_3_-18; H-4(δ_H_ 2.96)/H_3_-19 (δ_H_ 0.94), H-12α (δ_H_ 1.73), and H-6 (δ_H_ 2.52); H-6/H_3_-19; H_3_-17/H-8β (δ_H_ 2.08); H_3_-16 (δ_H_ 1.16)/H-10α (δ_H_ 2.03) and H-10β (δ_H_ 1.68); and H_3_-18/H-10β established the relative configurations of all stereocenters, which was the same as those for conidiogenone F (**3**) with the exception of C-13.

**Figure 6:**
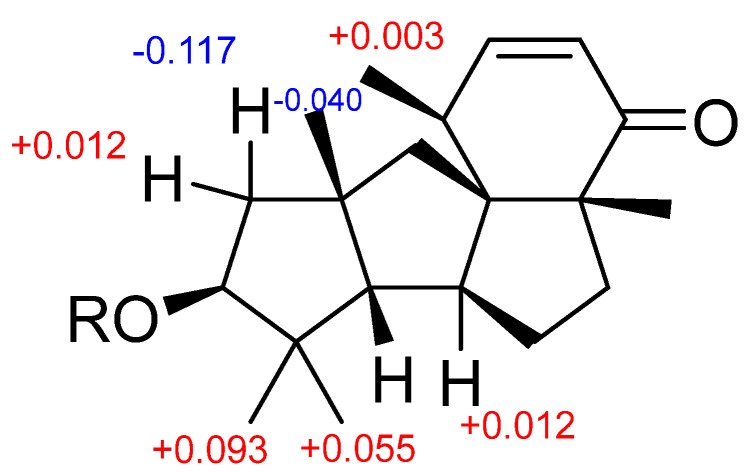
∆δ(δ_S_-δ_R_) values (in ppm) for MTPA esters of **3**.

The absolute configurations of the chiral centers of **2** could not be determined with a modified Mosher’s method [23] due to an insufficient amount of this compound. Therefore, this method was used for compound **3**. The esterification of **3** with (*S*)- and (*R*)-MTPA chloride occurred at the C-13 hydroxy group to yield the (*R*)-and (*S*)-MTPA esters **3a** and **3b**, respectively. The observed chemical shift differences Δδ(δ*_S_*-δ*_R_*) (Figure 6 and Figure S45–S48) indicated the 13*S* configuration, and therefore, the absolute configurations other chiral centers of **3** were established as 4*S*,5*S*,6*S*,9*R*,11*S*,15*S*. Due to the identity of relative stereochemistry in the diterpene core, the absolute configurations of **2** were determined as 4*S*,5*S*,6*S*,9*R*,11*S*,13*R*,15*S*. Compound **2** was named 13-*epi*-conidiogenone F.

The absolute configurations of leptosphin C; (**4**) were suggested as 4*S*,5*S,*6*S*,9*R*,11*S*,15*S* based on obvious biogenetic relationships with **1**–**3**.

The molecular formula of **5** was determined as C_10_H_18_O_4_ based on the (+)-HRESIMS spectrum (Figure S42) containing the peak of the cationized molecule [M+Na]^+^ (*m*/*z* 225.1097) and was confirmed by the ^13^C NMR data. The ^1^H and ^13^C NMR spectra of **5** (Table 2 and Figures S19 and S20) showed the presence of a methyl group (δ_C_ 18.6, δ_H_ 1.23), five methylene groups (δ_C_ 41.2, δ_H_ 2.57, 2.56; δ_C_ 39.4, δ_H_ 1.87, 1.58; δ_C_ 30.8, δ_H_ 1.72, 1.37; δ_C_ 30.7, δ_H_ 1.62, 1.42; δ_C_ 18.3, δ_H_ 1.72, 1.64), three oxygenated methine groups (δ_C_ 68.3, δ_H_ 4.05; δ_C_ 67.8, δ_H_ 4.08; δ_C_ 65.9, δ_H_ 4.29), and a carboxyl group (δ_C_ 174.9).

**Figure 7:**
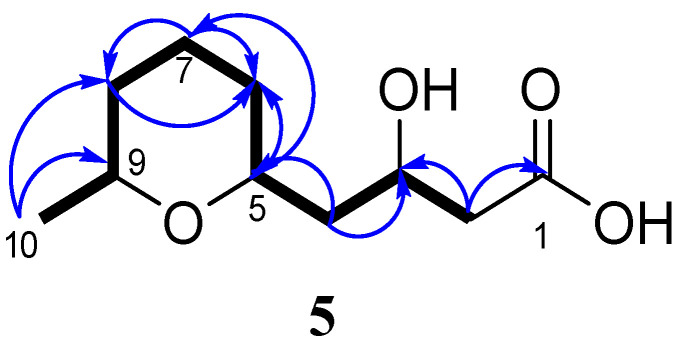
Key HMBC (arrows) and COSY (bold lines) correlations in compound **5**.

A single spin system H_2_(2)–H(3)–H_2_(4)–H(5)–H_2_(6)–H_2_(7)–H_2_(8)–H(9)–H_3_(10) was elucidated from the COSY correlations (Figure 7 and Figure S24). The HMBC correlations of **5** (Figure 7 and Figure S23) from H_2_-2 (δ_H_ 2.56, 2.57) to C-1 (δ_C_ 174.9) and C-3 (δ_C_ 65.9); from H-3 (δ_H_ 4.29) to C-1; from H-5 (δ_H_ 4.08) to C-4 (δ_C_ 39.4), C-6 (δ_C_ 30.6), and C-9 (δ_C_ 68.3); and from H-9 (δ_H_ 4.05) to C–5 (δ_C_ 67.8), C-8 (δ_C_ 30.8), and C-10 (δ_C_ 18.6) make it possible to close the tetrahydropyran ring through oxygen between C-5 and C-9 and add a carboxyl group to methylene C-2.

The NOESY correlations (Figure S25) between H-5 and H_3_-10 (δ_H_ 1.23) revealed the same relative stereostructure of the tetrahydropyrane moiety of **5**.

To establish absolute stereo configurations, an attempt was made to apply a modified Mosher’s method. Unfortunately, in the preparation of MTPA esters at 3-OH, compound **5** was destroyed. Nevertheless, the absolute configurations of all stereocenters in **5** were suggested based on obvious biogenetic relationships between **5**, **6,** and **7** [17].

To the best of our knowledge, the established structure of compound **5** was new, and this compound was named antaketide A.

### 2.3. Biological Activity of Isolated Compounds

#### 2.3.1. The Influence on *S. aureus*, *E. coli,* and *C. albicans*

The influence of compounds **1**–**6** on the growth and biofilm formation of *S. aureus*, *E. coli,* and *C. albicans* was investigated. Cladosporin (**7**) is a naturally occurring fungal metabolite with potent antibacterial, antifungal, insecticidal, and anti-inflammatory activities as well as plant growth regulatory effects [16]. Therefore, its biological activity in the present work was not studied.

**Figure 8:**
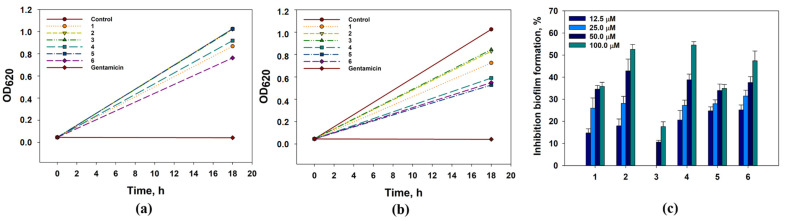
The effect of compounds **1**–**6** at concentrations of 12.5 µM (**a**) and 100.0 µM (**b**) on *Staphylococcus aureus* growth. Gentamicin was used as a positive control at a concentration of 1 mg/mL. (**c**) The effect of compounds **1**–**6** on the biofilm formation. The data are presented as a mean ± standard mean error. All experiments were carried out in independent triplicates.

The effects of compounds **1**–**6** on the growth and biofilm formation of Gram-positive bacteria *Staphylococcus aureus* are presented in Figure 8.

4-Hydroxyleptosphin C (**1**), at concentrations of 12.5 µM and 100 µM, inhibited *S. aureus* growth by 15.3% and 29.3%, respectively. The prevention of the biofilm formation by 15.9% and 34.5% was observed when the concentrations of **1** were 12.5 µM and 100 µM, respectively.

13-*epi*-conidiogenone F (**2**) showed a weak inhibition of *S. aureus* growth by 19.1% at 100 µM and no effect at 12.5 µM, but this one significantly prevented *S. aureus* biofilm formation from 37.9% at 12.5 µM to 52.6% at 100 µM. The half-maximal concentration (IC_50_) of the biofilm formation inhibition was calculated as 76.1 µM.

Conidiogenone F (**3**) inhibited *S. aureus* growth by 17.6% when it was used at 100 µM. At 12.5 µM, conidiogenone F (**3**) did not influence *S. aureus* growth. Moreover, conidiogenone F (**3**) prevented *S. aureus* biofilm formation by 10–15% only at concentrations of 50 µM and 100 µM. Leptosphin C; (**4**) inhibited *S. aureus* growth by 42.4% and 10.3% at 100 µM and 12.5 µM, respectively. Moreover, leptosphin C; (**4**) prevented the biofilm formation by 54.5% at 100 µM. The IC_50_ for biofilm formation inhibition was calculated as 85.5 µM.

Antaketide A (**5**) and tetraketide derivative **6** showed similar inhibition effects on *S. aureus* growth and biofilm formation. Antaketide A (**5**) inhibited *S. aureus* growth by 48.5% at 100 µM, and this one did not show any influence on *S. aureus* growth at 12.5 µM. Compound **6** inhibited *S. aureus* growth by 46.5% and 25.6% at 100 µM and 12.5 µM, respectively. In addition, **5** and **6** prevented the biofilm formation of *S. aureus* by nearly 30–40%.

**Figure 9:**
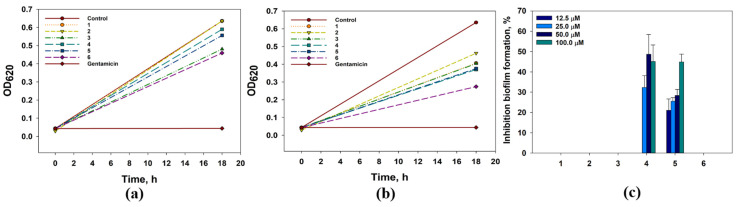
The effect of compounds **1**–**6** at concentrations of 12.5 µM (**a**) and 100.0 µM (**b**) on *E. coli* growth. Gentamicin was used as a positive control at a concentration of 1 mg/mL. (**c**) The effect of compounds **1**–**6** on the biofilm formation. The data are presented as a mean ± standard mean error. All experiments were carried out in independent triplicates.

The effects of compounds **1**–**6** on the growth and biofilm formation of Gram-negative bacteria *Escherichia coli* are presented in Figure 9.

All investigated compounds inhibited the growth of *E. coli* culture but only compounds **4** and **5** affect the biofilm formation of *E. coli*. 4-Hydroxyleptosphin C (**1**) and 13-*epi*-conidiogenone F (**2**) at a concentration of 100 µM inhibited *E. coli* growth by 35.9% and 27.3%, respectively. The growth inhibitory effect of these two compounds was not observed when its concentration was 12.5 µM.

Conidiogenone F (**3**) and leptosphin C; (**4**) at a concentration of 100 µM inhibited *E. coli* growth by 36.4% and 41.7%, respectively. At 12.5 µM, conidiogenone F (**3**) inhibited *E. coli* growth by 24.3% while leptosphin C; (**4**) inhibited it by only 7.2%. Nevertheless, **4,** at concentrations of 25–100 µM, prevented *E. coli* biofilm formation by nearly 30–40%.

Antaketide A (**5**) at 100 µM and 12.5 µM inhibited *E. coli* growth by 40.9% and 12.5%, respectively. Moreover, **5** prevented the biofilm formation by 21–45% at concentrations of 12.5–100 µM, respectively. Finally, tetraketide derivative **6** at 100 µM and 12.5 µM inhibited *E. coli* growth by 56.9% and 27.7%, respectively, and IC_50_ was calculated as 84.9 µM.

**Figure 10:**
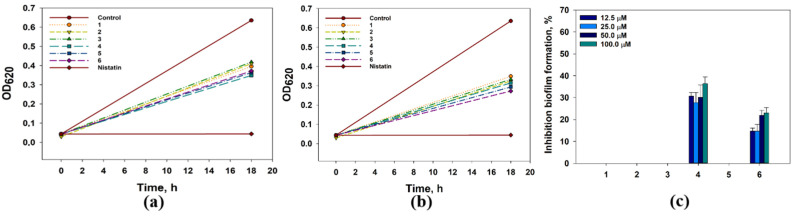
The effect of compounds **1**–**6** at concentrations of 12.5 µM (**a**) and 100.0 µM (**b**) on *C. albicans* growth. The effect of compounds **1**–**6** on the biofilm formation. Nitrofungin was used as a positive control at a concentration of 1 mg/mL. The data are presented as a mean ± standard mean error. All experiments were carried out in independent triplicates.

The effects of compounds **1**–**6** on the growth and biofilm formation of *Candida albicans* are presented in Figure 10.

All compounds inhibited *C. albicans* growth by nearly 30–40%, and compounds **4** and **6** also affected the biofilm formation by *C. albicans*. Compounds **1**–**3** at a 12.5 µM inhibited *C. albicans* growth by 30.4%, 27.9%, and 26.5%, respectively. Compound **4** inhibited *C. albicans* growth by 38.7–44.8% and the biofilm formation by 30.6–36.4% at concentrations from 12.5 to 100 µM. Compound **5** inhibited *C. albicans* growth by 36.2–48.4% and did not affect the biofilm formation. Compound **6** inhibited *C. albicans* growth by 34.7-52.1% and the biofilm formation by 14.7–23.1% at concentrations from 12.5 to 100 µM. Its IC_50_ for *C. albicans growth* was calculated as 89.9 µM.

#### 2.3.2. The Influence on Sortase A Activity

Sortase A enzyme is key for biofilm formation and virulence of *S. aureus* and sortase A’ inhibitors can prevent the biofilm formation [24]. Therefore, a natural inhibitor of sortase A activity rhodionin decreases the adhesion of *S. aureus* to fibrinogen via reducing the capacity of protein A on the bacterial surface and biofilm formation without affecting the survival and growth of bacteria [25]. 

**Figure 11:**
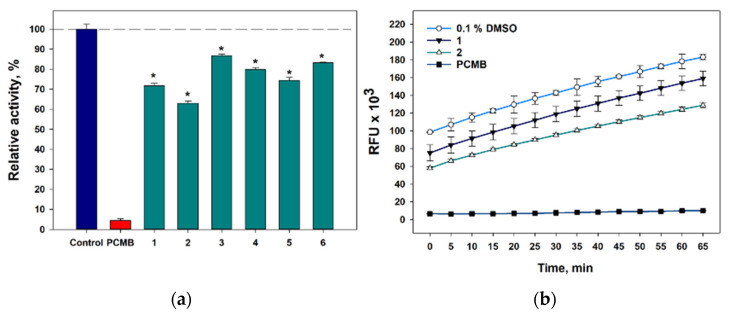
The effects of compounds **1**–**6** on sortase A activity after 10 min of incubation (**a**) and time-dependent graph of the inhibitory effect of 4-hydroxyleptosphin C (**1**) and 13-*epi*-conidiogenone F (**2**). (**b**) 4-(Hydroxymercuri)benzoic acid (PCMB) was used as a control. All experiments were carried out in triplicate. The data are presented as a mean ± standard error of the mean (SEM). * indicates significant differences between the control (DMSO 0.8%) and compounds (*p* value ≤ 0.05).

The investigated compounds **1**–**6** significantly reduced the formation of *S. aureus* biofilm; therefore, the influence of compounds **1**–**6** on the activity of sortase A studied in a cell-free test using SensoLyte 520 Sortase A Activity Assay Kit Fluorometric and data are presented in Figure 11.

All compounds at 50 µM statistically significantly inhibited the activity of sortase A (Figure 11a). 4-Hydroxyleptosphin C (**1**) and 13-*epi*-conidiogenone F (**2**) inhibited sortase A activity by 28.2% and 36.9%, and this effect continued during all experiments (Figure 11b). Compounds **3**-**6** inhibited the sortase A activity by 13.1%, 20.0%, 25.5%, and 16.8%, respectively.

13-*epi*-Conidiogenone F (**2**) is a stereo isomer of conidiogenone F (**3**) at C-13, and the β-position of OH-group at C-13 in the structure of **2** is possibly better for interaction with sortase A that caused higher inhibition of sortase A activity. To confirm or refute this assumption as well as determine the contribution of an additional OH-group to the activity of **1**, we carried out the molecular docking of compounds **1**-**4** with sortase A structure (PDB ID 1T2P) using SwissDock [26]. 

In the apo structure of sortase A (PDB ID 1T2P), a V-shaped pocket is formed by the β4, β7, and β8 strands on one side of the β barrel, together with three surrounding loops. The left side of the pocket is a hydrophobic tunnel formed by Ala92, Ala104, Ala118, Val161, Pro163, Val166, Val 168, Ile182, Val193, Trp194, Ile199, and Val201, along with two putative catalytic residues: Cys184 and Arg197. The right side of the pocket consists of several polar residues: Glu105, Asn114, Ser116, and Thr180 [27].

In our calculations, the most active sortase A inhibitor, 13-*epi*-conidiogenone F (**2**), can form a pose (∆G − −7.16274 kcal/mol) with the hydrogen-bonding interaction between Glu105 and its OH-group at C-13, and hydrophobic interaction between the keto-group at C1 and Gly192 as well as another hydrophobic interaction with Ala92, Thr93, Thr187, Trp194, and Ala104 (Table 3).

Conidiogenone F (**3**) can form a pose (∆G − −6.2875967 kcal/mol) with the hydrogen-bonding interaction between Arg197 and its OH-group at C-13 and hydrophobic interactions with only Ala104 and Ile182. 

4-Hydroxyleptosphin C (**1**) can form a pose (∆G − −6.722709 kcal/mol) with the hydrogen-bonding interactions between its keto-group at C-4 and Arg197 and OH-group at C-4 and Glu105 and hydrophobic interactions with Ile199, Ile182. Another stable pose (∆G − −7.4239464 kcal/mol) formed with the hydrogen-bonding interaction between Arg197 and the keto-group at C-4 and hydrophobic interactions with Ala104, Ile182, Ala92, and Thr93.

Finally, leptosphin C; (**4**) can form a pose (∆G − −6.429369 kcal/mol) with the hydrogen-bonding interaction between Arg197 and its keto-group at C-13 and hydrophobic interactions with Ile182. Another stable pose (∆G − −7.032929 kcal/mol) has no hydrogen-bonding interactions and hydrophobic interactions with Ala92, Gly192, Ile182, and Ala104.

**Figure 12:**
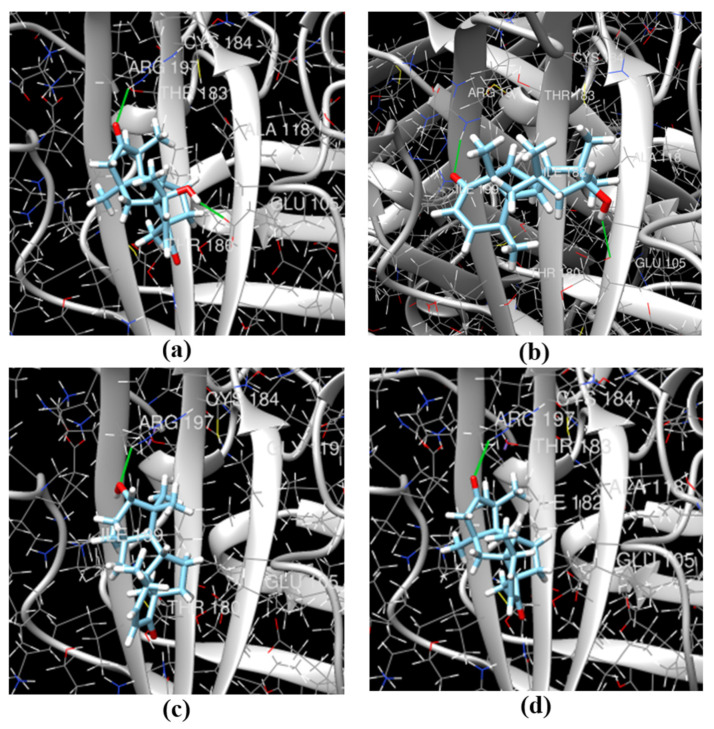
The molecular docking poses of cyclopiane diterpenes **1**–**4** with sortase A (PDB ID 1T2P): **1** (**a**), **2** (**b**), **3** (**c**), **4** (**d**).

Thus, the *α*-OH-group at C-13 in the structure of **2** allows 13-*epi*-conidiogenone F (**2**) to form the interactions with the amino acid residue of sortase A from both sides of diterpene moiety (Figure 12b). At the time, the *β*-OH-group at C-13 in the structure of **3** allows for the interaction on only one side of the molecule (Figure 12c). The additional hydroxy group in the structure of **1** also allows this compound to contract with **4** forms the interactions with the amino acid residue of sortase A from both sides of the diterpene moiety (Figure 12a,d). 

#### 2.3.3. The Cardiotoxicity of Compounds **1**–**6**

Therefore, secondary metabolites from marine fungus *P. antarcticum* KMM 4670 as inhibitors of *S. aureus* sortase A and the biofilm formation can be promising for future investigations, but its toxicity toward mammalian cells should be tested before such conclusions. Cardiovascular toxicity remains a major cause of concern during preclinical and clinical development, as well as contributing to a post-approval withdrawal of medicines [28]. Therefore, macrolides erythromycin, azithromycin, and clarithromycin cause QT prolongation, torsades de pointes, and arrhythmia-affected cardiomyocyte mitochondria in isolated rat heart mitochondria [29]. The cardiotoxicity of fluoroquinolones was discussed in the review [30]. Thus, the investigation of the cardiotoxicity of leading molecules and drug candidates is actually in the pre-clinical stage. At this time, only three cultivating cardiomyocyte cultures, murine HL-1, rat H9c2, and human AC-16 cell lines, are available. Despite the limitations, the H9c2 cell line is the most used in various experiments [31], including the study of antibiotics’ cardiotoxicity [32].

The effect of compounds **1**–**6** on the viability of H9c2 cardiomyocytes is presented in Table 4.

Therefore, polyketides **5** and **6** showed significant toxicity toward normal cardiomyocytes at the concentrations in which they affected the growth of microorganisms indicates their non-specific action against living cells. 

Both compounds **1** and **2** caused the H9c2 viability to decrease by nearly 30% at a concentration of 100 µM. Moreover, compound **1** caused the decrease in H9c2 cell viability, near 30%, at a concentration of 10 µM, while compound **2**–**4** was non-toxic.

## 3. Discussion

### 3.1. Cyclopiane Derivatives from P. antarcticum KMM 4670

Cyclopiane-type diterpenes were discovered 20 years ago, and since then, about 25 related compounds have been described. These compounds are usually produced by a number of fungi of the subgenus *Penicillium*, except one report of an isolation from a fungus of the genus *Leptosphaeria*. Structurally, cyclopianes are characterized by a conserved 5/5/5/6 tetracyclic core with methyl groups at C-4, C-9, C-11, and C-14 as well as an oxygenated C-1 atom. The biogenesis of cyclopiane diterpenes was proposed from geranyl geraniol pyrophosphate via hypothetical deoxyconidiogenol [21]. Unfortunately, the absolute stereoconfigurations of these compounds were not established in most of the published works, and in a number of other works, the absolute stereochemistry is questionable. This has led to high confusion about stereostructures of cyclopianes. This study is the very first proof of absolute stereoconfigurations of known conidiogenone F (**3**) and leptosphin C; (**4**) using a modified Mosher’s method. Despite the identity of the ECD data of compound **3** with conidiogenone F, the absolute stereostructure of **3** established by us is a mirror structure of that proposed earlier for conidiogenone F [19]. It should be noted that, in recent papers [33,34], conidiogenone F was depicted identically to that established by us. However, no evidence for such an absolute stereostructure has been previously published. The absolute stereostructure of leptosphin C (**4**), determined by us from the obvious biogenetic relationship with conidiogenone F (**3**), is also enantiomeric in comparison with the previously published [21]. 

Earlier it was reported that conidiogenone F (**3**) at 50 μg/mL inhibited bacteria *Escherichia coli* growth and did not inhibit the growth of *Staphylococcus aureus* when this one was used at concentrations up to 100 μg/mL [21]. Our own experiments confirm that conidiogenone F (**3**) is more active against Gram-negative *E. coli* growth. 

In the same work, it was reported that leptosphin C (**4**) showed antibacterial activity against *Staphylococcus aureus* (100 μg/mL) and *Escherichia coli* (>100 μg/mL) as well as another bacterial strain [21]. In this work, authors used bacterial suspension at a concentration of 10^6^ CFU per milliliter and did not use any instrumental equipment for the detection of bacterial growth inhibition. This probably explains why the authors recorded the activity of **3** and **4** at concentrations 166 µM and 333 µM (calculated from 50 and 100 μg/mL) only, whereas in our experiments the activity was detected at significantly lower concentrations. 

### 3.2. Polyketides from P. antarcticum KMM 4670

As was shown recently, tetraketide **5** and pentaketide **6** are intermediates in the biosynthesis of cladosporin (**7**). Thus, **6** is formed from a linear tetraketide precursor by its cyclization, and **5** is the product of the elongation of **6**, with one unit of malonyl-CoA. Both compounds are biosynthesized by the highly reducing I type polyketide synthase. Further formation of cladosporin is controlled by another non-reducing I type polyketide synthase [35]. It proves the absolute stereostructures of both compounds to be the same with cladosporin (**7**). It should be noted that **6** was isolated earlier as a product of the synthetic degradation of cladosporin [36], and its epimer was reported as a civet constituent [22]. Synthetic N-acetylcysteamine thioester derivative of **5** was used for the in vitro biosynthesis of cladosporin by non-reducing I type polyketide synthase [35]. Thus, the current research is the very first report of isolation of cladosporin precursors **5** and **6** as individual natural compounds. 

Earlier the antimicrobial activity for **6** was not reported. In our experiments, this one and antaketide A (**5**) showed the highest inhibition activity against the growth of all used test-strains. However, they significantly inhibited biofilm formation in the *S. aureus* culture only, and this effect was supported by the inhibition of sortase A activity. However, we observed the cytotoxicity of the cladosporin precursors **5** and **6** to H9c2 cardiomyocytes, which makes these compounds not as promising leading molecules as we would like. 

### 3.3. Biological Activity of Cyclopiane Diterpenes

Cyclopiane diterpenes **1**–**4** have a little difference in their chemical structures, but these differences result in differences in their bioactivity. According to their cytotoxic activity, substances can be arranged in the following order: **1** > **2** > **3** = **4**. According to their ability to inhibit the microbial growth, it will be the order: **4** > **1** > **2** = **3** (*S. aureus*), **3** > **4** > **1** = **2** (*E. coli*), and **1** = **2** = **3** = **4** (*C. albicans*), but the differences are not so big. According to their ability to inhibit biofilm formation, it will be the order: **2** = **4** > **1** > **3** (*S. aureus*), **4** >> **1** = **2** = **3** (*E. coli*), and **4** >> **1** = **2** = **3** (*C. albicans*). Finally, the order of compounds according to their ability to inhibit the sortase A activity is **2** > **1** > **4** > **3**. 

Obviously, that the presence of an additional OH-group at C-4 in the structure of the compound new cyclopiane diterpene 4-hydroxyleptosphin C (**1**), in contrast with leptosphin C (**4**), resulted in its higher cytotoxic and sortase A inhibition activities.

It is believed that antibiotics aimed at the vital functions of bacteria cause the development of resistance much faster. The appearance of penicillin-resistant *S. aureus* strains was registered in 1949, while penicillin was put into practice in the early 40s. The appearance of methicillin and vancomycin-resistant strains was also not long in coming [37]. The identification and analysis of the virulence factors used by pathogens to colonize, invade, and persist within a susceptible host resulted in a new strategy, suggesting that drugs blocking these factors without killing the bacteria create less evolutionary pressure and diminish the chances of resistant genes to emerge. This strategy offers the targeting of the inhibition of the quench pathogen quorum sensing systems or the inhibition of the biofilm formation in the discovery of new antibiotics [38]. 

Bacteria can survive in two forms: planktonic cells and biofilm, and the formation of biofilm depends on many factors, including the attachment of cell wall components to the cell wall [39]. Staphylococcal sortase A (EC 3.4.22.70) is the cysteine transpeptidase and mainly acts as an anchor surface protein [40]. About 25 years have passed since the beginning of the study of sortase A inhibitors [41], and during this time, a large amount of experimental data has accumulated [42]. Experiments involving laboratory animals confirm that sortase A inhibitors are effective not only in in vitro, but also in in vivo studies [43]. For example, flavonol glycoside hibifolin inhibited sortase A activity, decreased the adhesion of *S. aureus* bacteria to the host cells and the biofilm formation, and, in combination with cefotaxime, protected mice from *S. aureus* infection-induced pneumonia [44]. 

The natural compounds of various classes, such as alkaloids, polyketides, quinones, and others, were described as sortase A inhibitors. In our experiments, first, it was shown that cyclopiane diterpenes can affect sortase A activity and biofilm formation. The compounds 13-*epi*-conidiogenone F (**2**) and 4-hydroxyleptosphin C (**1**) showed a more effective inhibition of sortase A activity, but 4-hydroxyleptosphin C (**1**) had more cardiotoxicity. Leptosphin C; (**4**) inhibited sortase A activity less than 13-*epi*-conidiogenone F (**2**) but can also be interesting for future investigations due to the low cardiotoxicity. 

## 4. Materials and Methods

### 4.1. General Experimental Procedures

Optical rotations were measured on a Perkin–Elmer 343 polarimeter (Perkin Elmer, Waltham, MA, USA). UV spectra were recorded on a Shimadzu UV-1601PC spectrometer (Shimadzu Corporation, Kyoto, Japan) in methanol. CD spectra were measured with a Chirascan-Plus CD spectrometer (Leatherhead, UK) in methanol. NMR spectra were recorded in CDCl_3_, acetone-*d_6_*, and DMSO-*d_6_* on a Bruker DPX-300 (Bruker BioSpin GmbH, Rheinstetten, Germany), a Bruker Avance III-500 (Bruker BioSpin GmbH, Rheinstetten, Germany), and a Bruker Avance III-700 (Bruker BioSpin GmbH, Rheinstetten, Germany) spectrometer, using TMS as an internal standard. HRESIMS spectra were measured on a Maxis impact mass spectrometer (Bruker Daltonics GmbH, Rheinstetten, Germany). Microscopic examination and photography of fungal cultures were performed with Olympus CX41 microscope equipped with an Olympus SC30 digital camera. Detailed examination of ornamentation of the fungal conidia was performed using scanning electron microscopy (SEM) EVO 40.

Low-pressure liquid column chromatography was performed using silica gel (60/100 μm, Imid Ltd., Krasnodar, Russia) and Gel ODS-A (12 nm, S—75 um, YMC Co., Ishikawa, Japan). Plates precoated with silica gel (5–17 μm, 4.5 cm × 6.0 cm, Imid Ltd., Russia) and silica gel 60 RP-18 F_254_S (20 cm × 20 cm, Merck KGaA, Darmstadt, Germany) were used for thin-layer chromatography. Preparative HPLC was carried out on an Agilent 1100 chromatograph (Agilent Technologies, Santa Clara, CA, USA) with an Agilent 1100 refractometer (Agilent Technologies, Santa Clara, CA, USA) and a Shimadzu LC-20 chromatograph (Shimadzu USA Manufacturing, Canby, OR, USA) with a Shimadzu RID-20A refractometer (Shimadzu Corporation, Kyoto, Japan) using YMC ODS-AM (YMC Co., Ishikawa, Japan) (5 µm, 10 × 250 mm), YMC ODS-AM (YMC Co., Ishikawa, Japan) (5 µm, 4.6 × 250 mm), a HyperClone ODS (Phenomenex, Torrance, CA, USA) (5 µm, 4.6 × 250 mm), and Hydro-RP (Phenomenex, Torrance, CA, USA) (4 μm, 10 × 250 mm) columns.

### 4.2. Fungal Strain

The fungal strain KMM 4670 was isolated from submarine soil sample collected in the Sea of Okhotsk (the northeastern shelf of the Sakhalin Island). Earlier, the strain KMM 4670 was described by Kirichuk N.N. et al. [3] as a new species “*Penicillium ochotense*” closely related to *Penicillium antarcticum* (section *Canescentia*, series *Atroveneta*) based on DNA sequences of the ITS (internal transcribed spacer), *BenA* (β-tubulin), and *CaM* (calmodulin) regions. During this study, the strain was re-sequenced and re-identified based on sequences of ITS, partial *BenA*, *CaM,* and *RPB2* (RNA polymerase second large subunit) regions. The fungal strain is stored in the Collection of Marine Microorganisms (PIBOC FEB RAS, Vladivostok, Russia) under the code KMM 4670.

### 4.3. DNA Extraction and Amplification

Genomic DNA was isolated from fungal mycelium grown on MEA (malt extract agar) at 25 °C for 7 days using the MagJET Plant Genomic DNA Kit (Thermo Fisher Scientific, Waltham, MA, USA) according to the manufacturer’s protocol. PCR was conducted using GoTaq Flexi DNA Polymerase (Promega, Madison, WI, USA). For amplification of the *ITS* gene, the standard primer pair, ITS1 and ITS4 [45], was used. The reaction profile was 95 °C for 300 s; 35 cycles of 95 °C for 20 s, 55 °C for 30 s, 72 °C for 90 s; and, finally, 72 °C for 300 s. For amplification of the partial *BenA* gene, the primer pair, tub_P/A_F (5′-GGTAACCAAATYGGTGCTGCTTTC-3′) and Bt-2b [46], was used. The reaction profile was 95 °C for 150 s; 35 cycles of 95 °C for 20 s, 60 °C for 30 s, and 72 °C for 90 s; and, finally, 72 °C for 300 s. For amplification of the partial *CaM* gene, the degenerate primer pair, cal_P/A_F (5′-TCYGAGTACAAGGAGGCSTT-3′) and cal_P/A_R (5′-CCRATGGAGGTCATRACGTG-3′), was used. The reaction profile was 95 °C for 300 s; 35 cycles of 95 °C for 20 s, 65 °C for 30 s, and 72 °C for 90 s; and, finally, 72 °C for 300 s. For amplification of the partial *RPB2* gene, the degenerate primer pair, rpb2_Pen_F (5′-GAGACYAAYCGBGARATYTA-3′) and rpb2_Pen_R (5′-GTCATSACAATCATRATDGT-3′), was used. The reaction profile was an initial denaturation at 95 °C for 300 s, followed by 5 cycles of 95 °C for 20 s, 48 °C for 30 s, and 72 °C for 90 s; 5 cycles of 95 °C for 20 s, 50 °C for 30 s, and 72 °C for 90 s; 25 cycles of 95 °C for 20 s, 52 °C for 30 s, and 72 °C for 90 s; and, finally, 72 °C for 420 s. The amplified *ITS*, *BenA, CaM,* and *RPB2* genes were purified with the ExoSAP-IT™ PCR Product Cleanup Reagent (Thermo Fisher Scientific, Waltham, MA, USA). Sequencing was bidirectionally performed with the same primers on an Applied Biosystems SeqStudio Genetic Analyzer (Thermo Fisher Scientific, Waltham, MA, USA) using the Big Dye Terminator reagent kit, version 3.1. Gene sequences were deposited in GenBank under accession numbers KU358553.2 for *ITS*, KU358556.2 for the partial *BenA*, KU358559.2 for the partial *CaM,* and OR271597 for the partial *RPB2* (Table 1).

### 4.4. Phylogenetic Analysis

The ITS region, the partial *BenA*, *CaM,* and *RPB2* gene sequences of the fungal strain KMM 4670 and other members from the section *Canescentia*, series *Atroveneta,* were aligned using MEGA X software version 11.0.9 [47] using Clustal W algorithm. The ex-type homologs were searched in the GenBank database (http://ncbi.nlm.nih.gov, accessed on 8 November 2023) using the BLASTN algorithm (http://www.ncbi.nlm.nih.gov/BLAST, accessed on 20 July 2023). The phylogenetic analysis was conducted using MEGA X software [47]. The ITS region and partial *BenA*, *CaM,* and *RPB2* gene sequences were concatenated into one alignment. Phylogenetic tree was constructed according to the Maximum Likelihood (ML) algorithm based on the Tamura–Nei model [48]. The tree topology was evaluated with 1000 bootstrap replicates. The *Aspergillus glaucus* NRRL 116 ^T^ strain was used in the phylogenetic analysis as outgroup (Table 5).

### 4.5. Cultivation of Fungus for Metabolite Isolation

The fungal strain KMM 4670 was cultured on a rice medium at 22 °C for three weeks in 100 Erlenmeyer flasks (500 mL), each containing 20 g of rice, 20 mg of yeast extract, 10 mg of KH_2_PO_4_, and 40 mL of natural seawater from the Marine Experimental Station of PIBOC, Troitsa (Trinity) Bay, the Sea of Japan.

### 4.6. Extraction and Isolation

At the end of cultivation, the mycelium of the fungal strain KMM 4670, together with the medium, was homogenized and extracted with EtOAc (1 L). The obtained extract was concentrated in vacuo. The dry residue (57.5 g) was dissolved in H_2_O−EtOH (4:1) (300 mL) and extracted successively with *n*-hexane (3 × 0.2 L), EtOAc (3 × 0.2 L), and butanol-1 (3 × 0.2 L). The ethyl acetate extract was evaporated to dryness (2.6 g) and chromatographed on a silica gel column (3 × 14 cm), which was first eluted with *n*-hexane–EtOAc from 100% *n*-hexane with a stepwise gradient of 10% to 100% EtOAc (total volume 20 L). Fractions of 250 mL were collected and combined based on TLC data.

The fraction eluted with EtOAc–*n*-hexane (20:80, 765 mg) was separated on a YMC ODS-A column (1.5 × 5.5 cm), which was eluted with a step gradient from 40% to 100% MeOH in H_2_O (total volume 1 L). Subfraction I, eluted with MeOH–H_2_O (40:60, 46 mg), was purified with HPLC on a YMC ODS-AM column (5 µm, 10 × 250 mm), eluted MeOH-H_2_O (40:60), and on Phenomenex Hydro-RP column (4 µm, 10 × 250 mm) to give compounds **5** (10.1 min, 4.6 mg) and **6** (9.0 min, 1.2 mg). Subfraction II, eluted with MeOH–H_2_O (60:40, 62 mg), was separated on a YMC ODS-AM column, and then, it was re-chromatographed with HPLC on a HyperClone ODS column (5 µm, 4.6 × 250 mm), eluted with MeCN–H_2_O (55:45), to give compounds **1** (12.0 min, 0.7 mg), **2** (8.8 min, 0.6 mg), **3** (10.0 min, 2.8 mg), and **4** (14.9 min, 6.7 mg).

### 4.7. Spectral Data

4-Hydroxyleptosphin C (**1**): [α]_D_^20^—71.43 (c 0.007, MeOH); UV (MeOH) λ_max_ (log ε) 219 (3.78), 198 (3.79), 210 (3.77) (Figure S23 from Supplementary Materials CD (c 2.21 mM, MeOH) λ_max_ (∆ε) 196 (−5.13), 208 (−0.15), 238 (−6.17), 300 (0.36), 345 (1.14) nm, (Figure S24 from Supplementary Materials ^1^H and ^13^C NMR data are provided in Table 1 (Figures S1 and S2 from Supplementary Materials); HRESIMS *m*/*z* 339.1933 [M+Na]^+^ (calcd for C_20_H_28_O_3_Na 339.1931).

13-*epi*-Conidiogenone F (**2**): [α]_D_^20^—38.30 (c 0.006, MeOH); UV (MeOH) λ_max_ (log ε) 195 (3.86) (Figure S25 from Supplementary Materials CD (c 0.33 mM, MeOH) λ_max_ (∆ε) 207 (−1.25), 220 (−1.30), 255 (0.10), 288 (0.03), 3296 (0.06) nm (Figure S26 from Supplementary Materials); ^1^H and ^13^C NMR data are provided in Table 1 (Figures S8 and S9 from Supplementary Materials); HRESIMS *m*/*z* 325.2142 [M+Na]^+^ (calcd for C_20_H_30_O_2_Na 325.2138).

Antaketide A (**5**): [α]_D_^20^—108.3 (c 0.012, MeOH), UV (MeOH) λ_max_ (log ε) (Figure S31 from Supplementary Materials); CD (c 0.99 mM, MeOH) λ_max_ (∆ε) nm, (Figure S32 from Supplementary Materials ^1^H and ^13^C NMR data are provided in Table 2 (Figures S1–S20 from Supplementary Materials); HRESIMS *m*/*z* 225.1097 [M+Na]^+^ (calcd for C_10_H_18_O_4_Na 225.1097).

### 4.8. Preparation of (S)-MTPA and (R)-MTPA Esters of Conidiogenone F (**3**)

A few crystals of 4-dimethylaminopyridine and (*R*)-MTPA-Cl (5 μL) were added to a solution of **3** (0.6 mg) in anhydrous pyridine at room temperature and stirred for 4 h. After evaporation of the solvent, the residue was purified with HPLC on a YMC Chiral column (MeCN–H_2_O, 90:10) to afford the (*S*)-MTPA ester (10.8 min, 0.65 mg). The (*R*)-MTPA ester (11.6 min, 0.16 mg) was prepared in a similar manner using (*S*)-MTPA-Cl.

(*S*)-MTPA ester of **3** (**3a**): ^1^H NMR (CDCl_3_, 500.13 MHz) δ: 6.985 (1H, q, *J =* 10.1, 5.9Hz, H-3), 5.973 (1H, dd, *J =* 10.1, 0.9 Hz, H-2), 5.043 (1H, t, *J =* 8.7 Hz, H-13), 3.524 (3H, d, *J =* 0.9 Hz, OMe), 2.692 (1H, m, H-4), 2.304-2.249 (2H, m, H-12, H-6), 2.100 (1H, m, H-8), 2.043 (1H, d, *J =* 14.6 Hz, H-10), 1.570-1.684 (3H, m, H-12, H-10), 1.309 (3H, s, H-18), 1.252 (3H, brd, *J =* 7.2 Hz*,* H-16), 1.166 (3H, s. H-17), 1.018 (3H, s, H-20), 1.006 (3H, s, H-19) (Figure S42 from Supplementary Materials). HRESIMS *m*/*z* 541.2541 [M + Na]^+^ (calcd for C_29_H_37_O_4_F_3_Na, 541.253615, Δ = 0.9 ppm) (Figure S44).

(*R*)-MTPA ester of **1** (**3b**): ^1^H NMR (CDCl_3_, 500.13 MHz) 6.987 (1H, q, *J =* 10.1, 5.9Hz, H-3), 5.977 (1H, dd, *J =* 10.1, 0.9 Hz, H-2), 5.106 (1H, t, *J =* 8.7 Hz, H-13), 3.536 (3H, d, *J =* 0.9 Hz, OMe), 2.697 (1H, m, H-4), 2.267-2.224 (2H, m, H-12, H-6), 2.100 (1H, m, H-8), 2.043 (1H, d, *J =* 14.6 Hz, H-10), 1.730 (1H, q, *J =* 14.0, 9.3 Hz, H-12), 1.589-1.691 (2H, m, H-10), 1.349 (3H, s, H-18), 1.259 (3H, btd, *J =* 7.2 Hz*,* H-16), 1.168 (3H, s. H-17), 0.964 (3H, s, H-20), 0.915 (3H, s, H-19) (Figure S44 from Supplementary Materials). HRESIMS *m*/*z* 541.2542 [M + Na]^+^ (calcd for C_29_H_37_O_4_F_3_Na, 541.253615, Δ *=* 0.9 ppm) (Figure S41).

### 4.9. The Quantum Chemical Calculations

The quantum chemical modeling of geometry and spectroscopic properties of compounds **1** and **4** was performed using Gaussian 16 package of programs [49]. Geometry optimizations and calculations IR spectra were performed with B3LYP exchange-correlation functional, the polarization continuum model (PCM), and 6-311G(d) split-valence basis set using default algorithms.

Two important conformations of **1** were found; their statistical weights (g*_im_*) were calculated via Gibbs free energies:

(1) gim=e−ΔGim/RT∑ie−ΔGim/RT

where index “*m*” denotes the most stable conformation and Δ*G_im_* = *G_i_* − *G*_m_ are the relative Gibbs free energies. The contributions of translational, rotational, vibrational, and electronic motions to *G_im_* were calculated at temperature T = 298.15 K. 

The ECD spectra were calculated using time-dependent density functional theory (TDDFT), cam-B3LYP functional, PCM model, and 6-311G(d) basis set. Twenty-five electronic transitions were calculated for each conformation of **1** and **4**. The individual bands in theoretical spectra were simulated as Gauss-type functions with the bandwidths ζ = 0.44 eV. The UV shifts Δλ = 17 nm and 14 nm were used for best correspondence between experimental and calculated spectra for compounds **1** and **4**, respectively.

### 4.10. Antimicrobial Activity

The yeast-like fungi of *Candida albicans* KMM 455 and bacterial strains *Staphylococcus aureus* ATCC 21027 and *Escherichia coli* VKPM (B-7935) (Collection of Marine Microorganisms PIBOC FEB RAS) were cultured on solid medium Mueller Hinton broth with agar (16.0 g/L) in a Petri dish at 37 °C for 24 h. 

The assays were performed in 96-well microplates in appropriate Mueller Hinton broth. Each well contained 90 µL of bacterial or of yeast-like fungi suspension (10^9^ CFU/mL). Then, 10 µL of a compound diluted at concentrations from 1.5 µM to 100.0 µM using two-fold dilution were added (DMSO concentration <1%). Culture plates were incubated for 18 h at 37 °C, and the OD_620_ was measured using a Multiskan FS spectrophotometer (Thermo Scientific Inc., Beverly, MA, USA). The antibiotic gentamicin and the antifungal agent nitrofungin were used as positive controls at 1 mg/mL; 1% DMSO in PBS served as a negative control [50]. 

### 4.11. Biofilm Formation Assay

The inhibition reducing biofilm formation and growth was assessed using the crystal violet biofilm assay as described [24]. Overnight cultures of bacteria *S. aureus* and *E. coli* and yeast-like fungi *C. albicans* were inoculated into Mueller–Hinton broth at a concentration of 10^9^ CFU/mL. A total of 90µL of this cell suspension was then dispensed into 96-well microtiter plates containing 10 µL of different concentrations of compounds **1**–**6**. After 24 h growth at 37 °C, the plates were washed with PBS to remove unbound cells. Next, the wells were stained with 0.1% crystal violet solution for 10 min at 37 °C. At the completion of the incubation, plates were washed 3 times with PBS and dried. Then, the crystal violet dye from the biofilm was solubilized with 100 µL of ethanol. A total of 100 µL of this solution was then moved to a new microtiter plate for absorbance measurement at λ = 570 nm. The results were reported as percent inhibition normalized to the wild-type control.

### 4.12. Sortase A Activity Inhibition Assay

The enzymatic activity of sortase A from *S. aureus* was determined using SensoLyte 520 Sortase A Activity Assay Kit Fluorimetric (AnaSpec AS-72229, AnaSpec, San Jose, CA, USA) in accordance with the manufacturer’s instructions. The compounds were dissolved in DMSO and diluted with reaction buffer to obtain a final concentration of 0.8% DMSO, which did not affect enzyme activity. DMSO at a concentration of 0.8% was used as a control. 4-(Hydroxymercuri)benzoic acid (PCMB) was used as sortase A enzyme activity inhibitor. Fluorescence was measured with a plate reader PHERAStar FS (BMG Labtech, Offenburg, Germany) for 60 min, with a time interval of 5 min. The data were processed with MARS Data Analysis v. 3.01R2 (BMG Labtech, Offenburg, Germany). The results were presented as relative fluorescent units (RFUs) and percentage of the control data, calculated using STATISTICA 10.0 software.

### 4.13. Cell Culture

The rat cardiomyocytes H9c2 cells were kindly provided by Prof. Dr. Gunhild von Amsberg from Martini-Klinik Prostate Cancer Center, University Hospital Hamburg-Eppendorf, Hamburg, Germany. 

The H9c2 cells were cultured in DMEM medium (Biolot, St. Petersburg, Russia) containing 10% fetal bovine serum (Biolot, St. Petersburg, Russia) and 1% penicillin/streptomycin (Biolot, St. Petersburg, Russia) at 37 °C in a humidified atmosphere with 5% (*v*/*v*) CO_2_. 

### 4.14. Cell Viability Assay

The H9c2 cells were seeded at concentrations of 3 × 10^3^ cell/well, and the experiments were started after 24 h. The compounds at concentrations up to 100 µM were added into the wells for 24 h, and the viability of the cells was measured with an MTT (3-(4,5-dimethylthiazol-2-yl)-2,5-diphenyltetrazolium bromide) assay, which was performed according to the manufacturer’s instructions (Sigma-Aldrich, Munich, Germany). All compounds were dissolved with DMSO so that the final concentration of DMSO in the cell culture was not more than 1%. Moreover, DMSO was used as a control. The results were presented as a percent of the control data and calculated IC_50_.

### 4.15. Molecular Docking

The pdb file of sortase A (PDB ID 1T2P) was obtained from the RCSB Protein Data Bank (https://www.rcsb.org, accessed on 8 November 2023) and prepared for docking with the PrepDock package of UCFS Chimera 1.16 software. The chemical structures of ligands were prepared for docking with ChemOffice and checked with the PrepDock package of UCFS Chimera 1.16 software.

Docking was conducted on the SwissDock online server (http://www.swissdock.ch, accessed on 8 November 2023) based on EADock DSS docking software [51]. The algorithm implies the generation of many binding modes in the vicinity of all target cavities (blind docking) and estimation of their CHARMM energies via the Chemistry at HARvard Macromolecular Mechanics (CHARMM) algorithm [52] for evaluation of the binding modes with the most favorable energies with FACTS (Fast Analytical Continuum Treatment of Solvation) [53] and, finally, clustering of these binding modes [54].

The predicted building models for each target/ligand pair were visualized and analyzed with UCFS Chimera 1.16 software. Docking parameters, such as Gibb’s free energy (ΔG, kcal/mol), FullFitness score (FF, kcal/mol), and hydrogen-bonding and hydrophobic interactions, were used for analysis of target/ligand complexes.

### 4.16. Statistical Data Evaluation

All data were obtained in three independent replicates, and calculated values were expressed as a mean ± standard error mean (SEM). Student’s *t*-test was performed using SigmaPlot 14.0 (Systat Software Inc., San Jose, CA, USA) to determine statistical significance. Differences were considered statistically significant at *p* < 0.05.

## 5. Conclusions

Two new cyclopiane diterpenes and a new cladosporin precursor, together with four known related compounds, were isolated from marine sediment-derived fungus *P. antarcticum* KMM 4670. The absolute stereostructures of conidiogenone F and leptosphin C were determined for the first time. This study is the very first report of the isolation of cladosporin precursors **5** and **6** as individual natural compounds. New cyclopiane diterpene 13-*epi*-conidiogenone F (**2**) is very promising for future investigation as an anti-staphylococcal agent.

## Figures and Tables

**Table 1:** NMR data (700 MHz, CDCl_3_, δ in ppm) for compounds **1** and **2**.

Position	1		2	
	δ_C_, Type	δ_H_, Mult (*J* in Hz)	δ_C_, Type	δ_H_, Mult (*J* in Hz)
1	205,0 C		206.1, C	
2	126.1, CH	5.86, d (10.2)	127.7, CH	5.97, d (10.0)
3	157.0, CH	6.68, d (10.3)	154.8, CH	6.93, dd (10.0, 5.9)
4	74.2, C		38.3, CH	2.96, m
5	65.1, C		60.5, C	
6	52.0, CH	2.79, dd (9.3, 5.0)	55.1, CH	2.52, m
7	34.5, CH_2_	α: 1.62, m β: 1.23, m	35.1, CH_2_	α: 1.64, m β: 1.19, m
8	40.0, CH_2_	α: 2.12, m β: 1.75, td (13.7, 7.0)	39.8, CH_2_	α: 1.67, m β: 2.08, dd (11.0, 5.3)
9	58.2, C		57.8, C	
10	48.8, CH_2_	α: 2.27, d (15.2) β: 1.87, d (15.0)	47.5, CH_2_	α: 2.03, d (14.7) β: 1.68, d (14.7)
11	44.4, C		48.7, C	
12	51.5, CH_2_	α: 2.85, d (18.8) β: 2.33, d (18.8)	48.5, CH_2_	α: 1.73, dd (13.4, 7.1) β: 1.98, dd (13.4, 6.5)
13	224.9, C		82.8, CH	3.92, t (6.8)
14	50.7, C		45.2, C	
15	72.3, CH	1.86, d (5.1)	73.6, CH	1.51, d (5.3)
16	29.1, CH_3_	1.41, s	19.0, CH_3_	1.16, d (7.1)
17	20.6, CH_3_	1.21, s	21.4, CH_3_	1.21, s
18	34.3, CH_3_	1.41, s	32.1, CH_3_	1.22, s
19	21.7, CH_3_	1.08, s	21.1, CH_3_	0.94, s
20	30.1, CH_3_	1.11, s	31.5, CH_3_	1.05, s

**Table 2:** NMR data (700 MHz, δ in ppm) for compounds **5** and **6**.

Position	5 ^a^		6 ^b^	
	δ_C_, Mult	δ_H_ (*J* in Hz)	δ_C_, Mult	δ_H_ (J in Hz)
1	174.9, C		172.5, C	
2	41.2, CH_2_	a: 2.56, brd (5.3) b: 2.57, brd (6.8)	39.1, CH_2_	a: 2.43, dd (14.9, 6.0) b: 2.58, dd (14.9, 7.8)
3	65.9, CH	4.29, m	68.7, CH	4.19, m
4	39.4, CH_2_	a: 1.58, m b: 1.87, ddd (14.4, 9.0, 3.4)	30.3, CH_2_	a: 1.37, m b: 1.67, m
5	67.8, CH	4.08, m	18.9, CH_2_	1.65, m
6	30.6, CH_2_	a: 1.42, m b: 1.62, m	32.2, CH_2_	a: 1.26, m b: 1.64, m
7	18.3, CH_2_	a: 1.64, m b: 1.72, m	67.7 CH	3.90, m
8	30.8, CH_2_	a: 1.37, m b: 1.72, m	20.0, CH_3_	1.12, d (6.4)
9	68.3, CH	4.05, m		
10	18.6, CH_3_	1.23, d (6.5)		

^a^ CDCl_3_; ^b^ acetone-d_6_.

**Table 3:** The results of molecular docking of compounds **1**–**4** with sortase A structure (PDB ID 1T2P).

Compound	FF Score, kcal/mol	∆G, kcal/mol	Hydrogen-Bonding Interactions	Hydrophobic Interactions
**1**	−3553.963	−7.4239464	Arg197 … O (at C-4), 2.226 Å	Ala104, Ile182, Ala92, Thr93
	−3549.677	−6.722709	Arg197 … O2 (at C-13), 2.234 Å OH-group at C-4 … Glu105, 1.876 Å	Ile199, Ile182,
**2**	−3531.3604	−6.4623137	Arg197 … O (at C-1), 2.681 Å OH-group at C-13 … Glu105, 2.118 Å	Gly192, Val193, Ala104
	−3541.7644	−7.16274	OH-group at C-13 … Glu105, 2.097 Å	Ala92, Thr93, Thr187, Trp194, Ala104, Gly192
**3**	−3539.8362	−6.2875967	Arg197 … OH-group at C-13, 2.668 Å	Ala104, Ile182,
**4**	−3548.0125	−6.429369	Arg197 … O (at C-13), 2.493 Å	Ile182
	−3549.6343	−7.032929	no	Ala92, Gly192, Ile182, Ala104

**Table 4:** The effect of compounds **1**–**6** on the viability of H9c2 cardiomyocytes.

Compound	Cell Viability, %		
	1 µM	10 µM	100 µM
**1**	94.8 ± 5.9	65.7 ± 9.4	63.2 ± 0.5
**2**	98.1 ± 1.9	107.3 ± 7.8	67.3 ± 3.1
**3**	103.3 ± 3.2	92.9 ± 2.5	109.7 ± 9.2
**4**	107.2 ± 2.9	101.7 ± 4.6	92.3 ± 1.9
**5**	94.4 ± 3.1	88.5 ± 3.1	74.1 ± 4.9
**6**	98.1 ± 1.6	80.9 ± 2.9	54.6 ± 2.1

**Table 5:** The strains of the species used in multi-locus phylogenetic analysis and GenBank accession numbers.

Species	Strain Number	GenBank Accession Number			
		*ITS*	*BenA*	*CaM*	*RPB2*
*Penicillium atrovenetum* G. Smith	CBS 241.56 ^T^	AF033492	JX140944	KJ867004	JN121467
*Penicillium antarcticum* A.D. Hocking & C.F. McRae	CBS 100492 ^T^	KJ834503	MN969371	MN969236	JN406653
*Penicillium antarcticum*	**KMM 4670**	**KU358553**	**KU358556**	**KU358559**	**OR271597**
*Penicillium coralligerum* Nicot and Pionnat	CBS 123.65 ^T^	JN617667	MN969378	MN969248	JN406632
*Penicillium nucicola* Visagie, Malloch, and Seifert	CBS 140987 ^T^	KT887860	KT887821	KT887782	MN969171
*Penicillium novae-zeelandiae* J.F.H. Beyma	CBS 137.41 ^T^	JN617688	MN969390	MN969279	JN406628
*Aspergillus glaucus* Link	NRRL 116 ^T^	EF652052	EF651887	EF651989	EF651934

^T^—ex-type strain.

## Data Availability

The original data presented in the study are included in the article/Supplementary Materials; further inquiries can be directed to the corresponding author.

## Supplementary Materials

The following supporting information can be downloaded at: https://www.mdpi.com/article/10.3390/md21110584/s1, Figures S1–S27: NMR spectra of compounds **1**–**6**; Figures S38–S43: MS spectrum of compounds **1**–**6**; Figures S28, S30, S32, S34, S36: UV spectra of compounds **1**–**5;** Figures S29, S31, S33, S35, S37: CD spectra of compounds **1**–**5**; Figures S44–S45, S47-S48: NMR spectra of compounds **3a** and **3b**; Figures S46, S49: MS spectrum of compounds **3a** and **3b.**
