# Supplementary material for: New Cyclopiane Diterpenes and Polyketide Derivatives from Marine Sediment-Derived Fungus Penicillium antarcticum KMM 4670 and Their Biological Activities
Source: Mar Drugs. 2023 Nov 9;21(11):584. doi: 10.3390/md21110584 (PMC10672241; doi:10.3390/md21110584)

SUPPLEMENTARY MATERIAL

# New antimicrobial cyclopiane diterpenes and polyketide derivatives from marine sediment-derived fungus *Penicillium antarcticum* KMM 4670

**Content**

Figure S1. $^{1}$H NMR spectrum (700 MHz, $CDCl_3$) of 1 ........ 4
Figure S2. $^{13}$C NMR spectrum (125.75 MHz, $CDCl_3$) of 1 ........ 6
Figure S3. DEPT NMR spectrum (700 MHz, $CDCl_3$) of 1 ........ 7
Figure S4. HSQC NMR spectrum (700 MHz, CDCl3) of 1 ........ 8
Figure S5. HMBC NMR spectrum (700 MHz, CDCl3) of 1 ........ 9
Figure S6. $^{1}$H-$^{1}$H COSY NMR spectrum (700 MHz, CDCl3) of 1 ........ 10
Figure S7. ROESY NMR spectrum (700 MHz, CDCl3) of 1 ........ 11
Figure S8. $^{1}$H NMR spectrum (700 MHz, CDCl3) of 2 ........ 12
Figure S9. $^{13}$C NMR spectrum (125 MHz, CDCl3) of 2 ........ 14
Figure S10. DEPT NMR spectrum (700 MHz, CDCl3) of 2 ........ 15
Figure S11. HSQC NMR spectrum (700 MHz, CDCl3) of 2 ........ 16
Figure S12. HMBC NMR spectrum (700 MHz, CDCl3) of 2 ........ 17
Figure S13. $^{1}$H-$^{1}$H COSY NMR spectrum (700 MHz, CDCl3) of 2 ........ 18
Figure S14. ROESY NMR spectrum (700 MHz, CDCl3) of 2 ........ 19
Figure S15. $^{1}$H NMR spectrum (700 MHz, CDCl3) of 3 ........ 20
Figure S16. $^{13}$C NMR spectrum (700 MHz, CDCl3) of 3 ........ 23
Figure S17. $^{1}$H NMR spectrum (700 MHz, CDCl3) of 4 ........ 24
Figure S18. $^{13}$C NMR spectrum (700 MHz, CDCl3) of 4 ........ 26
Figure S19. $^{1}$H NMR spectrum (500 MHz, $CDCl_3$) of 5 ........ 27
Figure S20. $^{13}$C NMR spectrum (500 MHz, $CDCl_3$) of 5 ........ 28
Figure S21. DEPT NMR spectrum (500 MHz, $CDCl_3$) of 5 ........ 29
Figure S22. HSQC NMR spectrum (500 MHz, $CDCl_3$) of 5 ........ 30
Figure S23. HMBC NMR spectrum (500 MHz, $CDCl_3$) of 5 ........ 31
Figure S24. $^{1}$H-$^{1}$H COSY NMR spectrum (500 MHz, $CDCl_3$) of 5 ........ 32
Figure S25. NOESY NMR spectrum (500 MHz, $CDCl_3$) of 5 ........ 33
Figure S26. $^{1}$H NMR spectrum (500 MHz, acetone-$d_6$) of 6 ........ 34
Figure S27. $^{13}$C NMR spectrum (500 MHz, acetone-$d_6$) of 6 ........ 36
Figure S28. UV spectrum of 1 ........ 37
Figure S29. CD spectrum of 1 ........ 37
Figure S30. UV spectrum of 2 ........ 38
Figure S31. CD spectrum of 2 ........ 38
Figure S32. UV spectrum of 3 ........ 39
Figure S33. CD spectrum of 3 ........ 39
Figure S34. UV spectrum of 4 ........ 40
Figure S35. The experimental and calculated ECD spectra of 4 ........ 40
Figure S36. UV spectrum of 5 ........ 41
Figure S37. CD spectrum of 5 ........ 41
Figure S38. HR (+)ESI MS spectrum of 1 ........ 42
Figure S39. HR (+)ESI MS spectrum of 2 ........ 42
Figure S40. HR (+)ESI MS spectrum of 3 ........ 42

Figure S41. HR (+)ESI MS spectrum of 4 .......... 43
Figure S42. HR (+)ESI MS spectrum of 5 .......... 43
Figure S43. HR (+)ESI MS spectrum of 6 .......... 43
Figure S44. $^{1}$H NMR spectrum for (*R*)-MTPA esters (500 MHz, CDCl3) of (3b) .......... 44
Figure S45. $^{1}$H-$^{1}$H COSY NMR spectrum for (*R*)-MTPA esters (500 MHz, CDCl3) of (3b) .......... 45
Figure S46. HR (+)ESI MS spectrum of for (*R*)-MTPA esters of (3b) .......... 46
Figure S47. $^{1}$H NMR spectrum for (*S*)-MTPA esters (500 MHz, CDCl3) of (3a) .......... 47
Figure S48. $^{1}$H-$^{1}$H COSY NMR spectrum for (*S*)-MTPA esters (500 MHz, CDCl3) of (3a) .......... 48
Figure S49. HR (+)ESI MS spectrum of for (*S*)-MTPA esters of (3a) .......... 49
Figure S50. Comparison of the experimental ECD spectra of compounds 1–4 .......... 49

**Figure S1. $^{1}H$ NMR spectrum (700 MHz, $CDCl_3$) of 1**

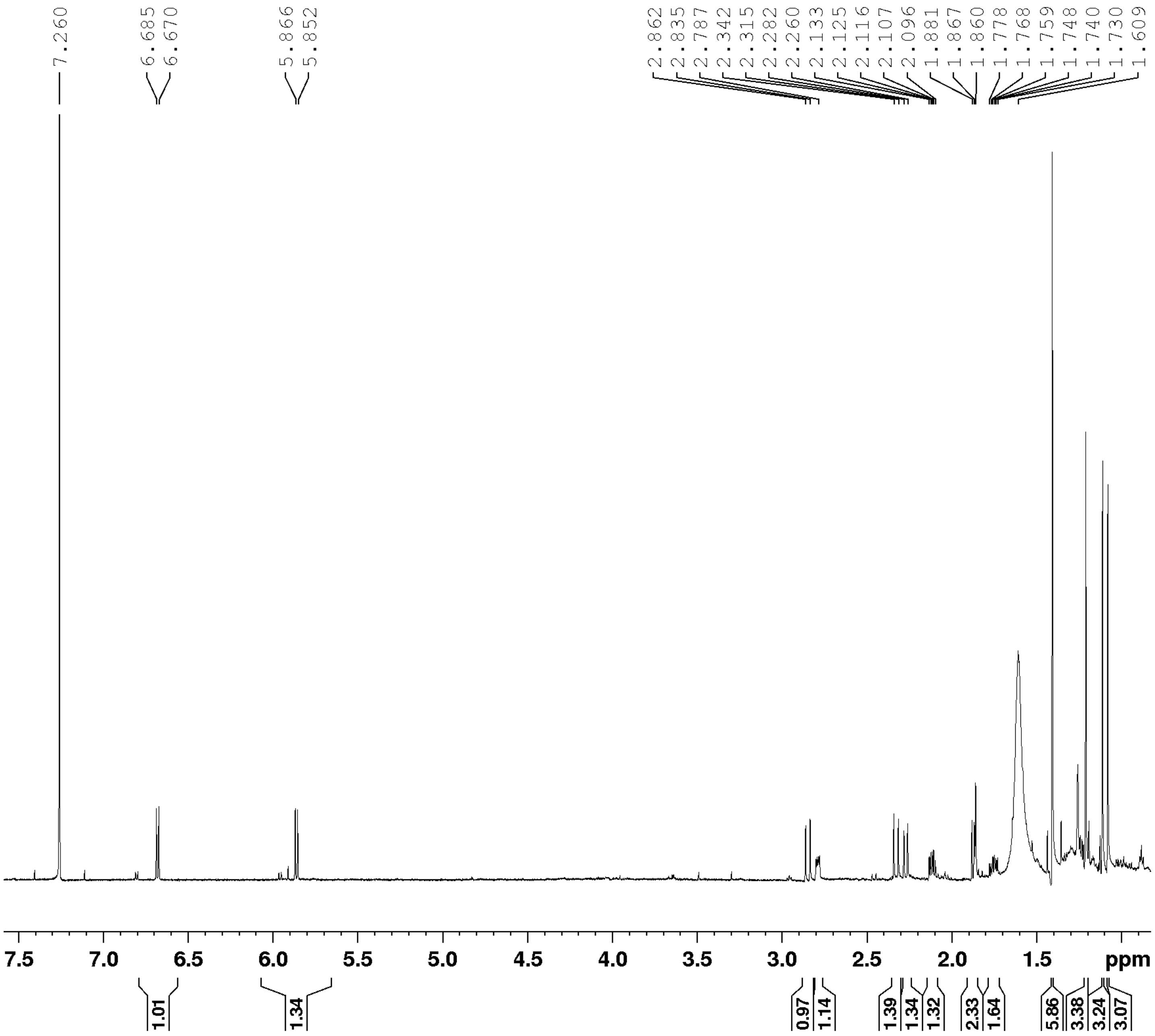


```
Current Data Parameters
NAME              CF-60ch
EXPNO                   1
PROCNO                  1

F2 - Acquisition Parameters
Date_            20220530
Time                12.00 h
INSTRUM             spect
PROBHD   Z134216_0006 (
PULPROG              zg30
TD                  32768
SOLVENT             CDCl3
NS                     16
DS                      2
SWH             10504.202 Hz
FIDRES           0.641126 Hz
AQ              1.5597568 sec
RG                 165.68
DW                 47.600 usec
DE                   6.50 usec
TE                  303.0 K
D1             2.00000000 sec
TD0                     1
SFO1          700.0051100 MHz
NUC1                   1H
P0                   5.33 usec
P1                  16.00 usec
PLW1          11.27900028 W

F2 - Processing parameters
SI                  32768
SF            700.0000162 MHz
WDW                    EM
SSB                     0
LB                   0.10 Hz
GB                      0
PC                   4.00
```

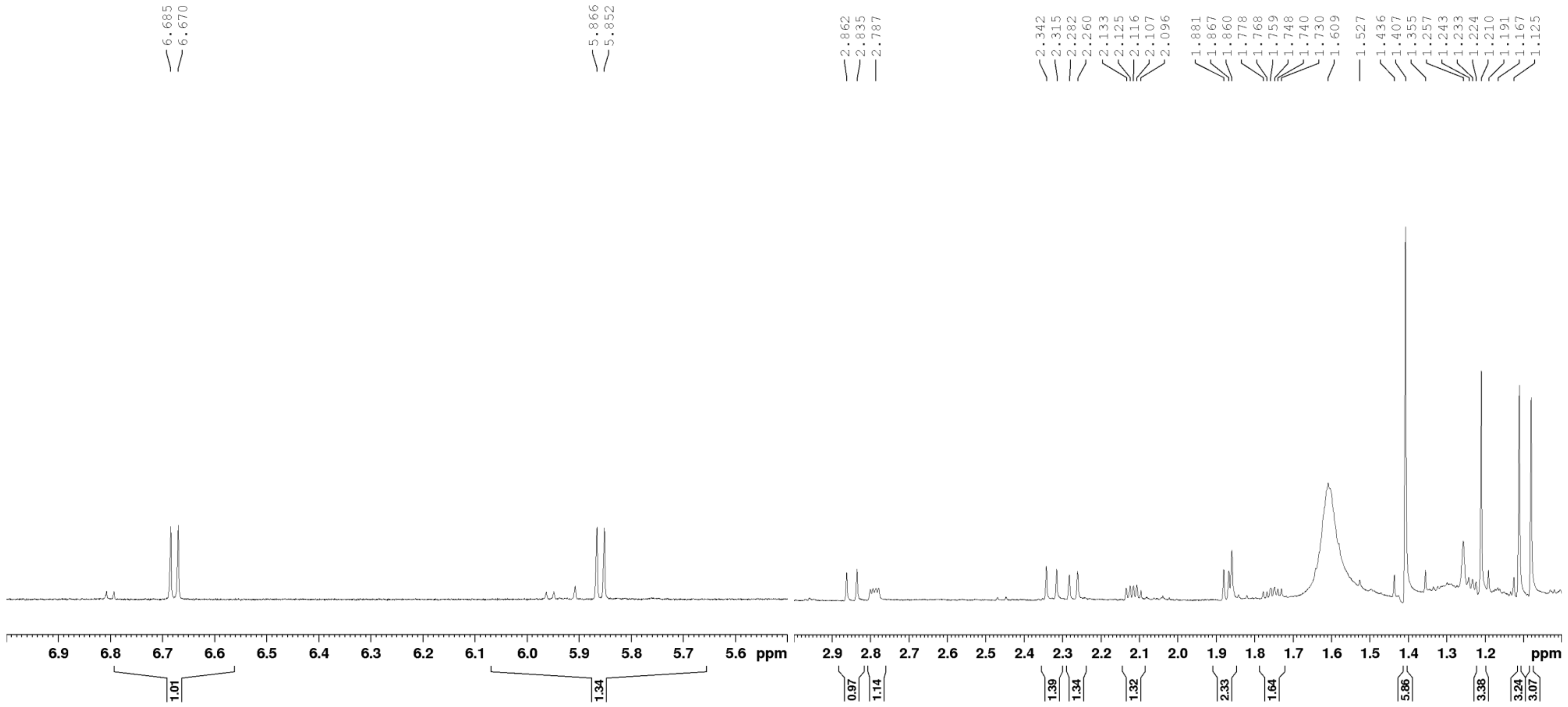

**Figure S2. $^{13}C$ NMR spectrum (125.75 MHz, $CDCl_3$) of 1**

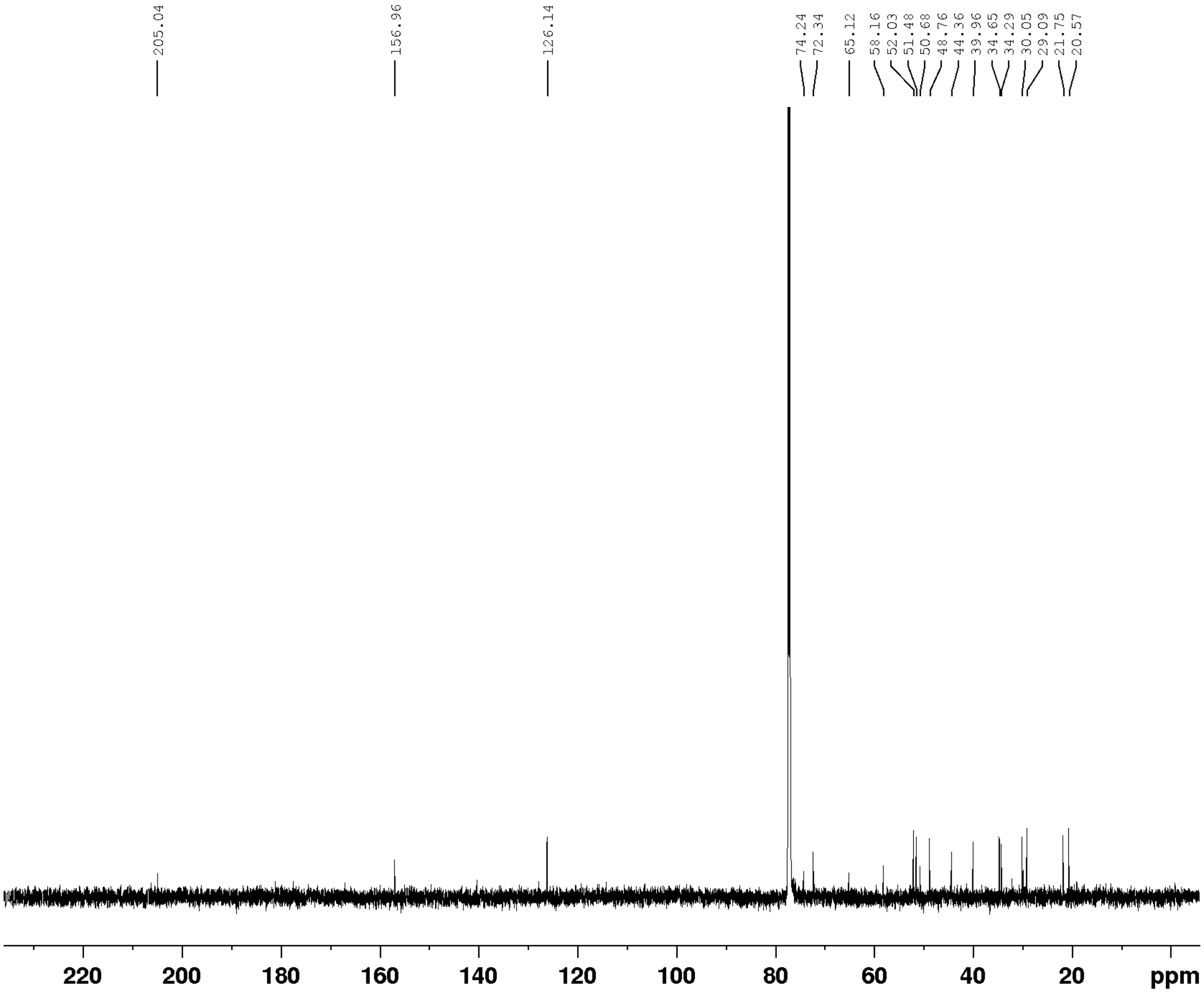


```
Current Data Parameters
NAME              CF-60ch
EXPNO                  14
PROCNO                  1

F2 - Acquisition Parameters
Date_            20220530
Time                17.37 h
INSTRUM             spect
PROBHD   Z134216_0006 (
PULPROG            zgpg30
TD                  32768
SOLVENT             CDCl3
NS                   7592
DS                      4
SWH             42613.637 Hz
FIDRES           2.600930 Hz
AQ              0.3844779 sec
RG                 191.01
DW                 11.733 usec
DE                   6.50 usec
TE                  303.0 K
D1             2.00000000 sec
D11            0.03000000 sec
TD0                  4096
SFO1          176.0353837 MHz
NUC1                  13C
P0                   4.00 usec
P1                  12.00 usec
PLW1          61.49700165 W
SFO2          700.0021000 MHz
NUC2                   1H
CPDPRG[2          waltz16
PCPD2               65.00 usec
PLW2          11.27900028 W
PLW12          0.68339998 W
PLW13          0.34412000 W

F2 - Processing parameters
SI                  65536
SF            176.0151108 MHz
WDW                    EM
SSB                     0
LB                   2.00 Hz
GB                      0
PC                   1.20
```

**Figure S3. DEPT NMR spectrum (700 MHz, $CDCl_3$) of 1**

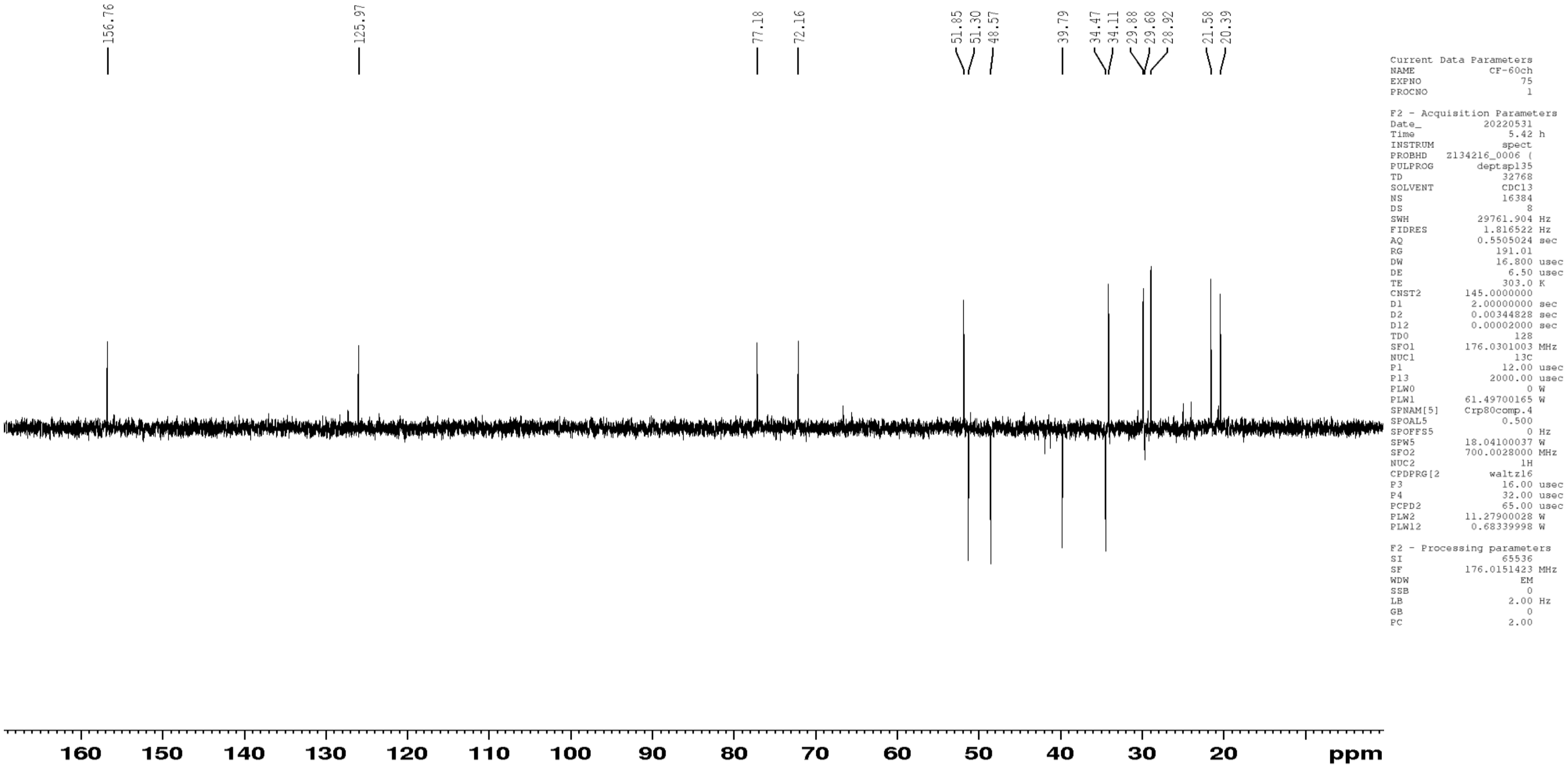

**Figure S4. HSQC NMR spectrum (700 MHz, CDCl3) of 1**

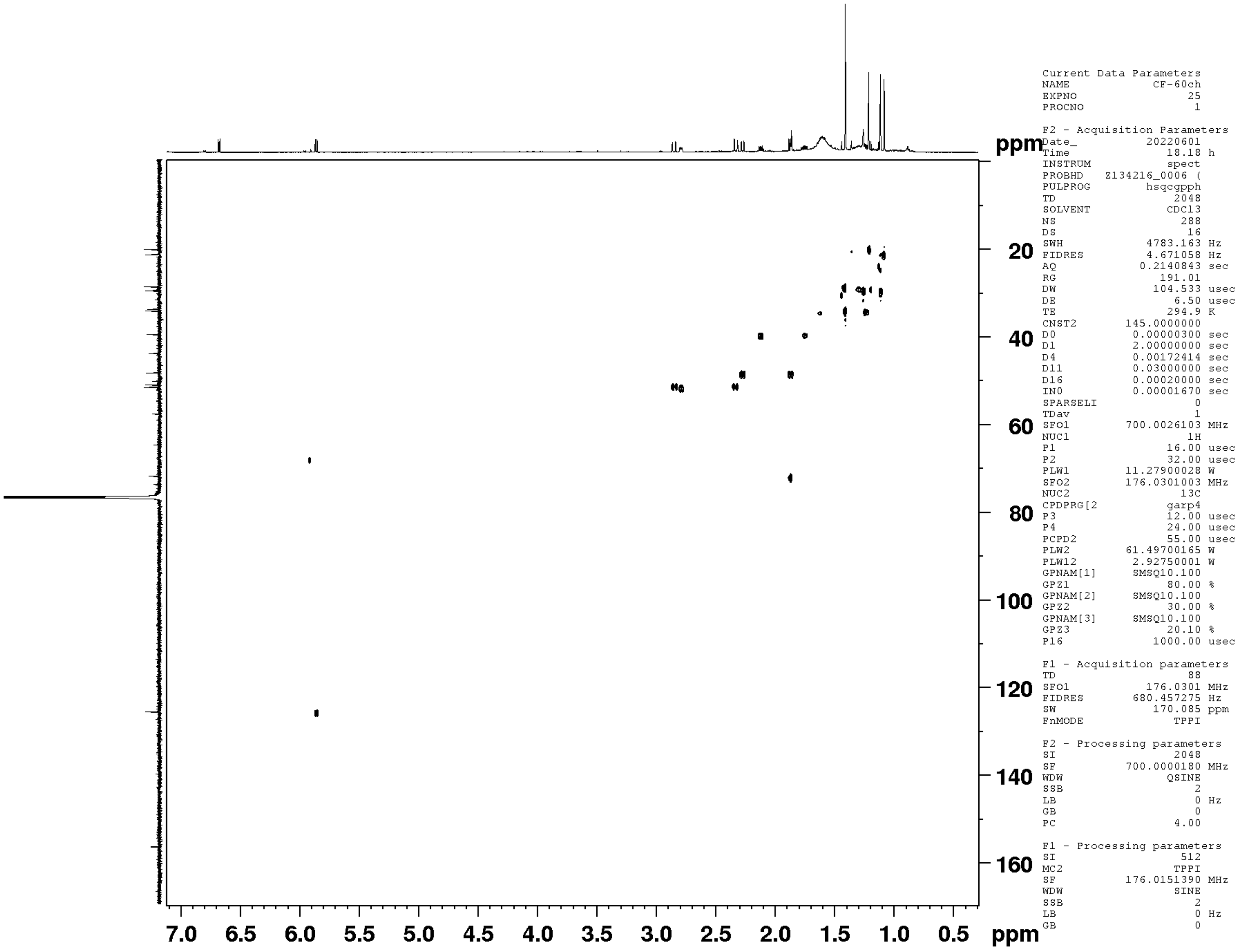

**Figure S5. HMBC NMR spectrum (700 MHz, CDCl3) of 1**

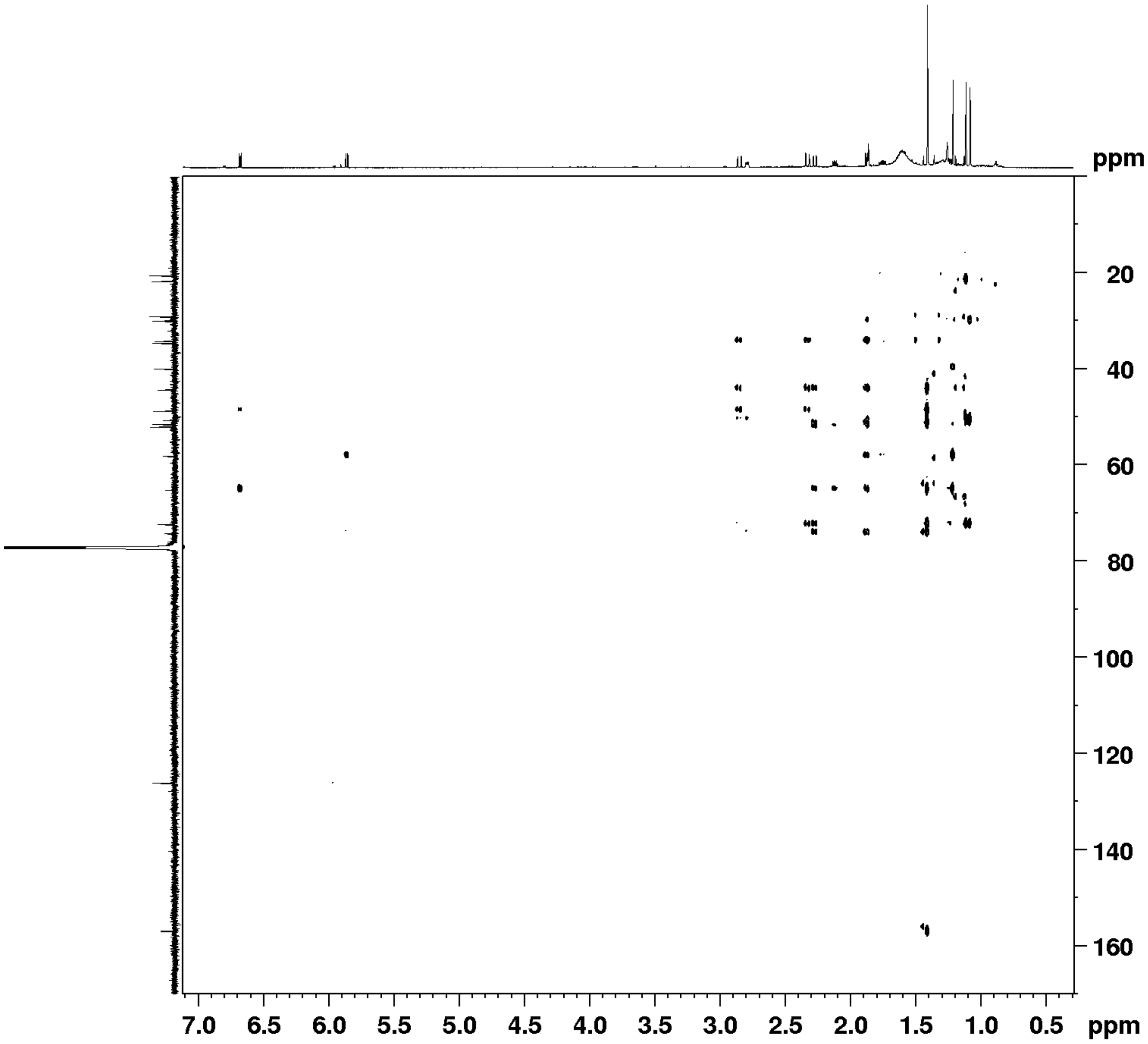


Current Data Parameters
NAME CF-60ch
EXPNO 26
PROCNO 1

F2 - Acquisition Parameters
Date_ 20220606
Time 15.33 h
INSTRUM spect
PROBHD Z134216_0006 (
PULPROG hmbcgplpndqf
TD 4096
SOLVENT CDCl3
NS 256
DS 16
SWH 4783.163 Hz
FIDRES 2.335529 Hz
AQ 0.4281685 sec
RG 191.01
DW 104.533 usec
DE 6.50 usec
TE 303.0 K
CNST2 145.0000000
CNST13 5.0000000
D0 0.00000300 sec
D1 2.00000000 sec
D2 0.00344828 sec
D6 0.10000000 sec
D16 0.00020000 sec
IN0 0.00001180 sec
SPARSELI 0
TDav 1
SFO1 700.0026103 MHz
NUC1 1H
P1 16.00 usec
P2 32.00 usec
PLW1 11.27900028 W
SFO2 176.0362608 MHz
NUC2 13C
P3 12.00 usec
PLW2 61.49700165 W
GPNAM[1] SMSQ10.100
GPZ1 50.00 %
GPNAM[2] SMSQ10.100
GPZ2 30.00 %
GPNAM[3] SMSQ10.100
GPZ3 40.10 %
P16 1000.00 usec

F1 - Acquisition parameters
TD 104
SFO1 176.0363 MHz
FIDRES 814.863098 Hz
SW 240.705 ppm
FnMODE QF

F2 - Processing parameters
SI 2048
SF 700.0000180 MHz
WDW SINE
SSB 3
LB 0 Hz
GB 0
PC 4.00

F1 - Processing parameters
SI 512
MC2 QF
SF 176.0151390 MHz
WDW SINE
SSB 0
LB 0 Hz
GB 0

**Figure S6. $^{1}$H-$^{1}$H COSY NMR spectrum (700 MHz, CDCl3) of 1**

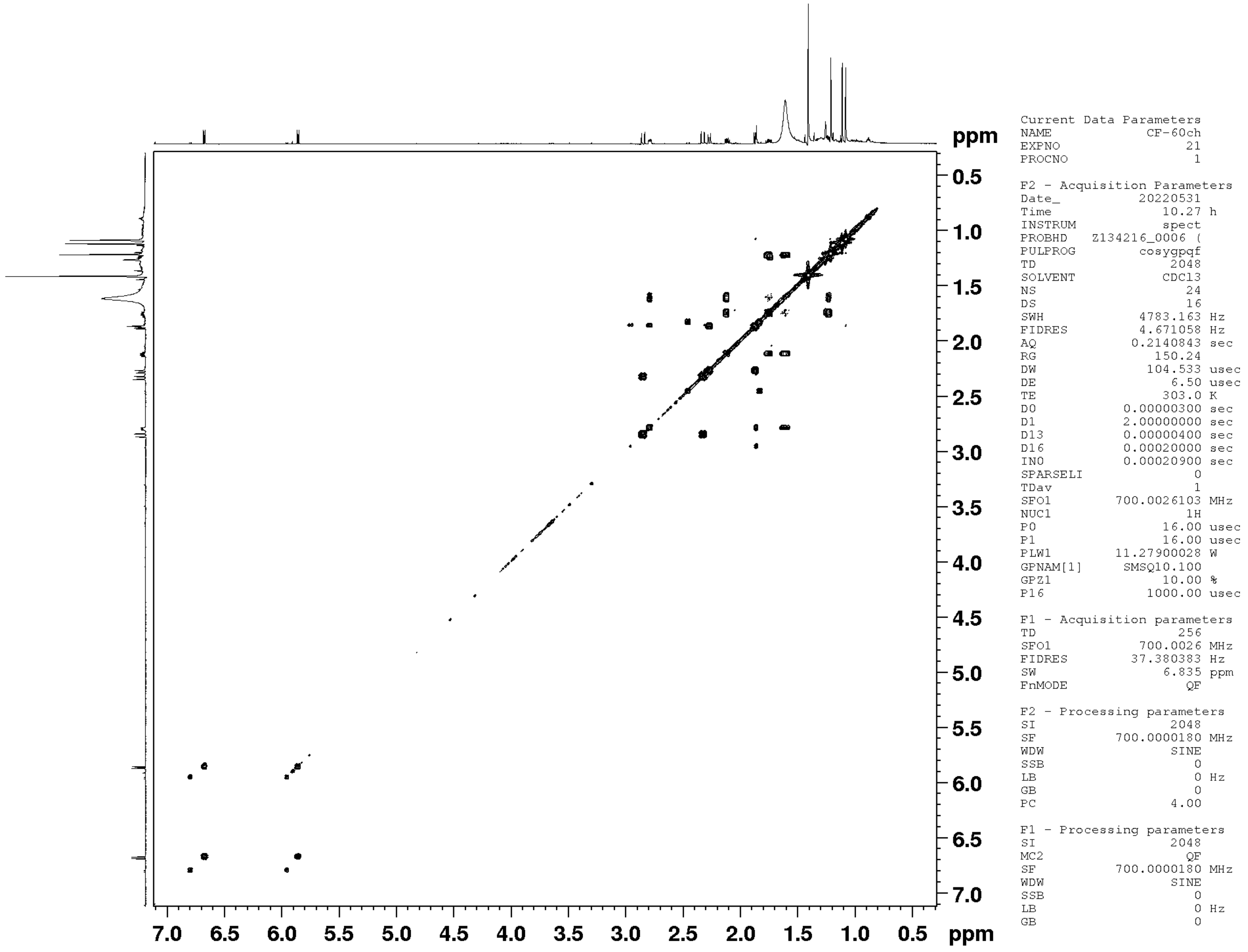

**Figure S7. ROESY NMR spectrum (700 MHz, CDCl3) of 1**

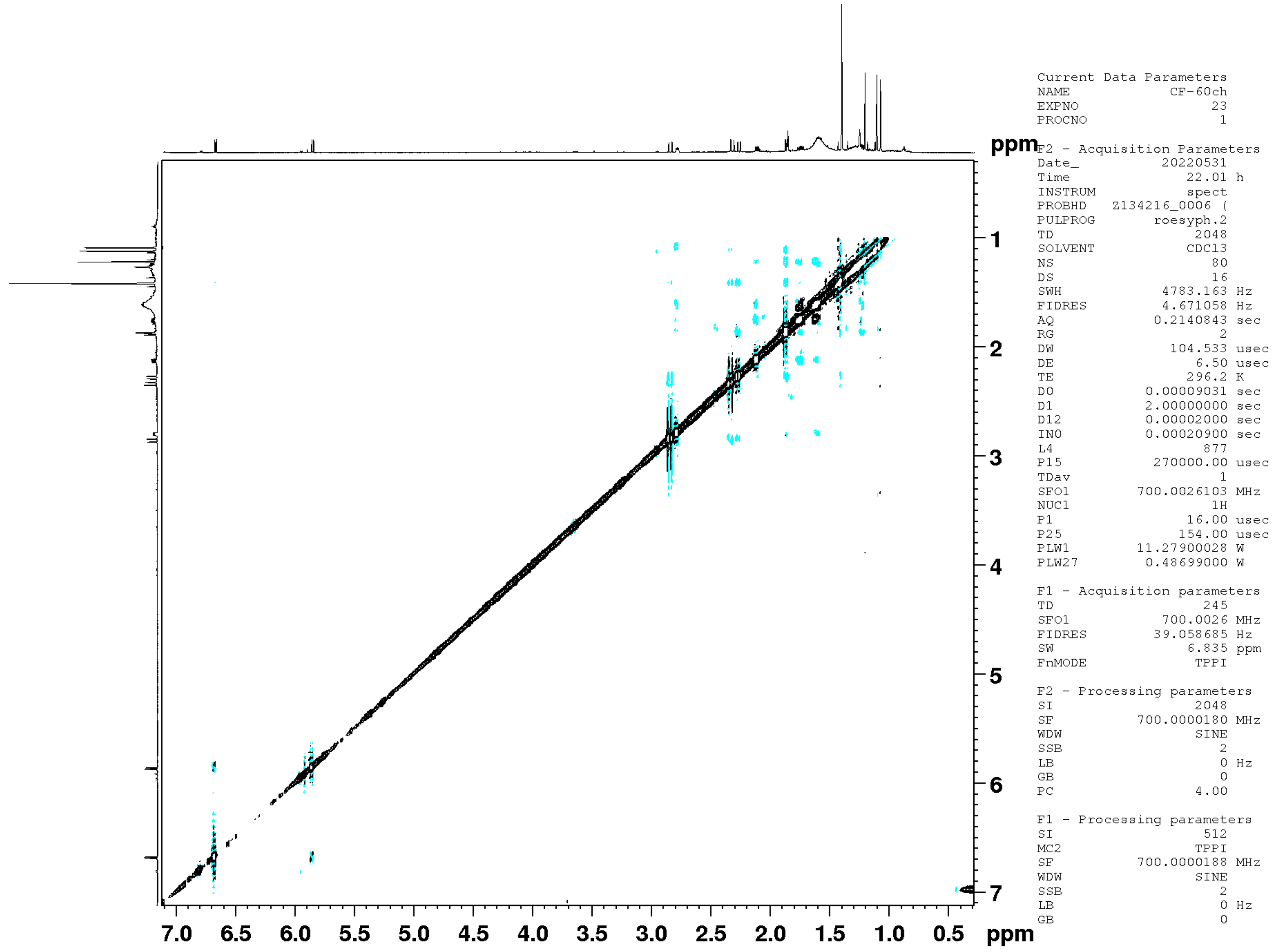

**Figure S8. $^{1}$H NMR spectrum (700 MHz, CDCl3) of 2**

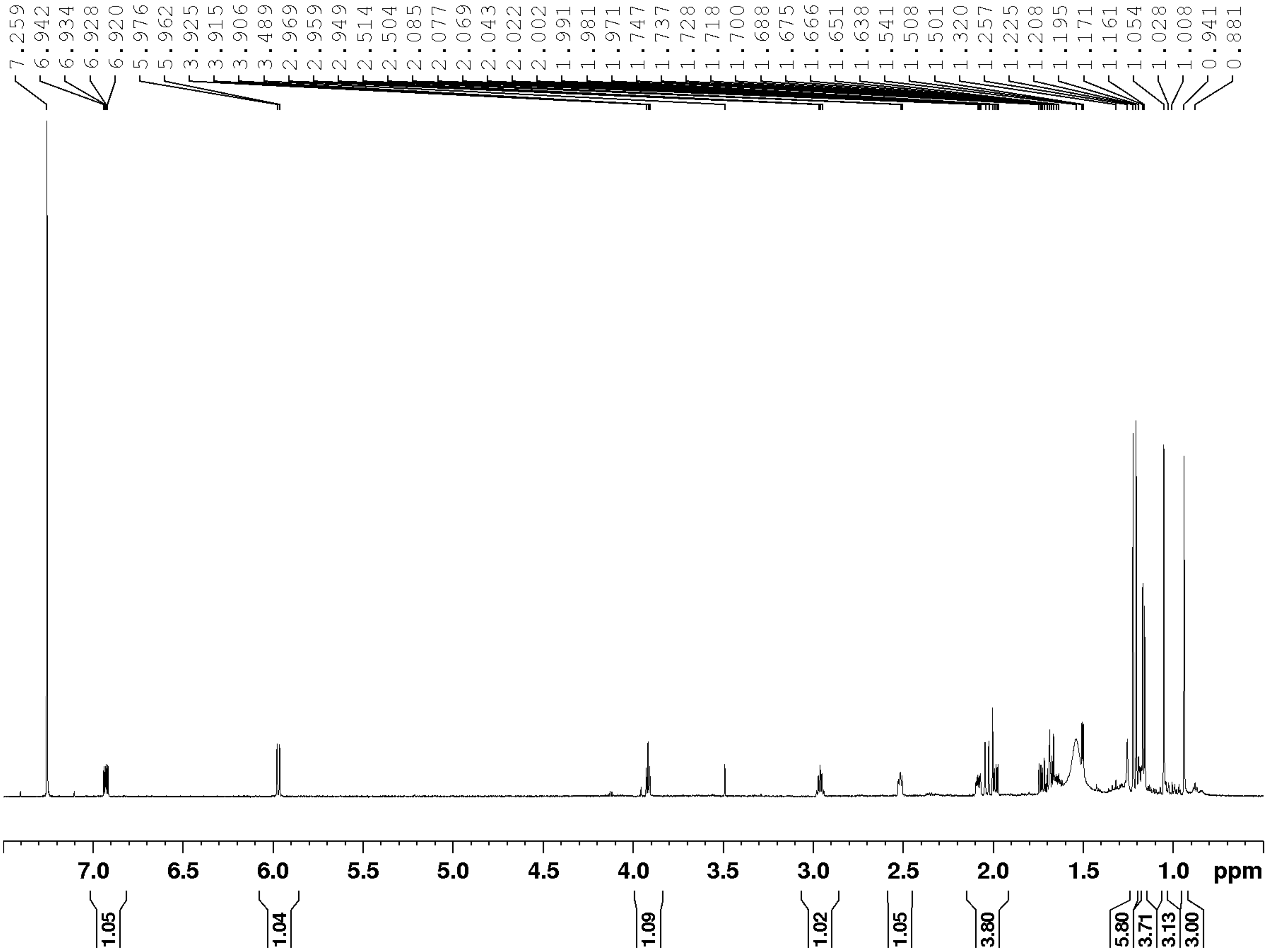


Current Data Parameters
NAME Och-67ch
EXPNO 2
PROCNO 1

F2 - Acquisition Parameters
Date_ 20220628
Time 16.33 h
INSTRUM spect
PROBHD Z134216_0006 (
PULPROG zg30
TD 32768
SOLVENT CDCl3
NS 8
DS 2
SWH 10504.202 Hz
FIDRES 0.641126 Hz
AQ 1.5597568 sec
RG 165.68
DW 47.600 usec
DE 6.50 usec
TE 302.9 K
D1 2.00000000 sec
TD0 1
SFO1 700.0051100 MHz
NUC1 1H
P0 5.33 usec
P1 16.00 usec
PLW1 11.27900028 W

F2 - Processing parameters
SI 32768
SF 700.0000178 MHz
WDW EM
SSB 0
LB 0.10 Hz
GB 0
PC 4.00

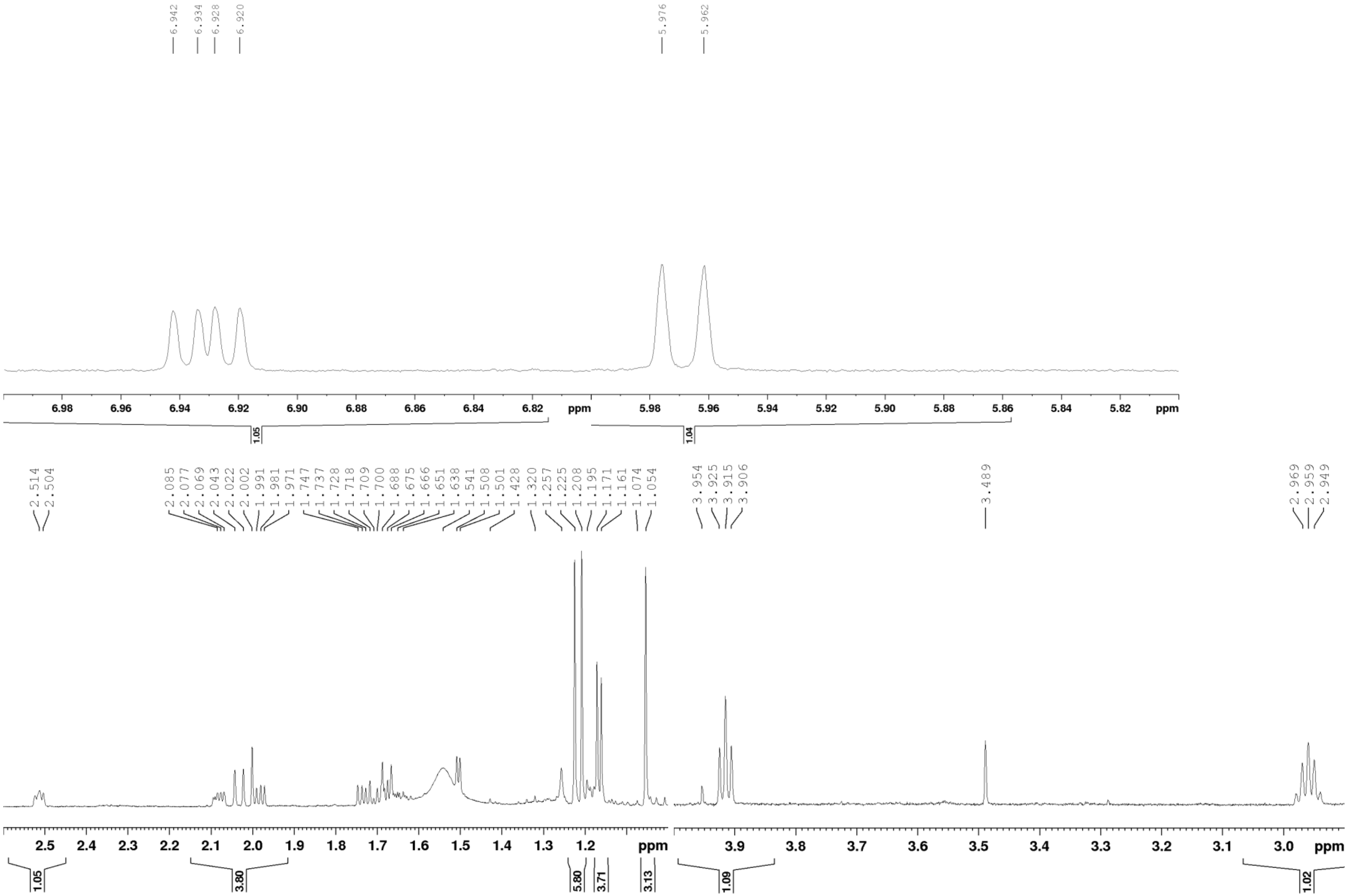
6.942
6.934
6.928
6.920
5.976
5.962
6.98 6.96 6.94 6.92 6.90 6.88 6.86 6.84 6.82 ppm
5.98 5.96 5.94 5.92 5.90 5.88 5.86 5.84 5.82 ppm
1.05
1.04
2.514
2.504
2.085
2.077
2.069
2.043
2.022
2.002
1.991
1.981
1.971
1.747
1.737
1.728
1.718
1.709
1.700
1.688
1.675
1.666
1.651
1.638
1.541
1.508
1.501
1.428
1.320
1.257
1.225
1.208
1.195
1.171
1.161
1.074
1.054
3.954
3.925
3.915
3.906
3.489
2.969
2.959
2.949
2.5 2.4 2.3 2.2 2.1 2.0 1.9 1.8 1.7 1.6 1.5 1.4 1.3 1.2 ppm
3.9 3.8 3.7 3.6 3.5 3.4 3.3 3.2 3.1 3.0 ppm
1.05
3.80
5.80
3.71
3.13
1.09
1.02

**Figure S9. $^{13}$C NMR spectrum (125 MHz, CDCl3) of 2**

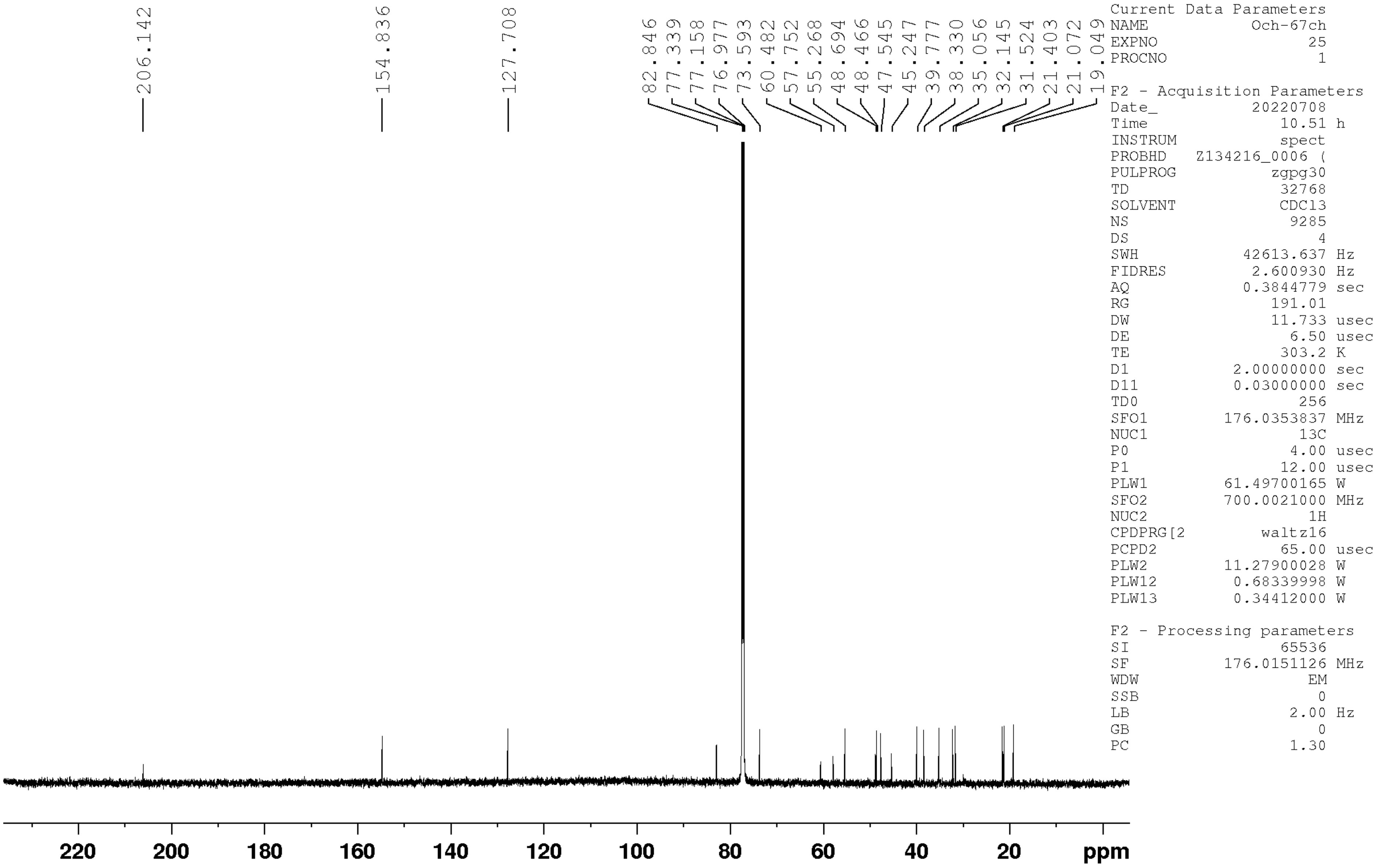

**Figure S10. DEPT NMR spectrum (700 MHz, CDCl3) of 2**

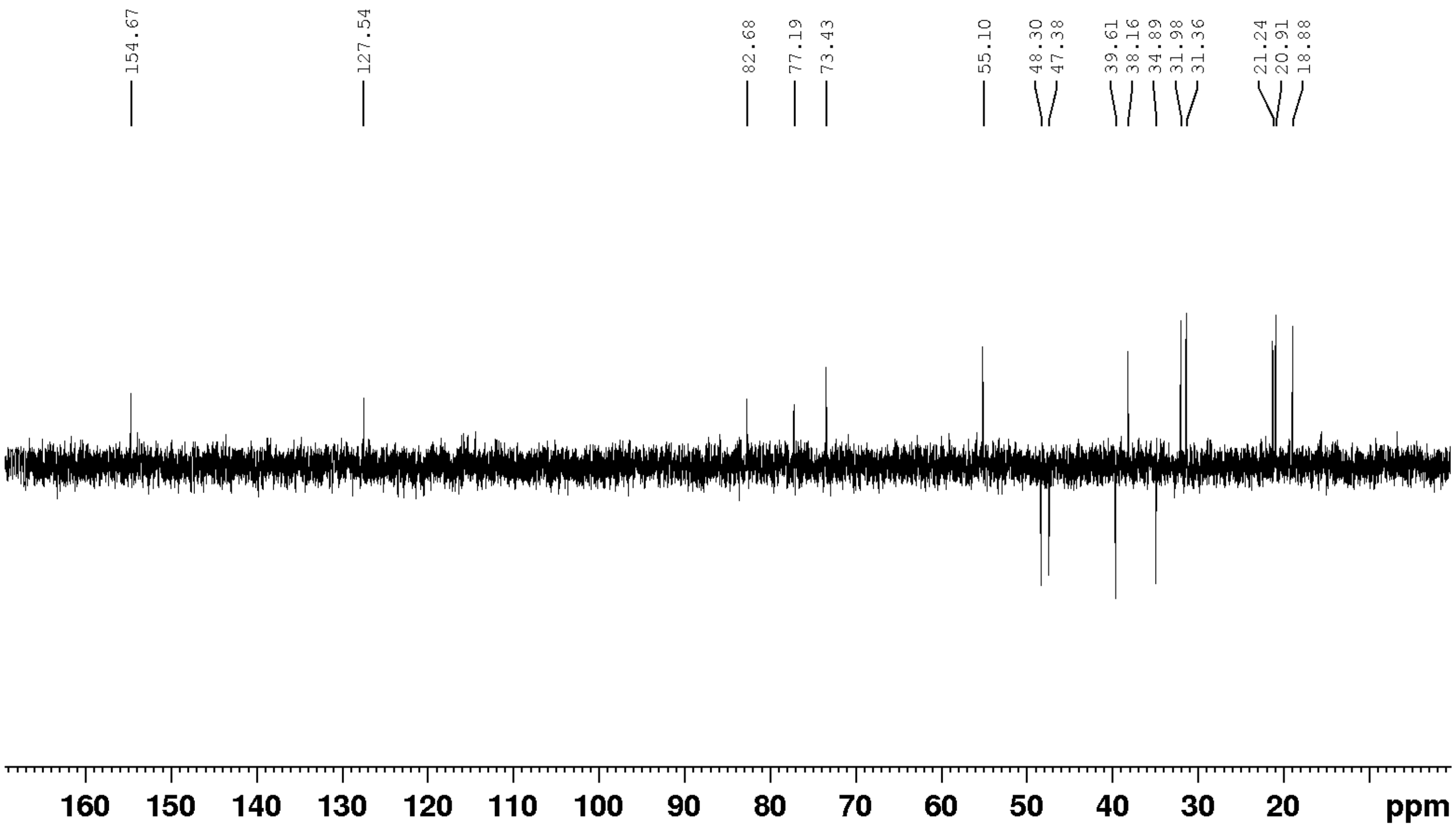


```
Current Data Parameters
NAME            Och-67ch
EXPNO                 75
PROCNO                 1

F2 - Acquisition Parameters
Date_           20220708
Time               11.42 h
INSTRUM            spect
PROBHD    Z134216_0006 (
PULPROG        deptsp135
TD                 65536
SOLVENT            CDCl3
NS                  1024
DS                     2
SWH            29761.904 Hz
FIDRES          0.908261 Hz
AQ             1.1010048 sec
RG                191.01
DW                16.800 usec
DE                  6.50 usec
TE                 303.1 K
CNST2        145.0000000
D1            1.00000000 sec
D2            0.00344828 sec
D12           0.00002000 sec
TD0                 4096
SFO1         176.0301003 MHz
NUC1                 13C
P1                 12.00 usec
P13              2000.00 usec
PLW0                   0 W
PLW1         61.49700165 W
SPNAM[5]     Crp80comp.4
SPOAL5             0.500
SPOFFS5                0 Hz
SPW5         18.04100037 W
SFO2         700.0028000 MHz
NUC2                  1H
CPDPRG[2         waltz16
P3                 16.00 usec
P4                 32.00 usec
PCPD2              65.00 usec
PLW2         11.27900028 W
PLW12         0.68339998 W

F2 - Processing parameters
SI                 65536
SF           176.0151419 MHz
WDW                   EM
SSB                    0
LB                  2.00 Hz
GB                     0
PC                  1.50
```

**Figure S11. HSQC NMR spectrum (700 MHz, CDCl3) of 2**

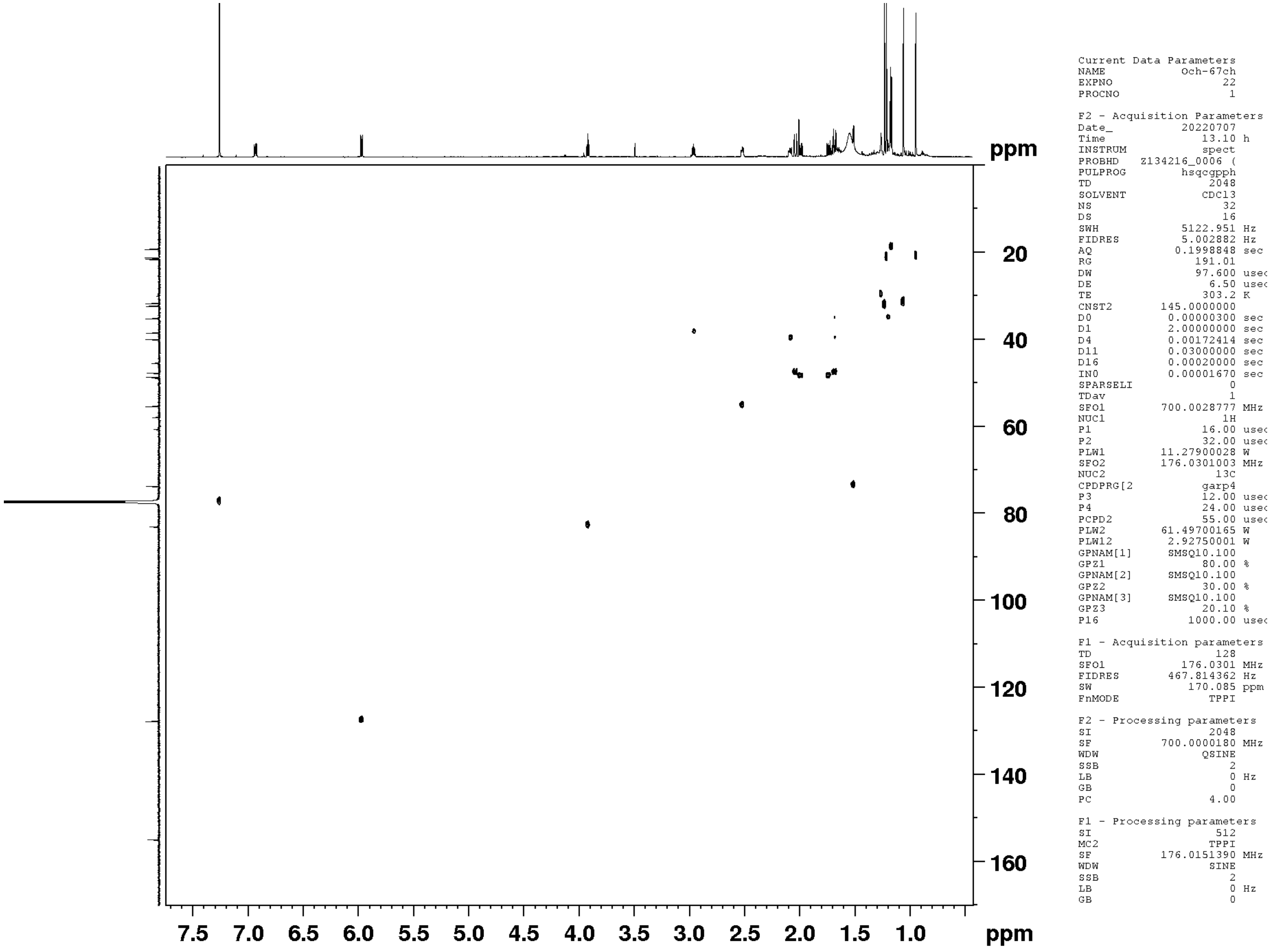

**Figure S12. HMBC NMR spectrum (700 MHz, CDCl3) of 2**

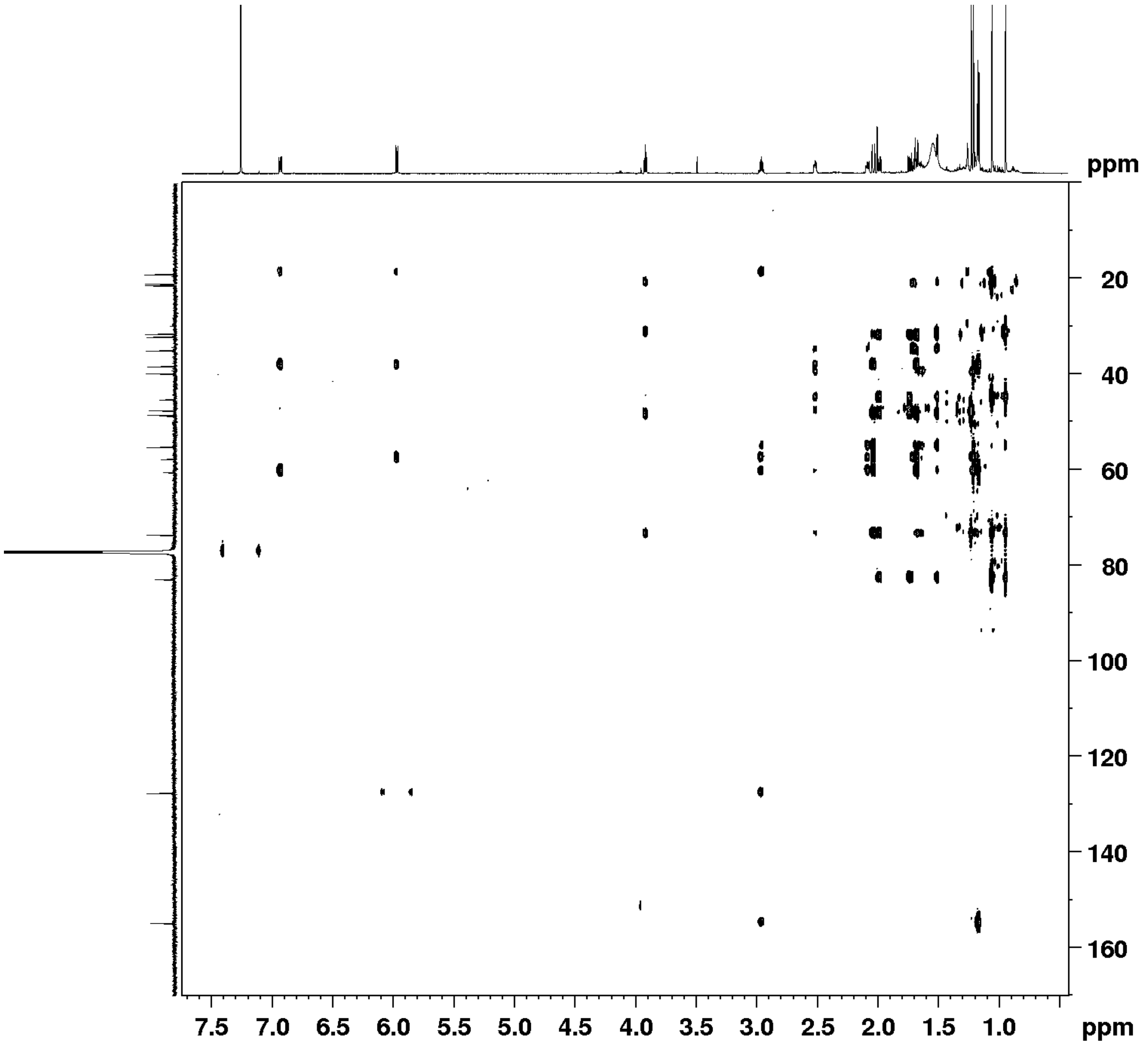


```
Current Data Parameters
NAME            Och-67ch
EXPNO                 24
PROCNO                 1

F2 - Acquisition Parameters
Date_           20220707
Time               21.26 h
INSTRUM            spect
PROBHD   Z134216_0006 (
PULPROG    hmbcgplpndqf
TD                  4096
SOLVENT            CDCl3
NS                    80
DS                    16
SWH             5122.951 Hz
FIDRES          2.501441 Hz
AQ             0.3997696 sec
RG                191.01
DW                97.600 usec
DE                  6.50 usec
TE                 303.0 K
CNST2        145.0000000
CNST13         5.0000000
D0            0.00000300 sec
D1            2.00000000 sec
D2            0.00344828 sec
D6            0.10000000 sec
D16           0.00020000 sec
IN0           0.00001180 sec
SPARSELI               0
TDav                   1
SFO1         700.0028777 MHz
NUC1                  1H
P1                 16.00 usec
P2                 32.00 usec
PLW1         11.27900028 W
SFO2         176.0362608 MHz
NUC2                 13C
P3                 12.00 usec
PLW2         61.49700165 W
GPNAM[1]      SMSQ10.100
GPZ1               50.00 %
GPNAM[2]      SMSQ10.100
GPZ2               30.00 %
GPNAM[3]      SMSQ10.100
GPZ3               40.10 %
P16              1000.00 usec

F1 - Acquisition parameters
TD                   128
SFO1            176.0363 MHz
FIDRES        662.076294 Hz
SW               240.705 ppm
FnMODE                QF

F2 - Processing parameters
SI                  2048
SF           700.0000180 MHz
WDW                 SINE
SSB                    3
LB                     0 Hz
GB                     0
PC                  4.00

F1 - Processing parameters
SI                   512
MC2                   QF
SF           176.0151390 MHz
WDW                 SINE
SSB                    0
LB                     0 Hz
GB                     0
```

**Figure S13. ¹H-¹H COSY NMR spectrum (700 MHz, CDCl3) of 2**

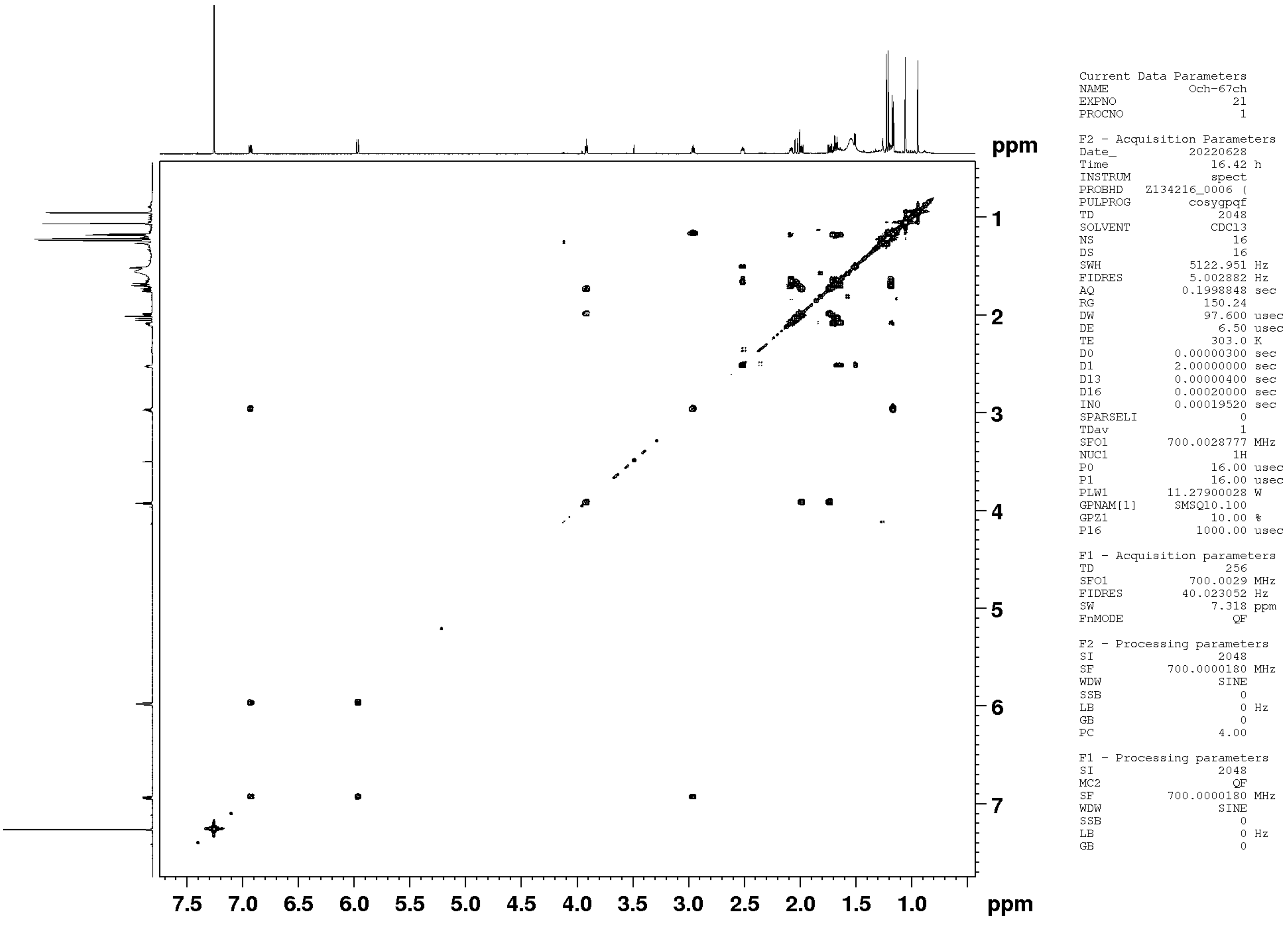

**Figure S14. ROESY NMR spectrum (700 MHz, CDCl3) of 2**

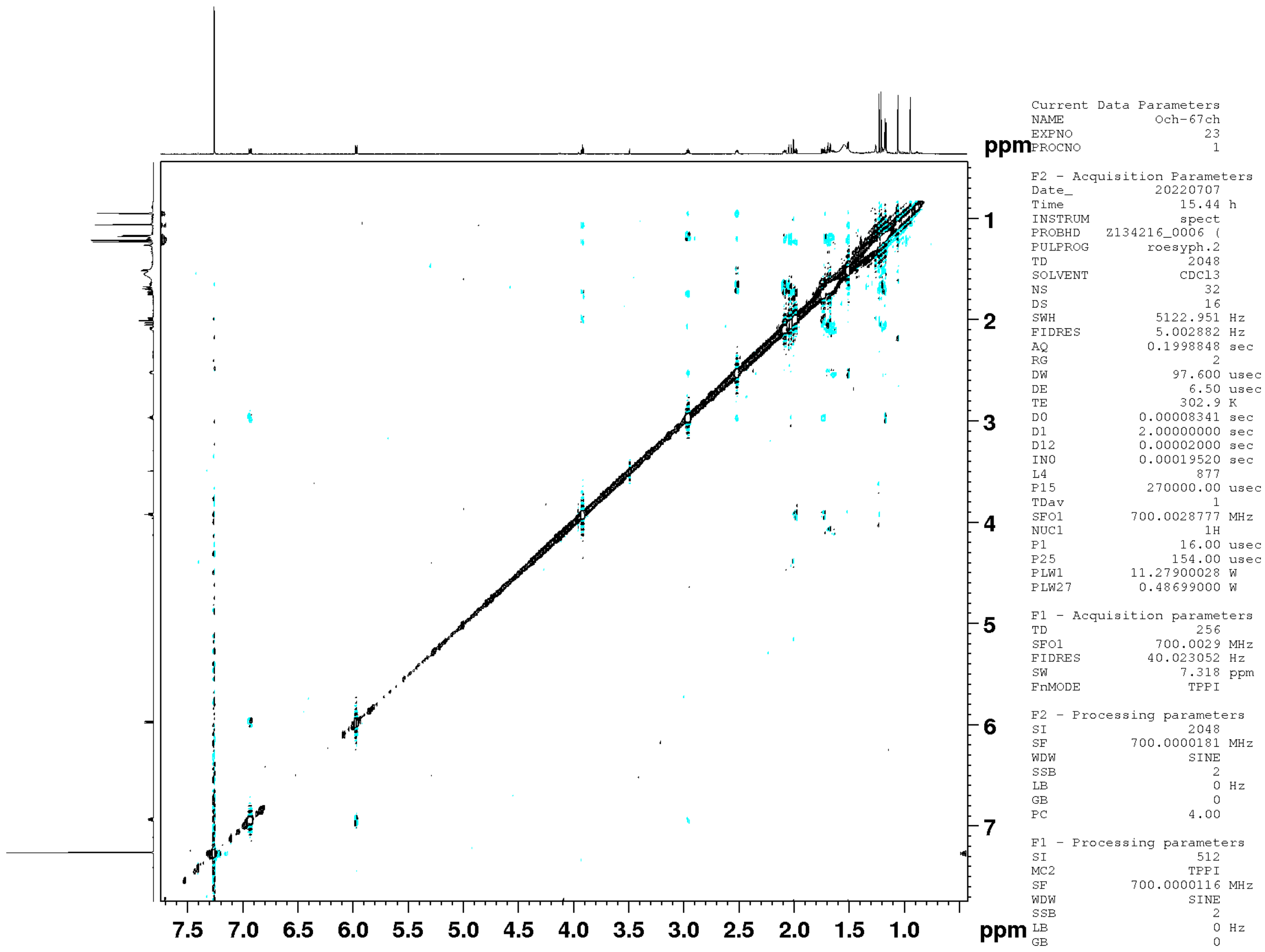

**Figure S15. ¹H NMR spectrum (700 MHz, CDCl3) of 3**

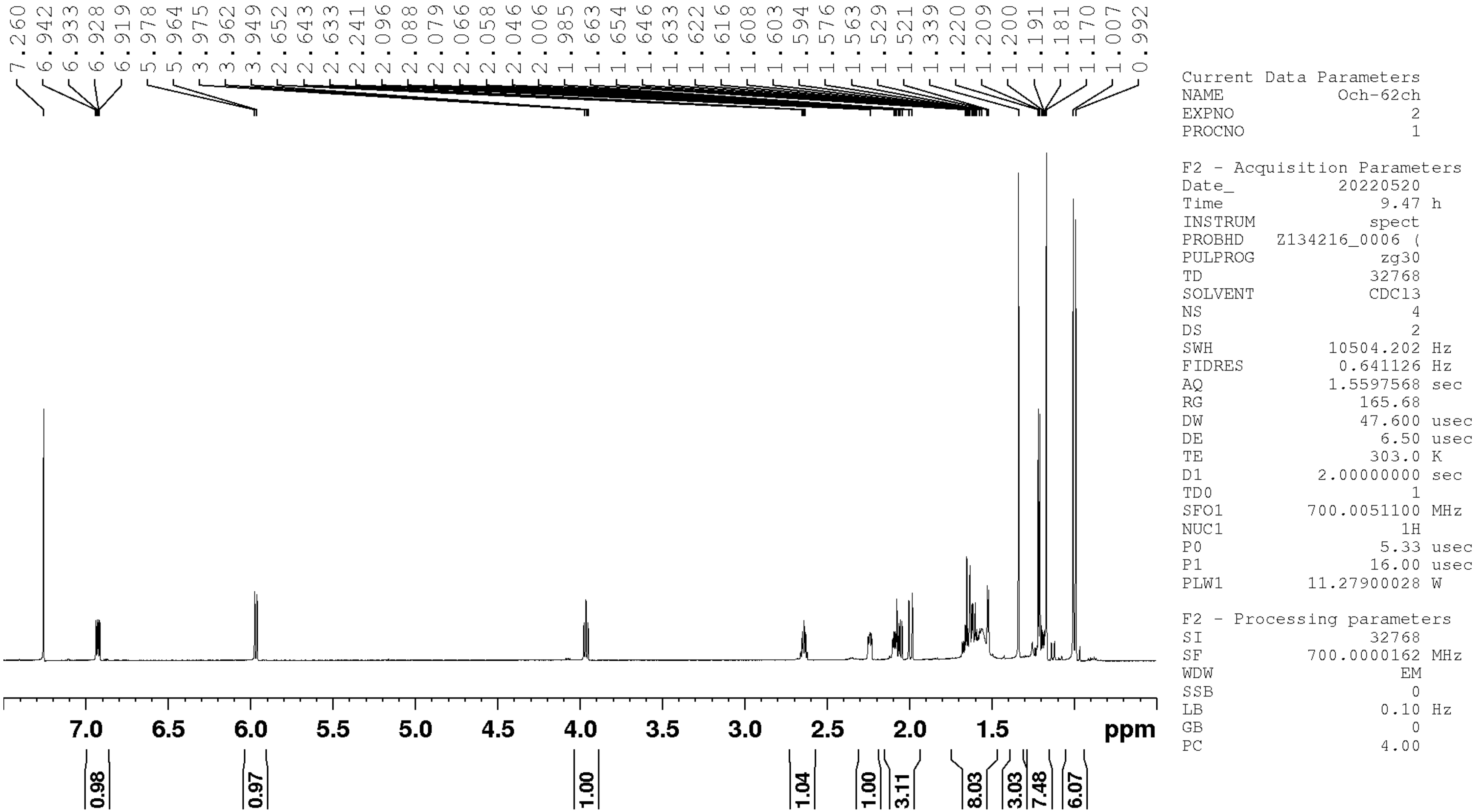

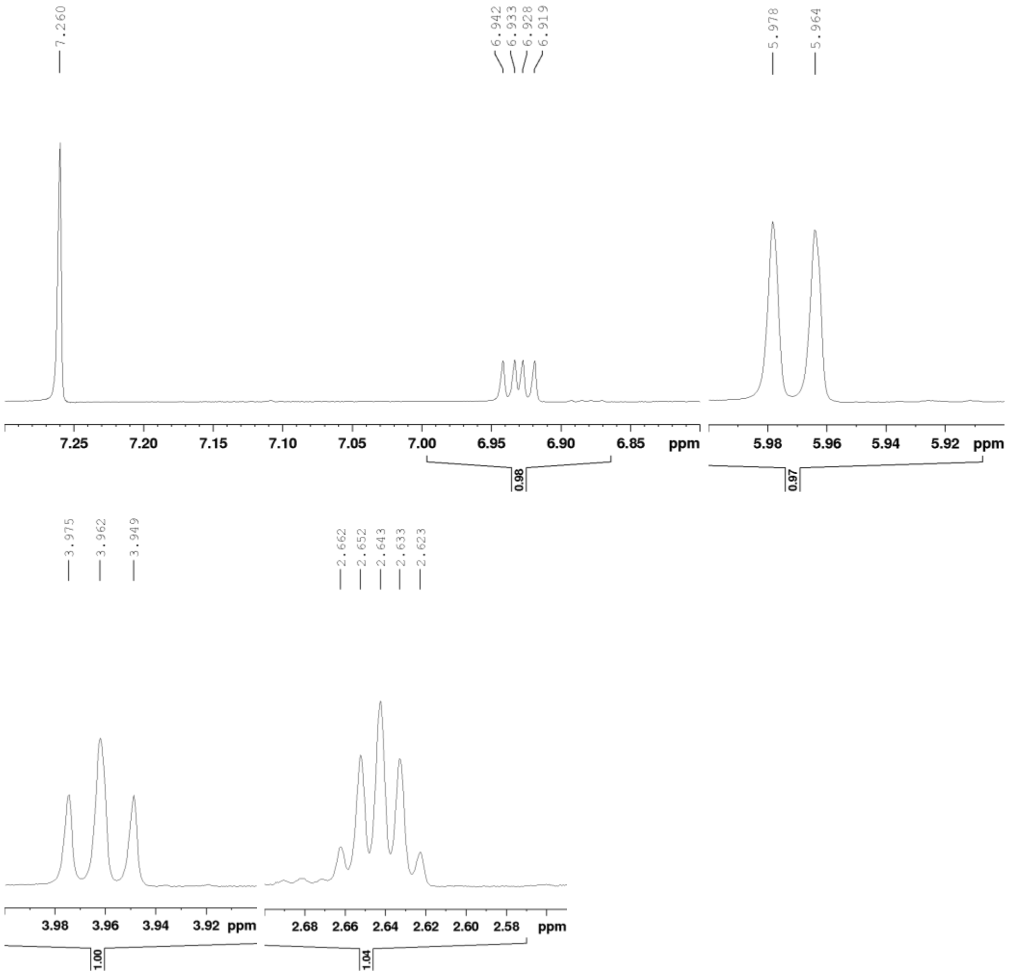
7.260
6.942
6.933
6.928
6.919
7.25 7.20 7.15 7.10 7.05 7.00 6.95 6.90 6.85 ppm
0.98
5.978
5.964
5.98 5.96 5.94 5.92 ppm
0.97
3.975
3.962
3.949
3.98 3.96 3.94 3.92 ppm
1.00
2.662
2.652
2.643
2.633
2.623
2.68 2.66 2.64 2.62 2.60 2.58 ppm
1.04

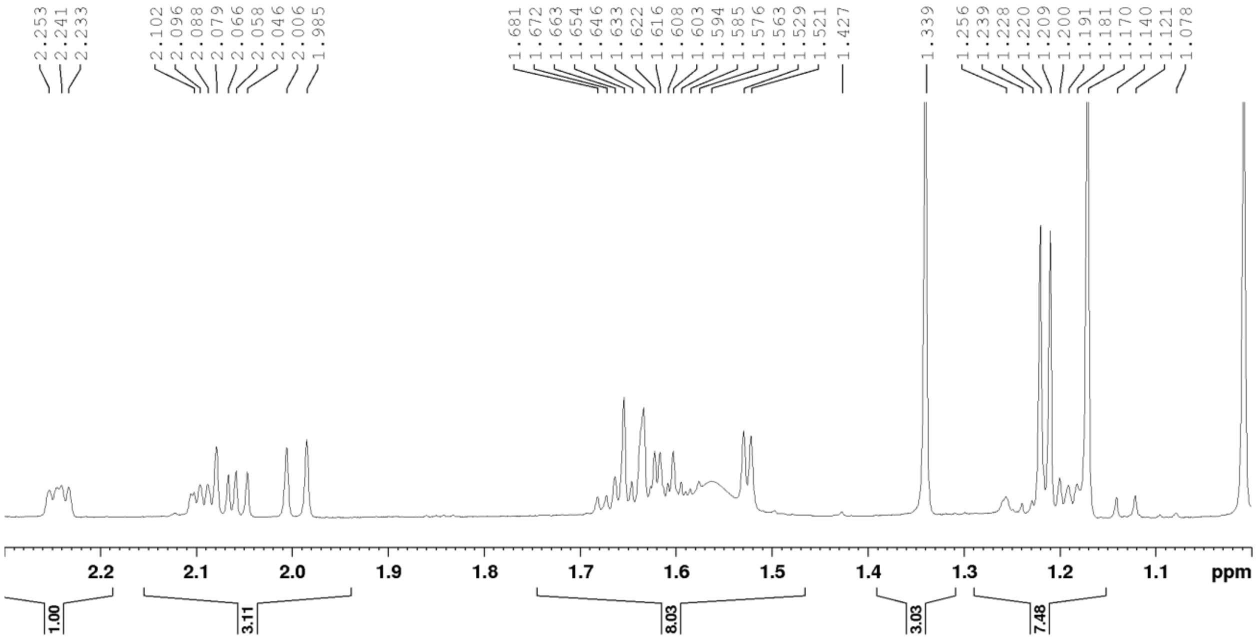
2.253
2.241
2.233
2.102
2.096
2.088
2.079
2.066
2.058
2.046
2.006
1.985
1.681
1.672
1.663
1.654
1.646
1.633
1.622
1.616
1.608
1.603
1.594
1.585
1.576
1.563
1.529
1.521
1.427
1.339
1.256
1.239
1.228
1.220
1.209
1.200
1.191
1.181
1.170
1.140
1.121
1.078
2.2
2.1
2.0
1.9
1.8
1.7
1.6
1.5
1.4
1.3
1.2
1.1
ppm
1.00
3.11
8.03
3.03
7.48

**Figure S16. $^{13}$C NMR spectrum (700 MHz, CDCl3) of 3**

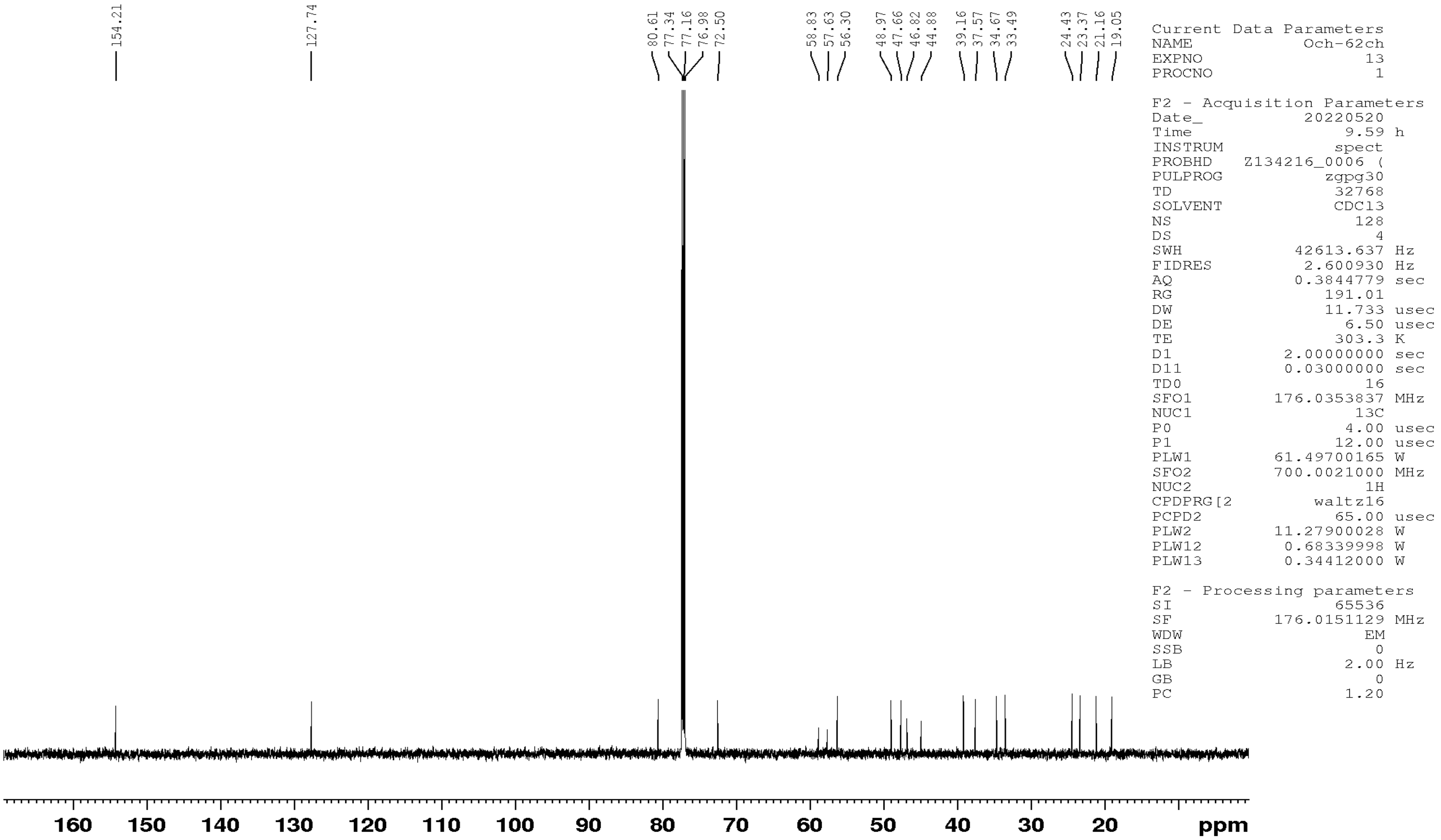

**Figure S17.** $^{1}$H NMR spectrum (700 MHz, CDCl3) of 4

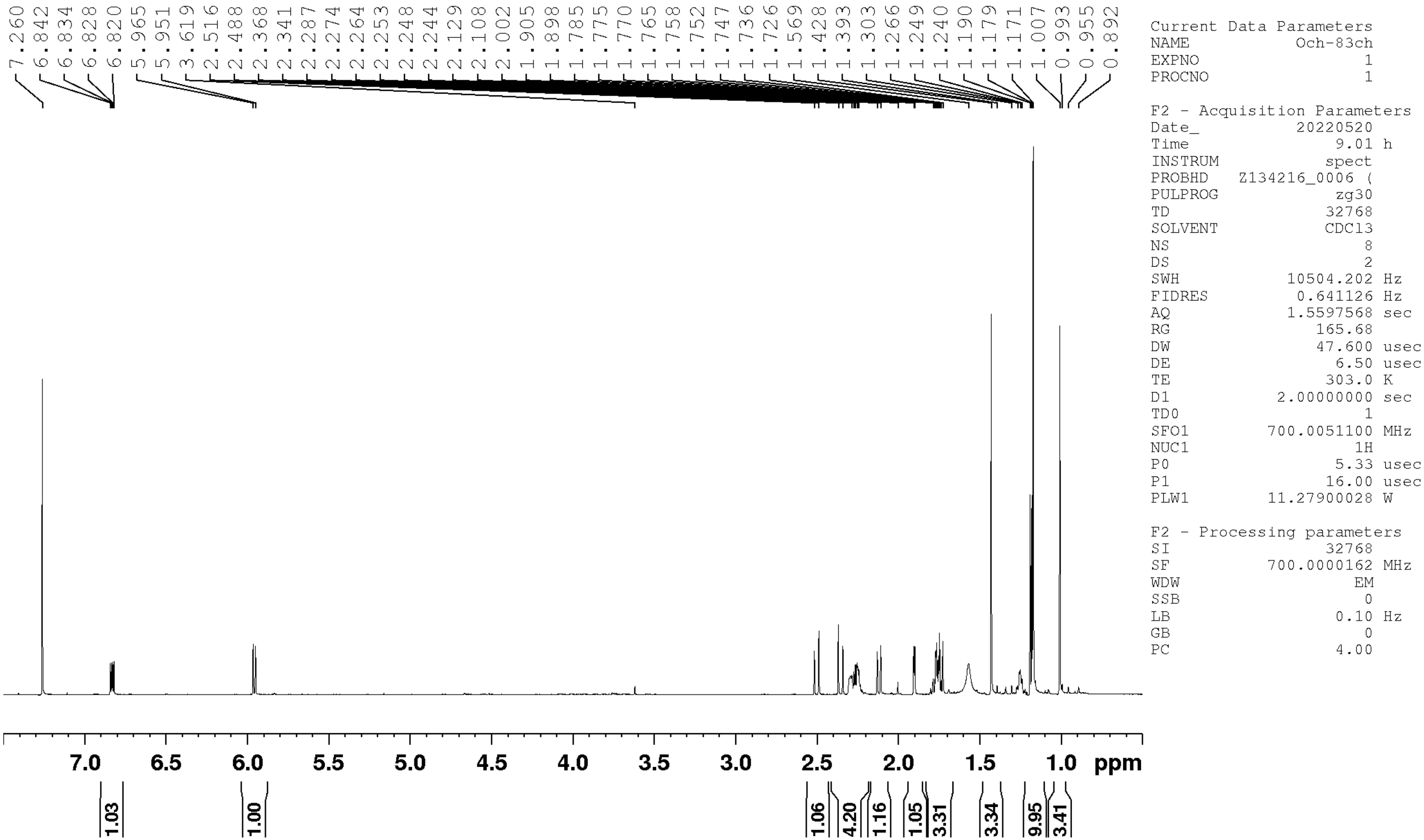

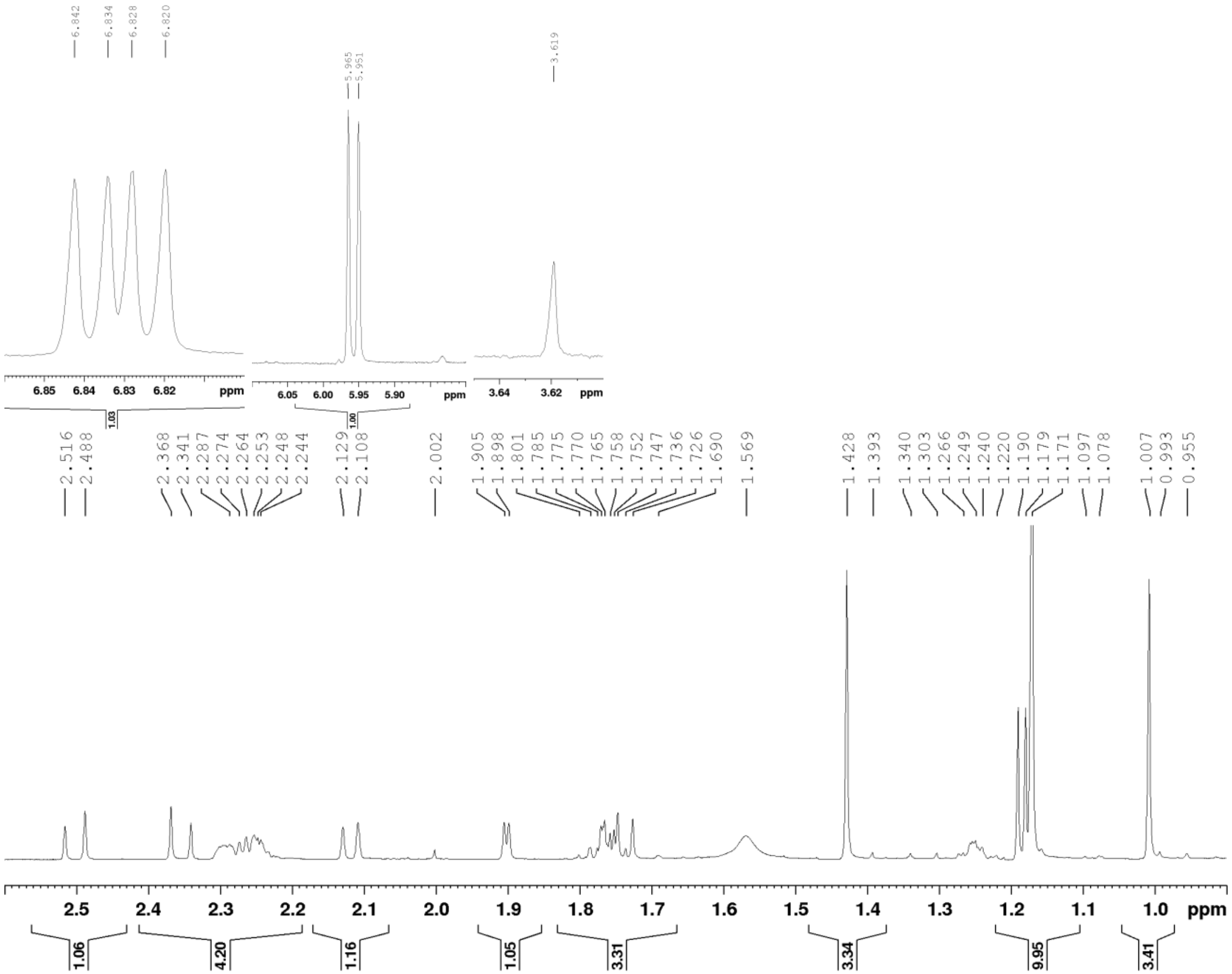

6.842
6.834
6.828
6.820
5.965
5.951
3.619
2.516
2.488
2.368
2.341
2.287
2.274
2.264
2.253
2.248
2.244
2.129
2.108
2.002
1.905
1.898
1.801
1.785
1.775
1.770
1.765
1.758
1.752
1.747
1.736
1.726
1.690
1.569
1.428
1.393
1.340
1.303
1.266
1.249
1.240
1.220
1.190
1.179
1.171
1.097
1.078
1.007
0.993
0.955
ppm
1.03
1.00
1.06
4.20
1.16
1.05
3.31
3.34
9.95
3.41

**Figure S18. $^{13}$C NMR spectrum (700 MHz, CDCl3) of 4**

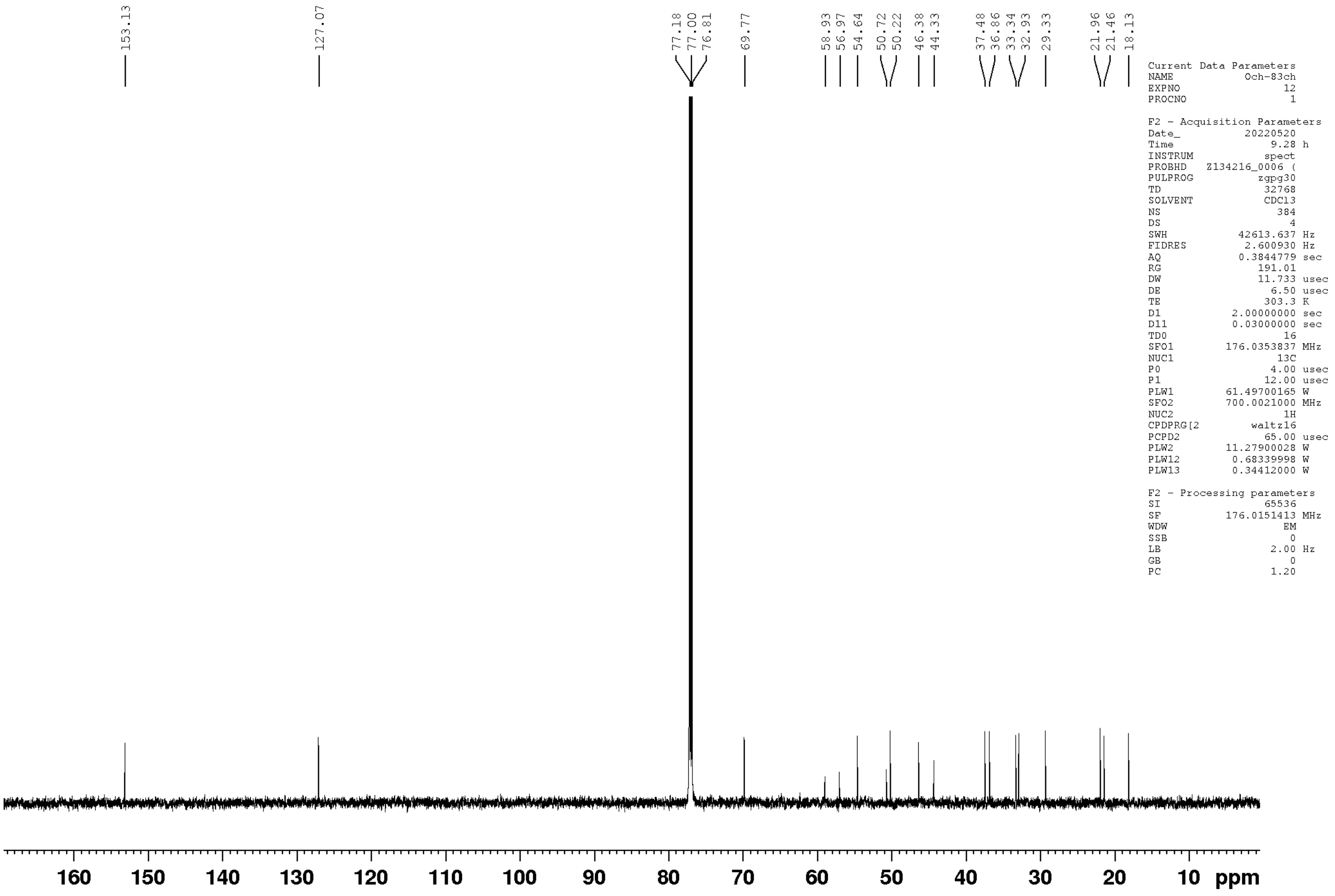

**Figure S19. $^1H$ NMR spectrum (500 MHz, $CDCl_3$) of 5**

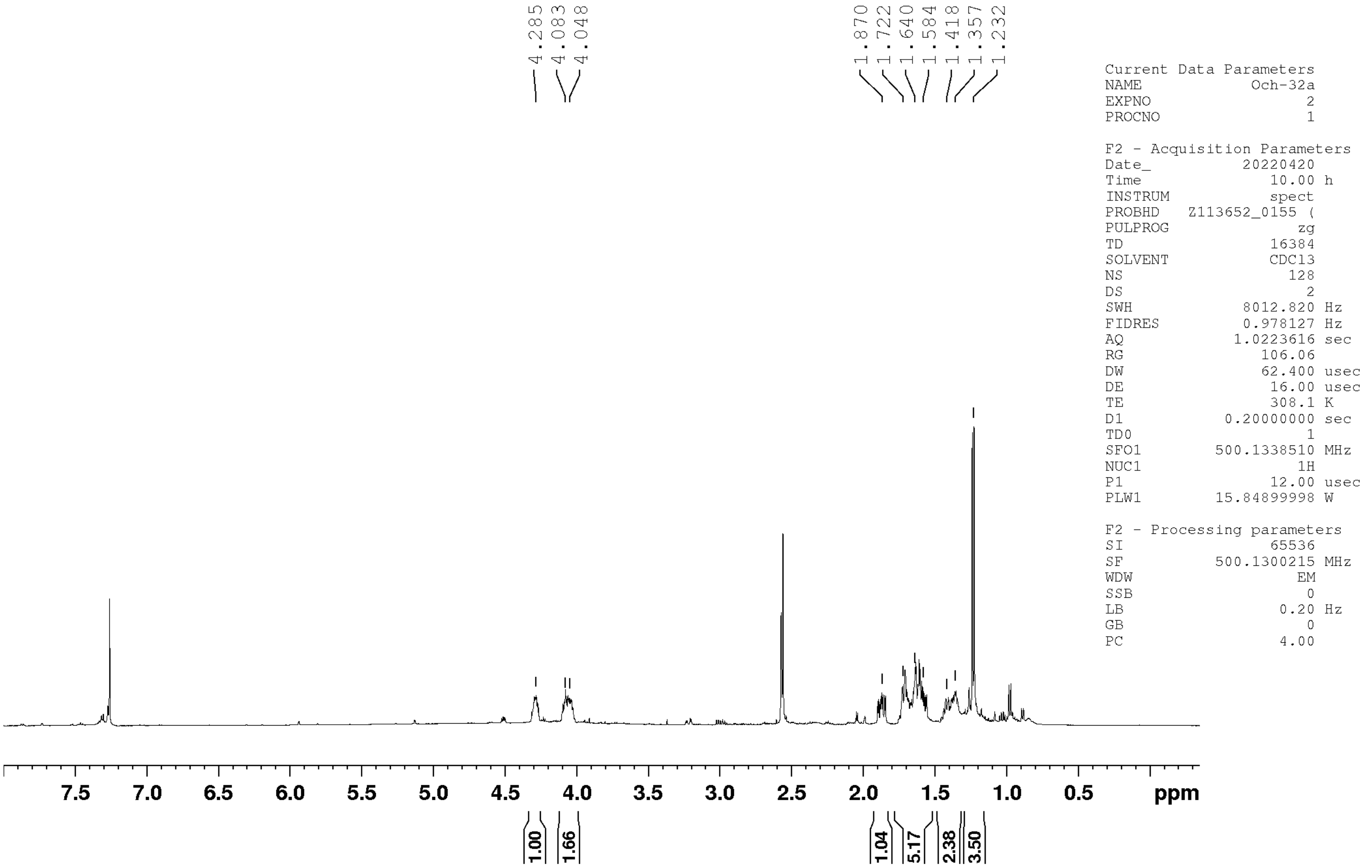

**Figure S20. $^{13}C$ NMR spectrum (500 MHz, $CDCl_3$) of 5**

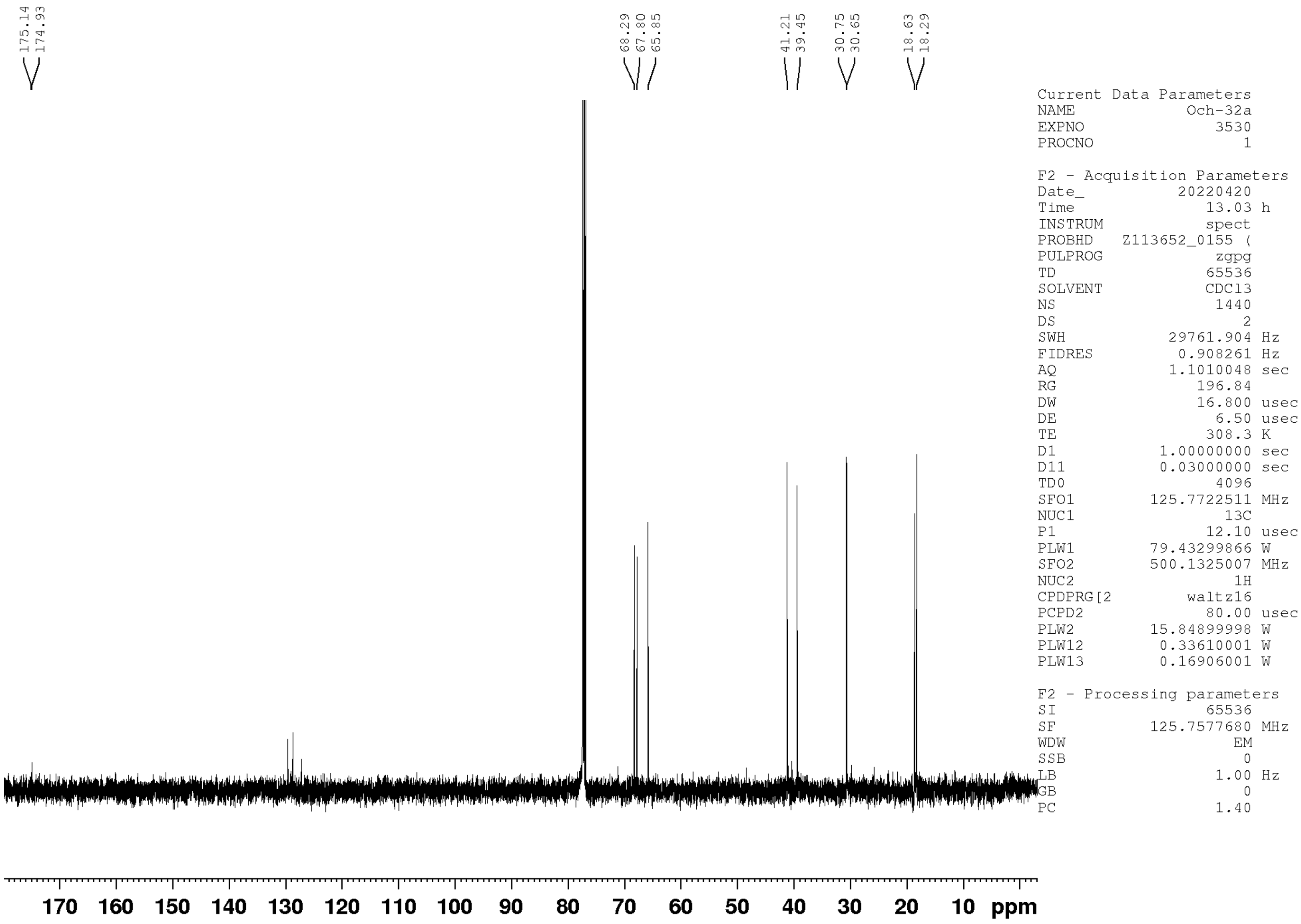


```
Current Data Parameters
NAME             Och-32a
EXPNO               3530
PROCNO                 1

F2 - Acquisition Parameters
Date_           20220420
Time               13.03 h
INSTRUM            spect
PROBHD   Z113652_0155 (
PULPROG             zgpg
TD                 65536
SOLVENT            CDCl3
NS                  1440
DS                     2
SWH            29761.904 Hz
FIDRES          0.908261 Hz
AQ             1.1010048 sec
RG                196.84
DW                16.800 usec
DE                  6.50 usec
TE                 308.3 K
D1            1.00000000 sec
D11           0.03000000 sec
TD0                 4096
SFO1         125.7722511 MHz
NUC1                 13C
P1                 12.10 usec
PLW1         79.43299866 W
SFO2         500.1325007 MHz
NUC2                  1H
CPDPRG[2         waltz16
PCPD2              80.00 usec
PLW2         15.84899998 W
PLW12         0.33610001 W
PLW13         0.16906001 W

F2 - Processing parameters
SI                 65536
SF           125.7577680 MHz
WDW                   EM
SSB                    0
LB                  1.00 Hz
GB                     0
PC                  1.40
```

**Figure S21. DEPT NMR spectrum (500 MHz, $CDCl_3$) of 5**

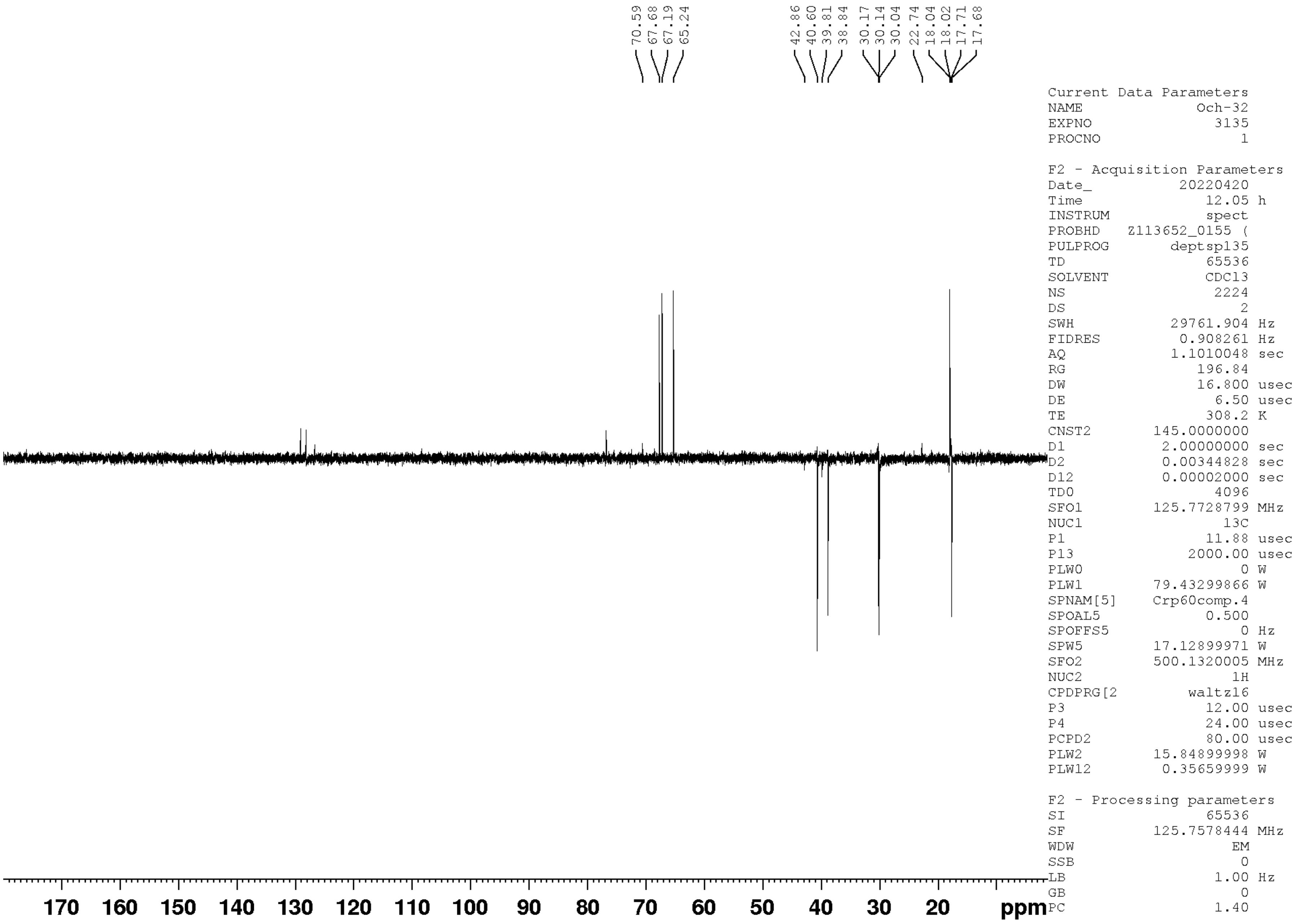


```
Current Data Parameters
NAME              Och-32
EXPNO               3135
PROCNO                 1

F2 - Acquisition Parameters
Date_           20220420
Time               12.05 h
INSTRUM            spect
PROBHD    Z113652_0155 (
PULPROG       deptsp135
TD                 65536
SOLVENT            CDCl3
NS                  2224
DS                     2
SWH            29761.904 Hz
FIDRES          0.908261 Hz
AQ             1.1010048 sec
RG                196.84
DW                16.800 usec
DE                  6.50 usec
TE                 308.2 K
CNST2        145.0000000
D1            2.00000000 sec
D2            0.00344828 sec
D12           0.00002000 sec
TD0                 4096
SFO1         125.7728799 MHz
NUC1                 13C
P1                 11.88 usec
P13              2000.00 usec
PLW0                   0 W
PLW1         79.43299866 W
SPNAM[5]     Crp60comp.4
SPOAL5             0.500
SPOFFS5                0 Hz
SPW5         17.12899971 W
SFO2         500.1320005 MHz
NUC2                  1H
CPDPRG[2         waltz16
P3                 12.00 usec
P4                 24.00 usec
PCPD2              80.00 usec
PLW2         15.84899998 W
PLW12         0.35659999 W

F2 - Processing parameters
SI                 65536
SF           125.7578444 MHz
WDW                   EM
SSB                    0
LB                  1.00 Hz
GB                     0
PC                  1.40
```

**Figure S22. HSQC NMR spectrum (500 MHz, $CDCl_3$) of 5**

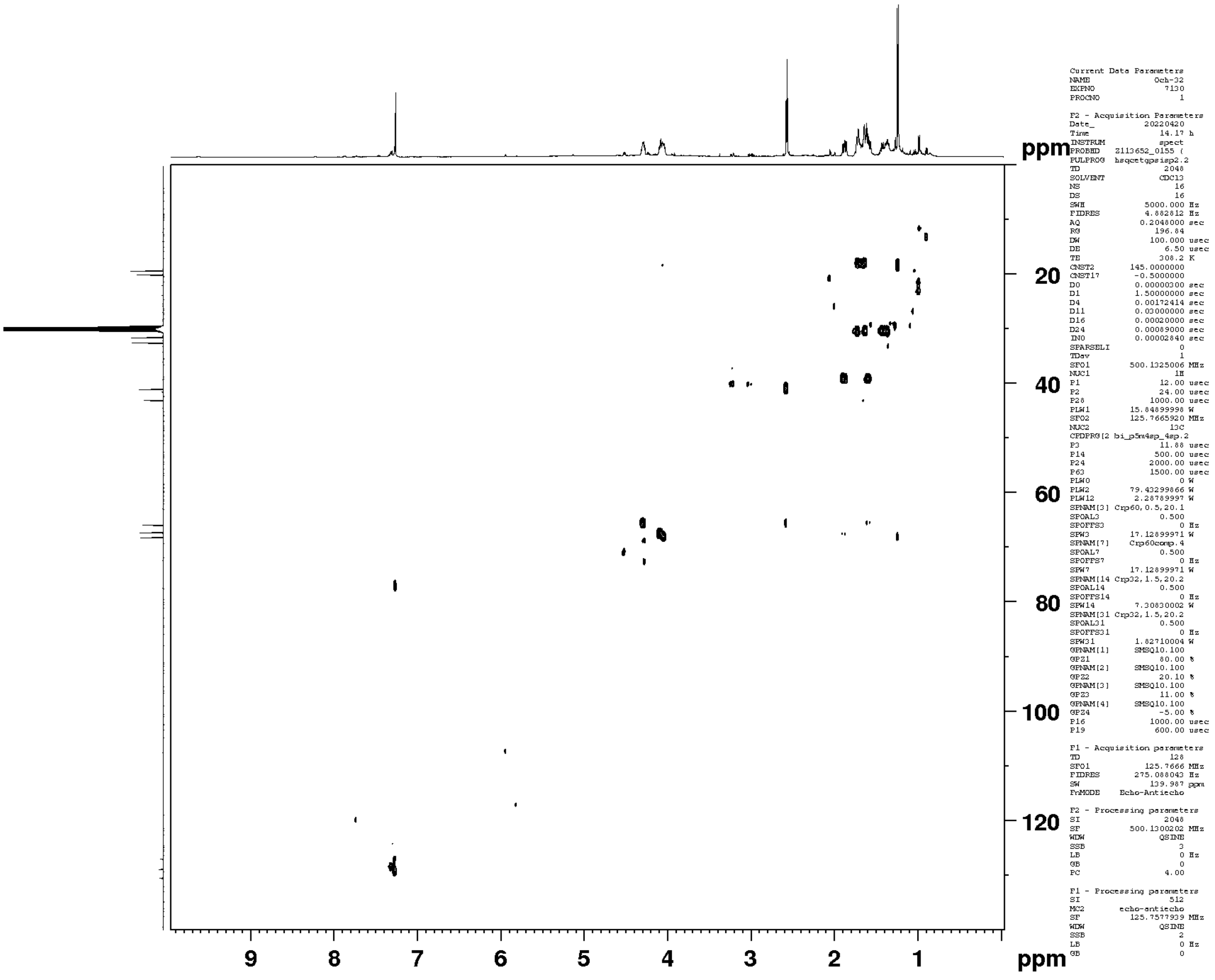

**Figure S23. HMBC NMR spectrum (500 MHz, CDCl₃) of 5**

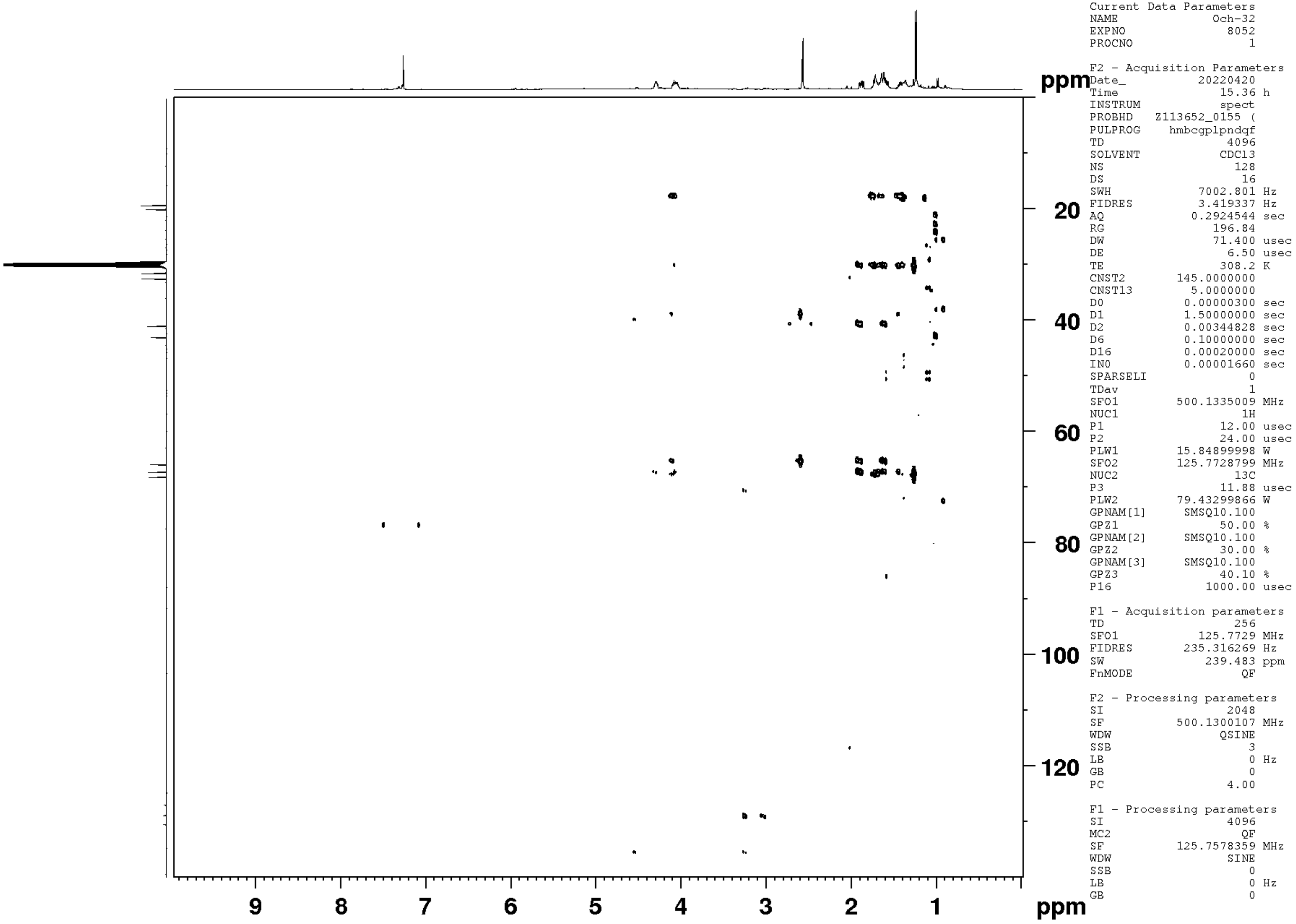

**Figure S24. $^{1}H$-$^{1}H$ COSY NMR spectrum (500 MHz, $CDCl_3$) of 5**

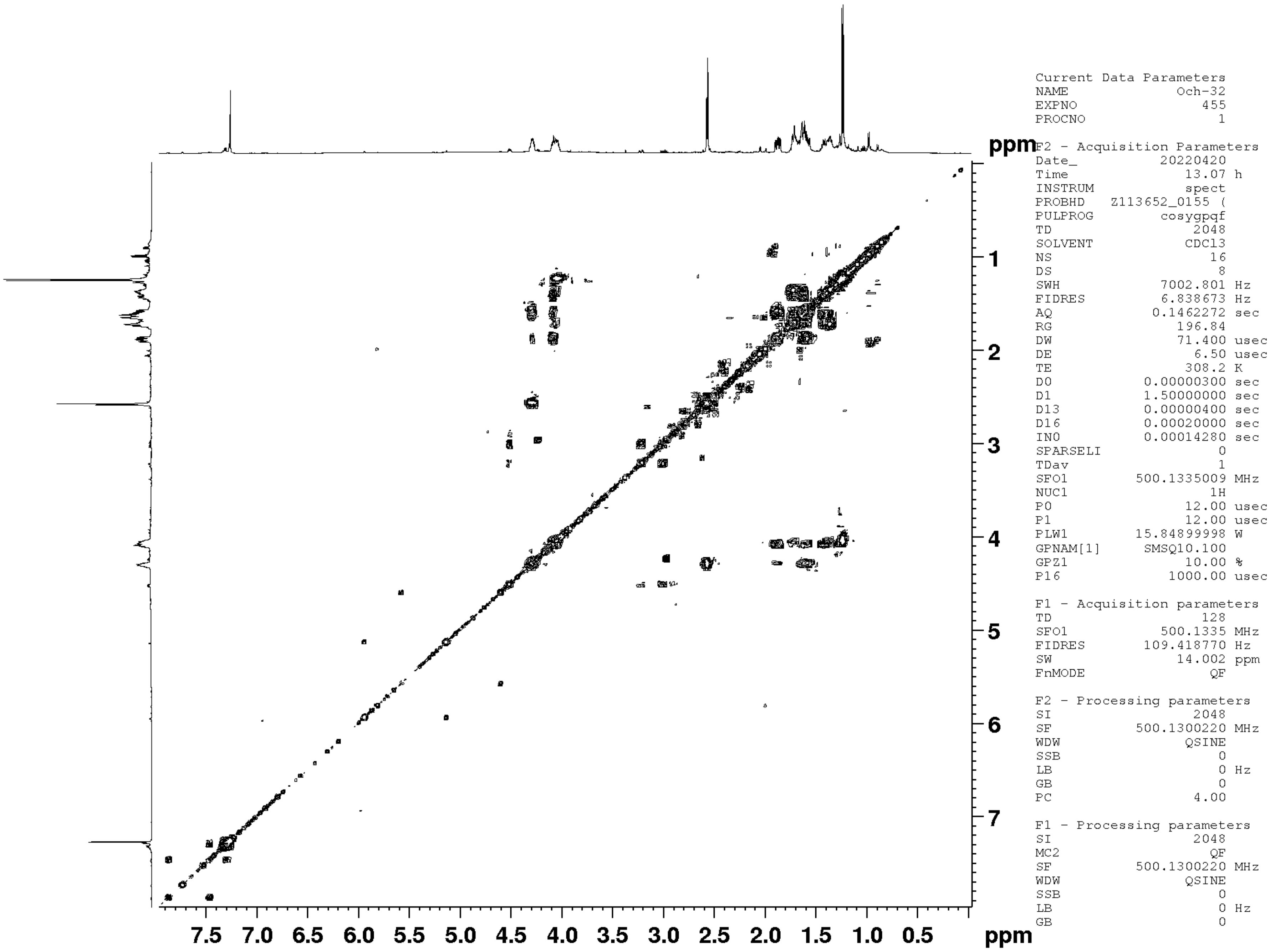

**Figure S25. NOESY NMR spectrum (500 MHz, $CDCl_3$) of 5**

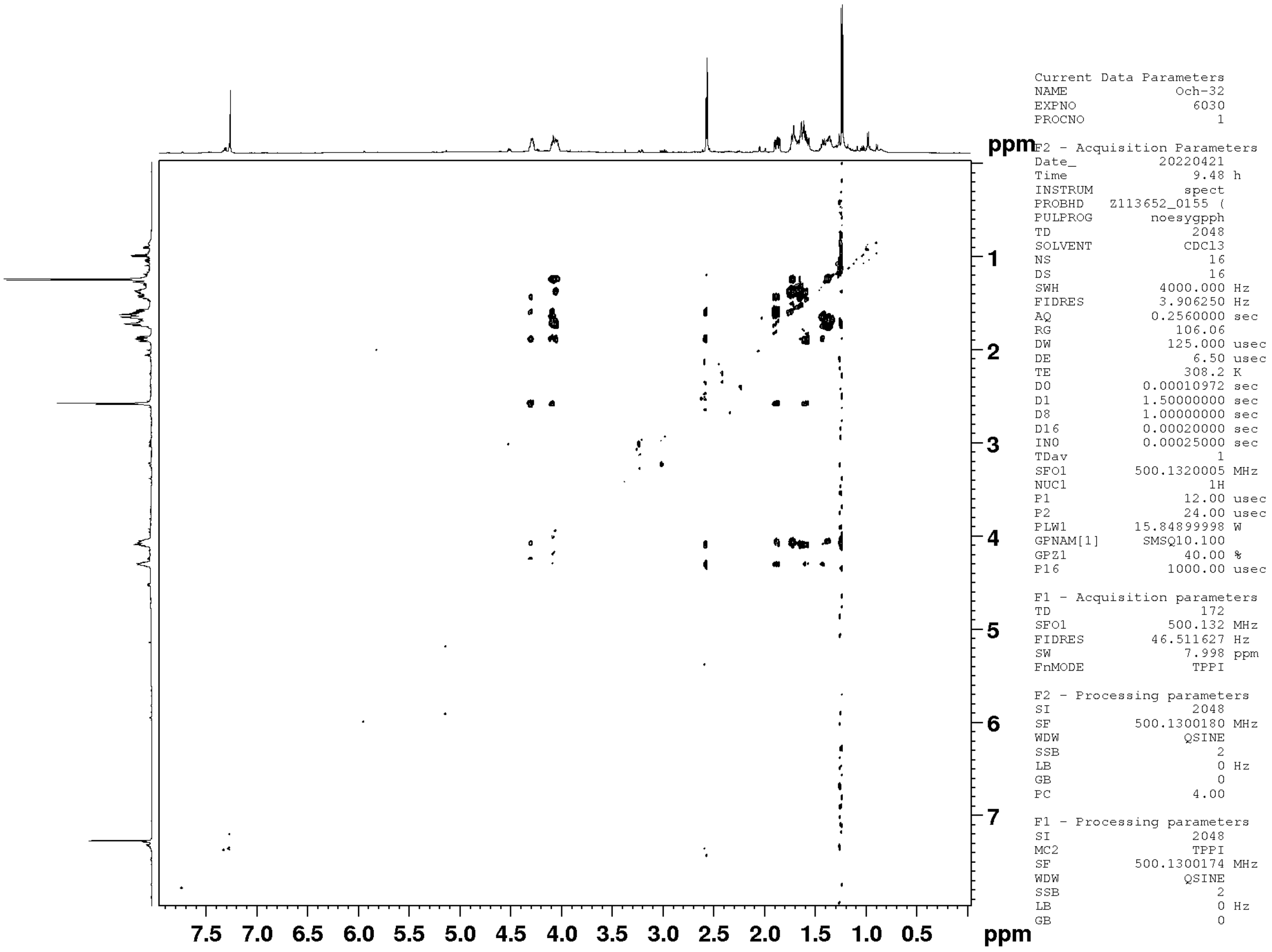

**Figure S26. $^1$H NMR spectrum (500 MHz, acetone-$d_6$) of 6**

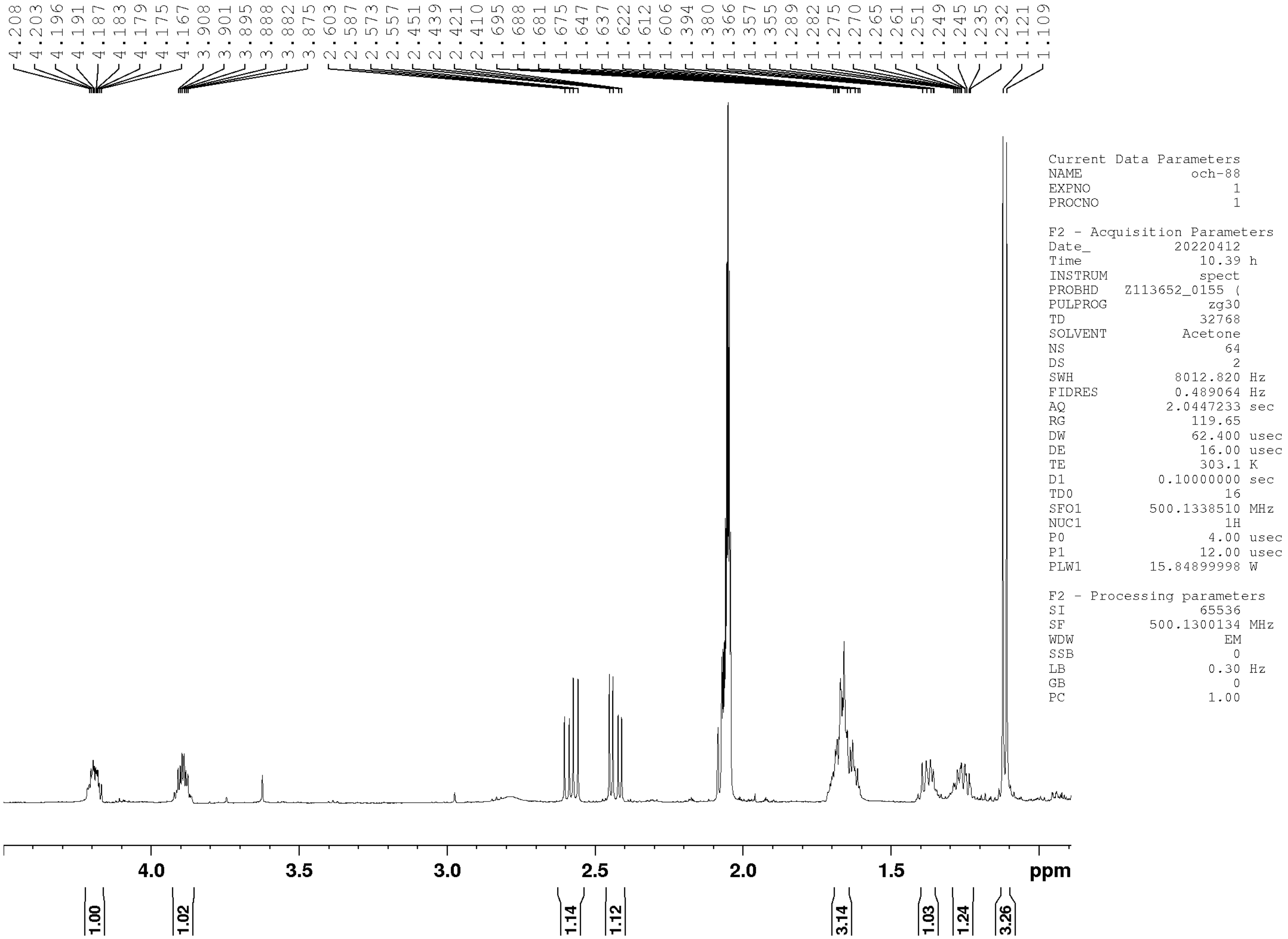

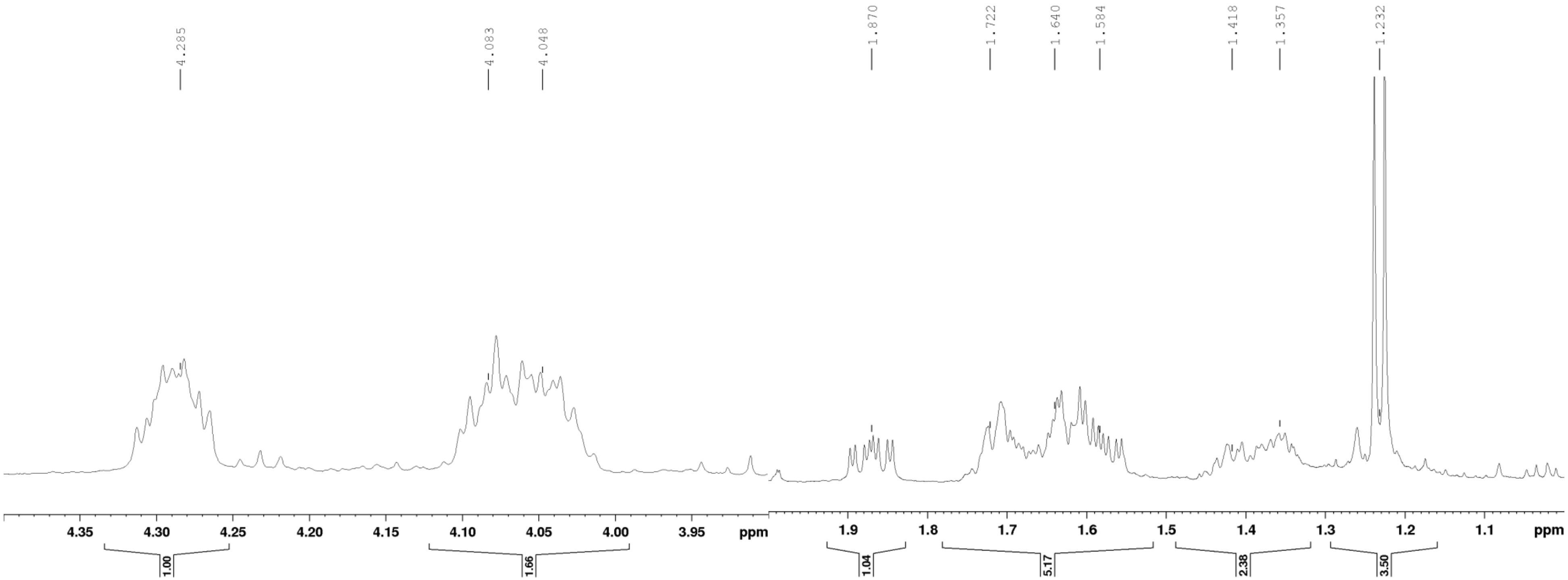
4.285
4.083
4.048
1.870
1.722
1.640
1.584
1.418
1.357
1.232
4.35
4.30
4.25
4.20
4.15
4.10
4.05
4.00
3.95
ppm
1.9
1.8
1.7
1.6
1.5
1.4
1.3
1.2
1.1
ppm
1.00
1.66
1.04
5.17
2.38
3.50

**Figure S27. $^{13}$C NMR spectrum (500 MHz, acetone-$d_6$) of 6**

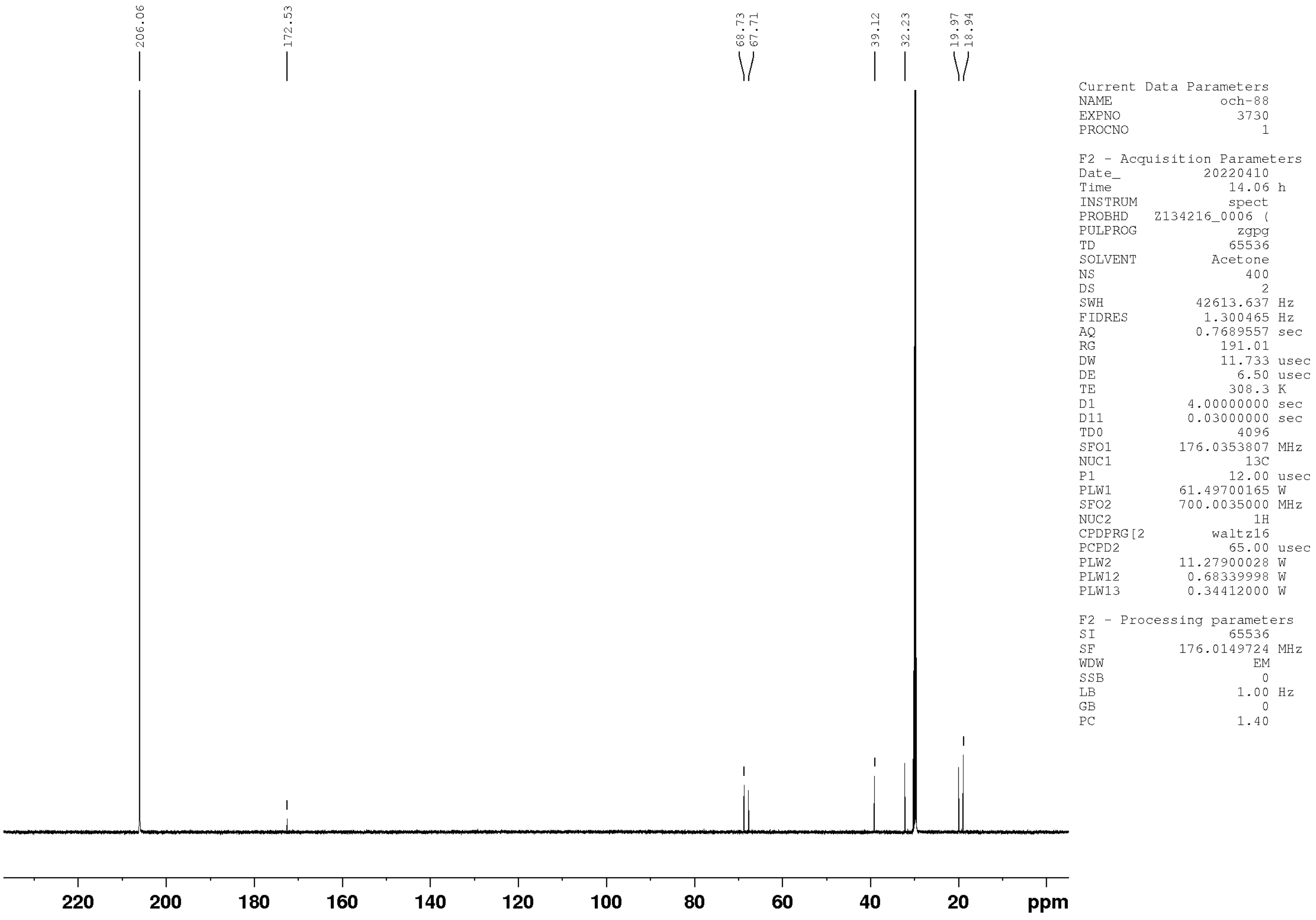

**Figure S28. UV spectrum of 1**

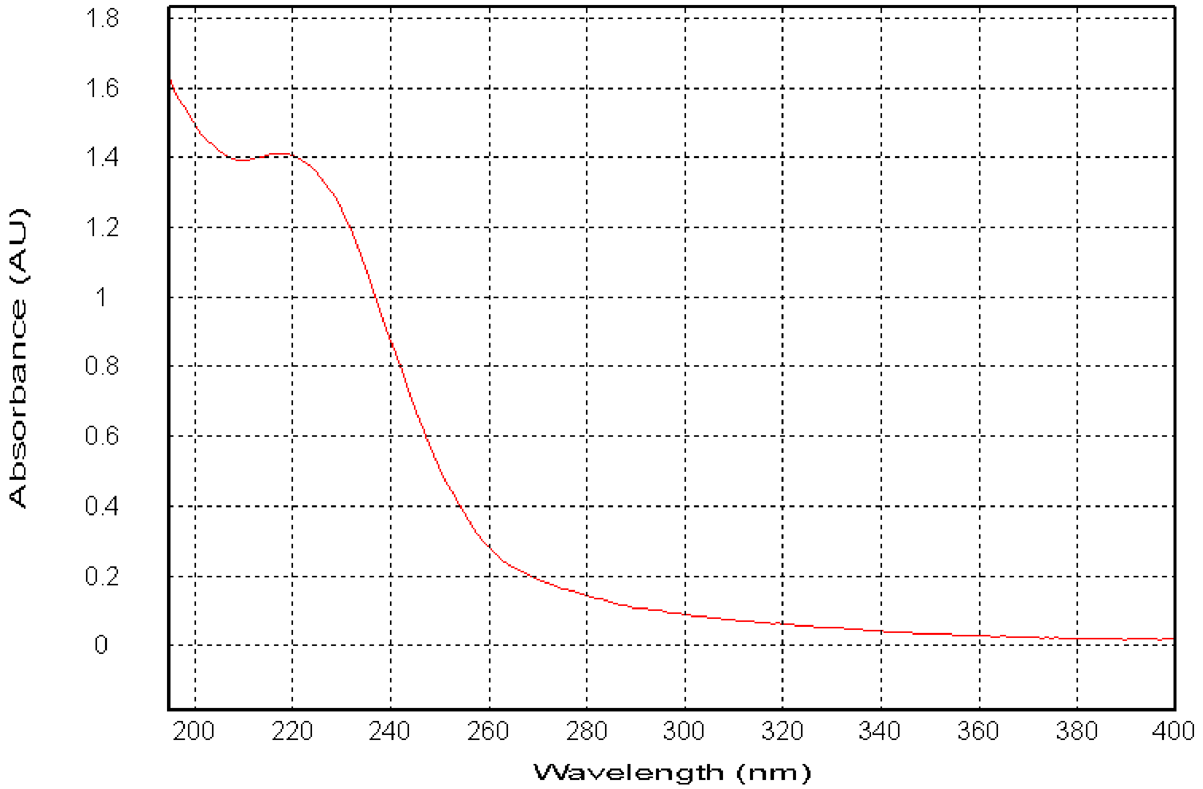


**Figure S29. CD spectrum of 1**

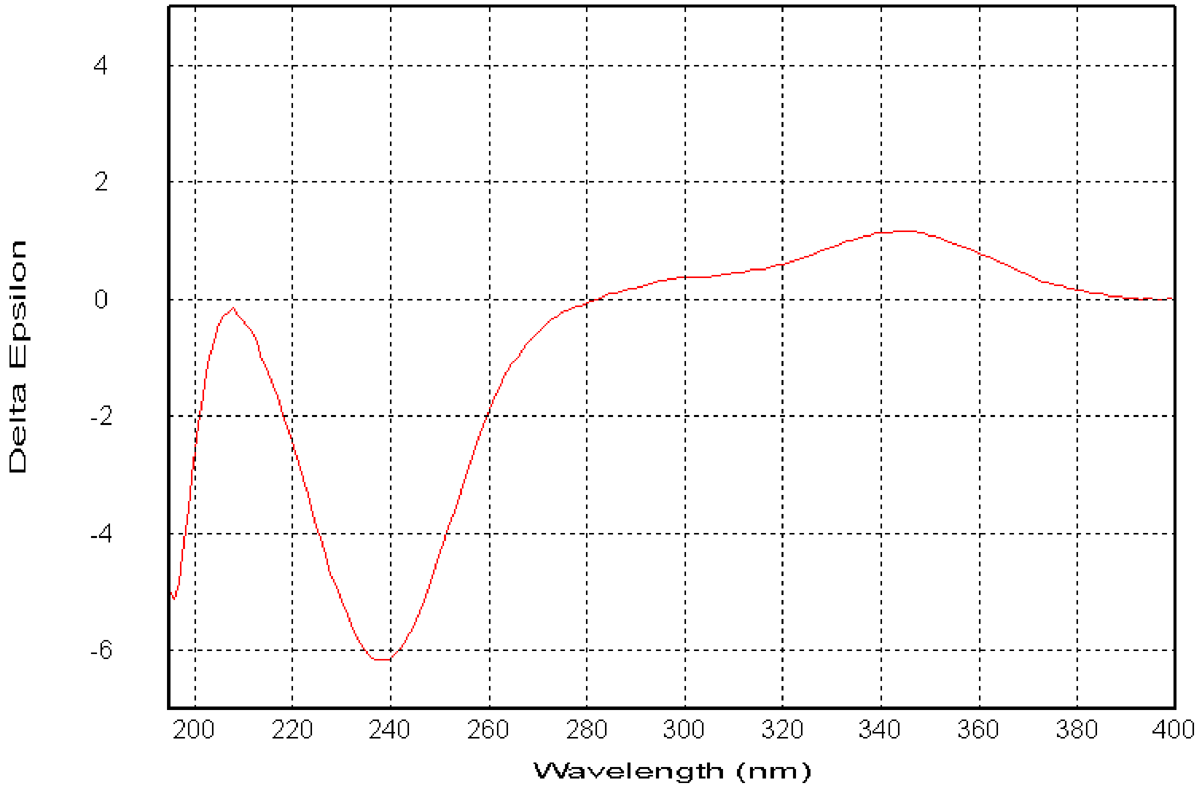

**Figure S30. UV spectrum of 2**

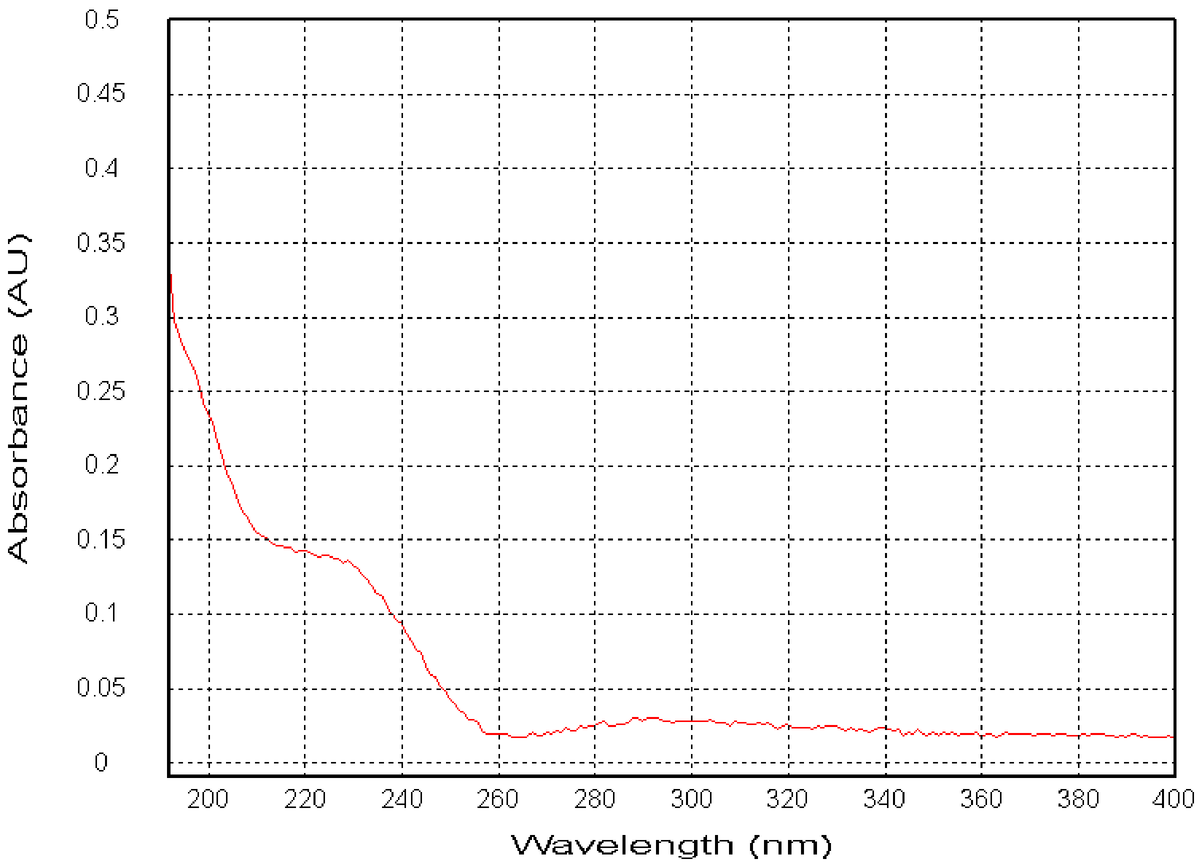


**Figure S31. CD spectrum of 2**

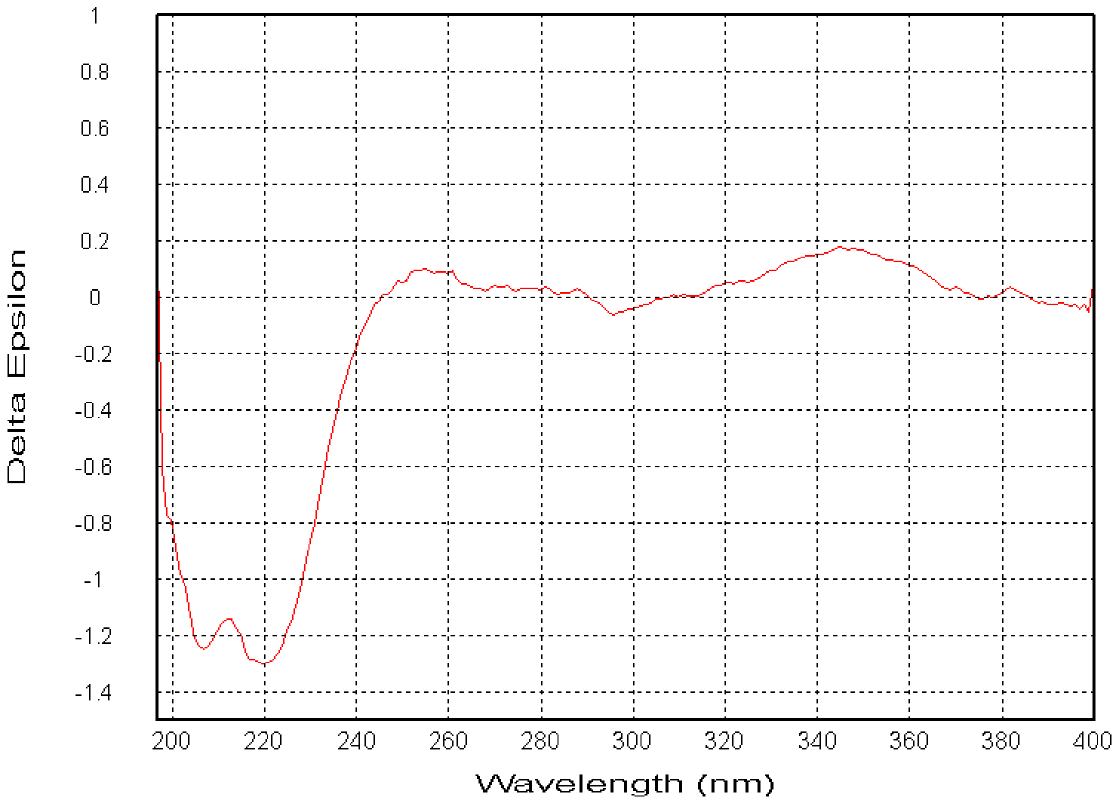

**Figure S32. UV spectrum of 3**

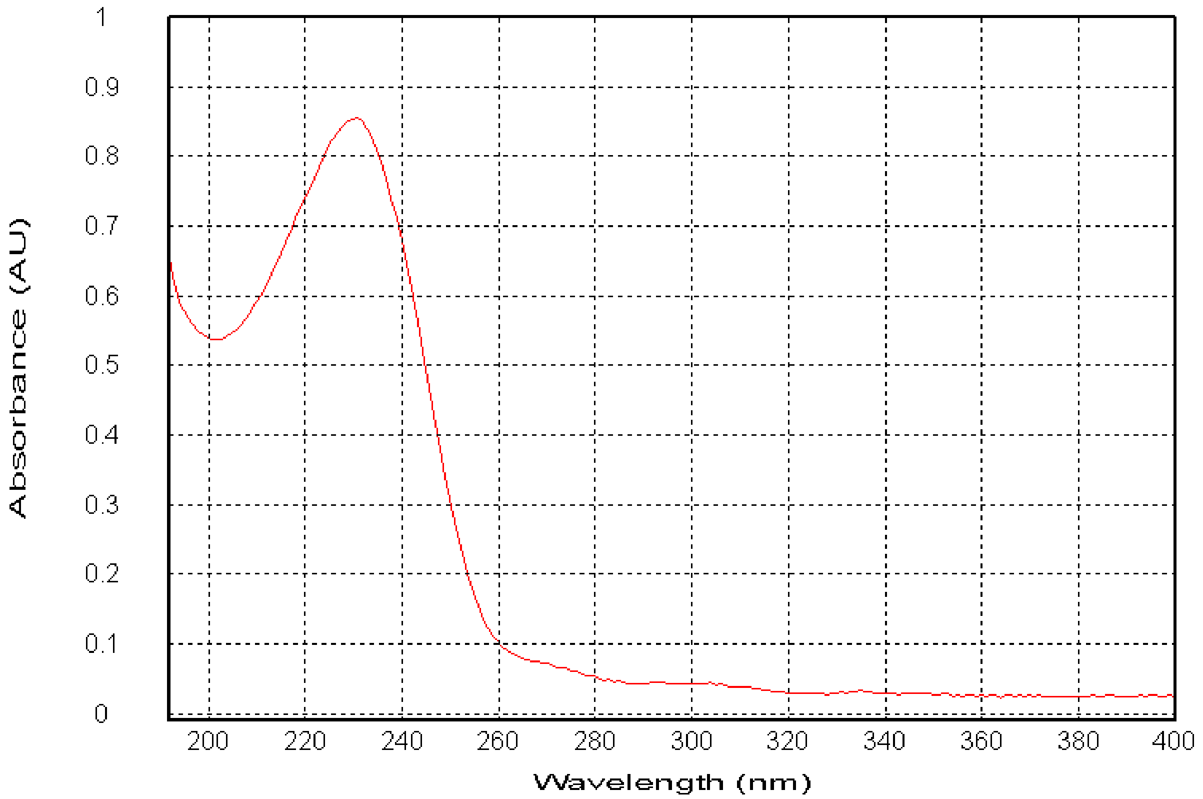


**Figure S33. CD spectrum of 3**

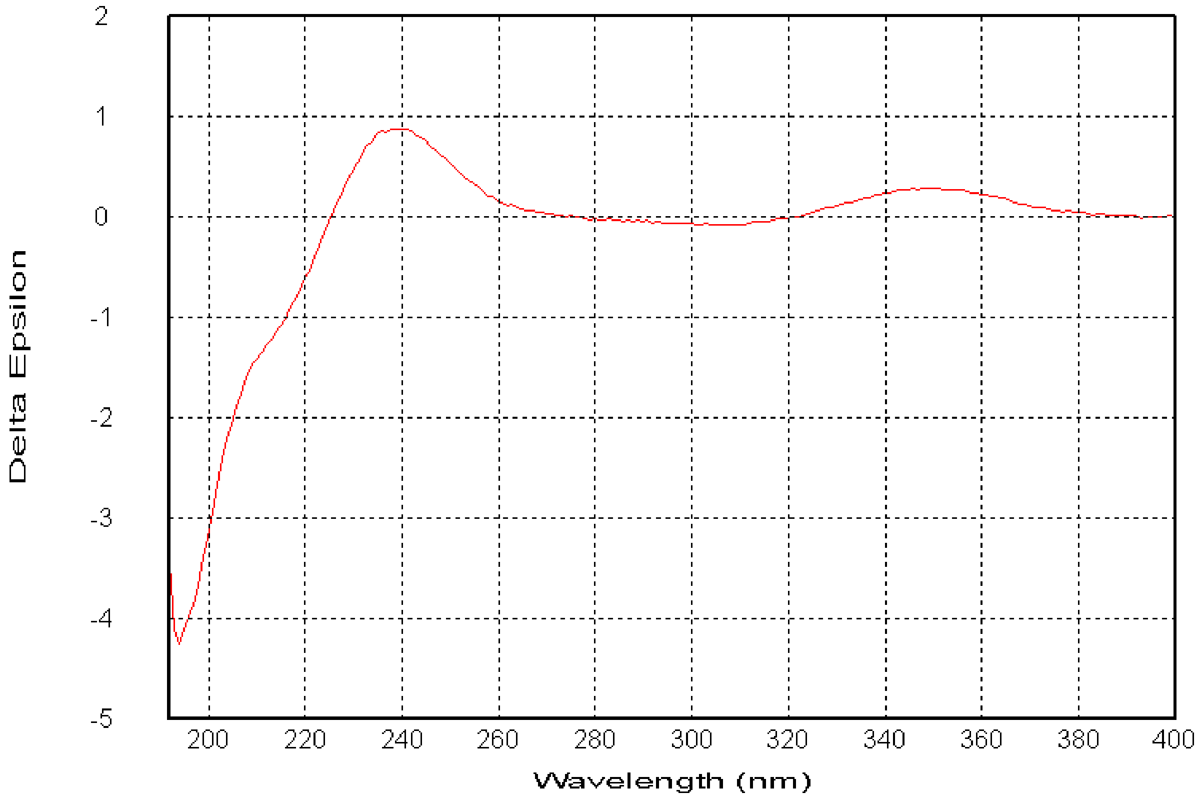

**Figure S34. UV spectrum of 4**

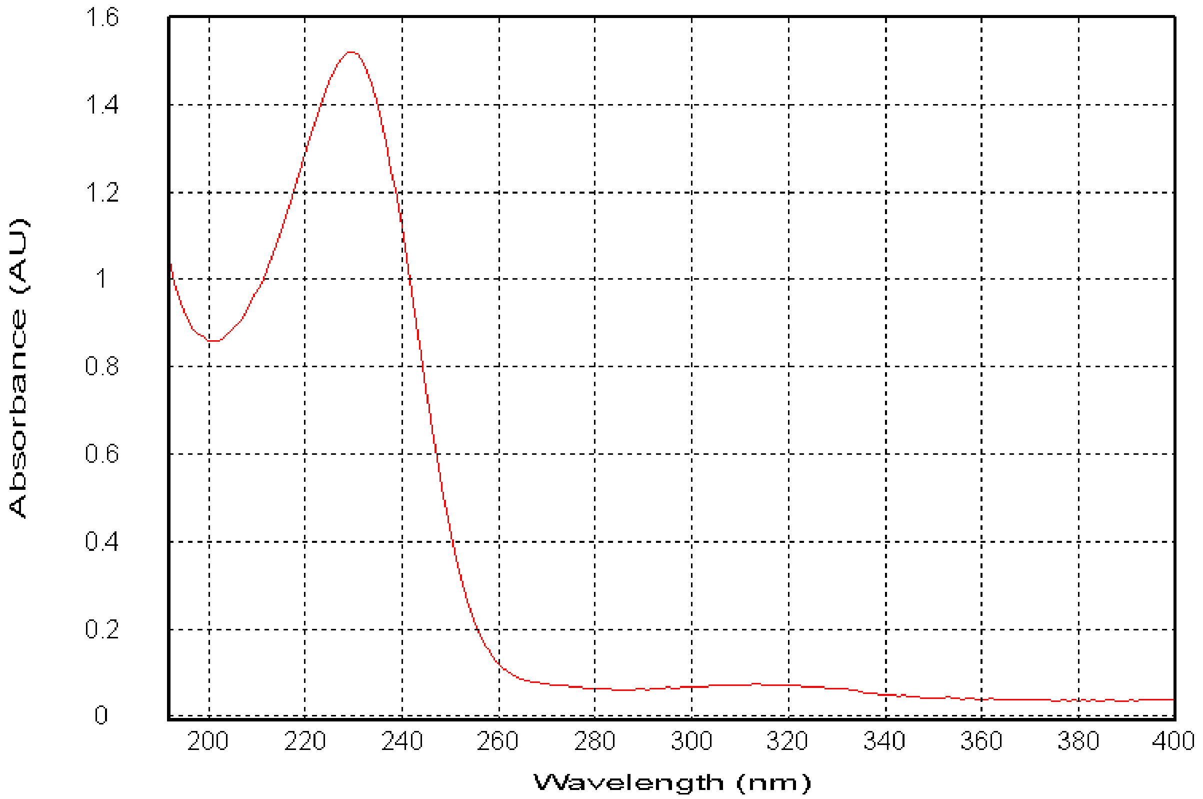


**Figure S35. The experimental and calculated ECD spectra of 4**

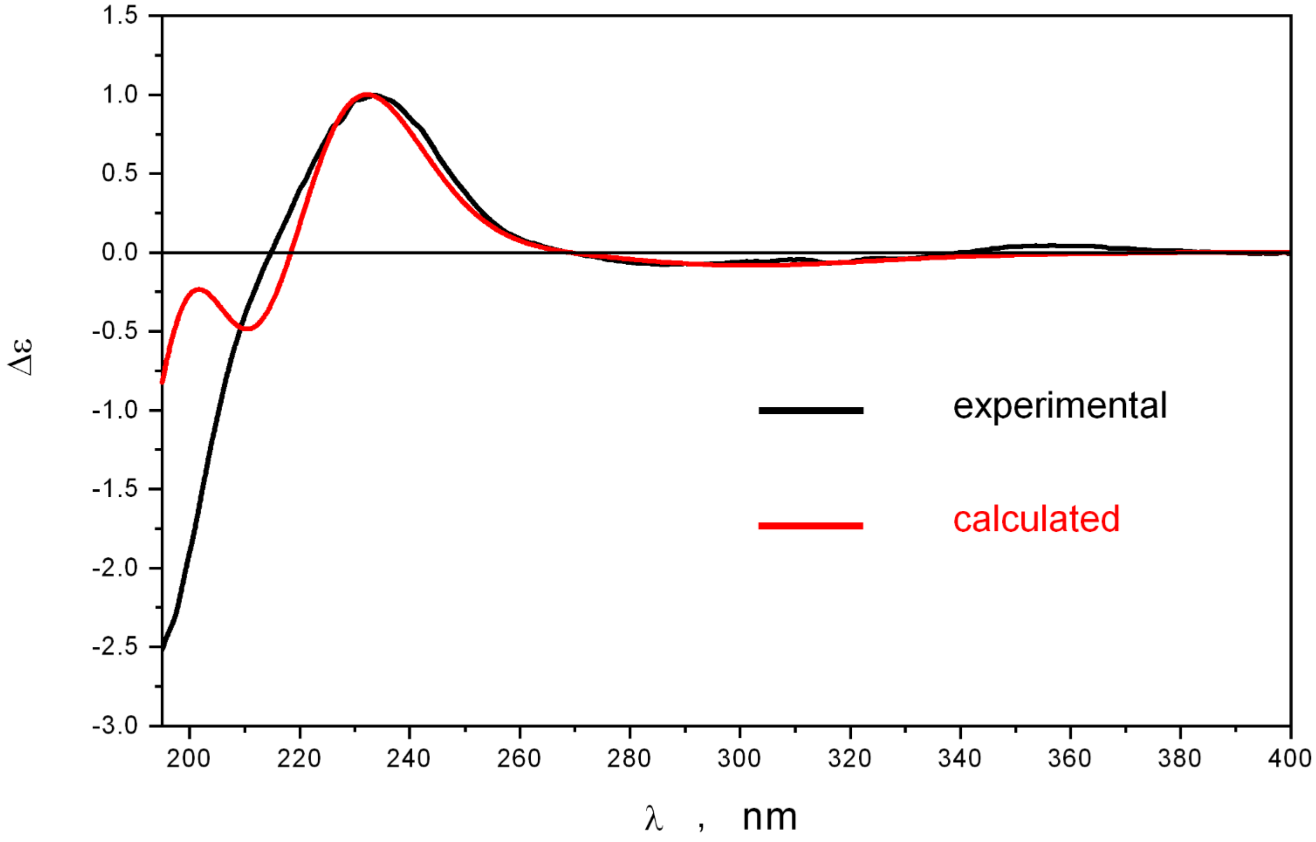

**Figure S36. UV spectrum of 5**

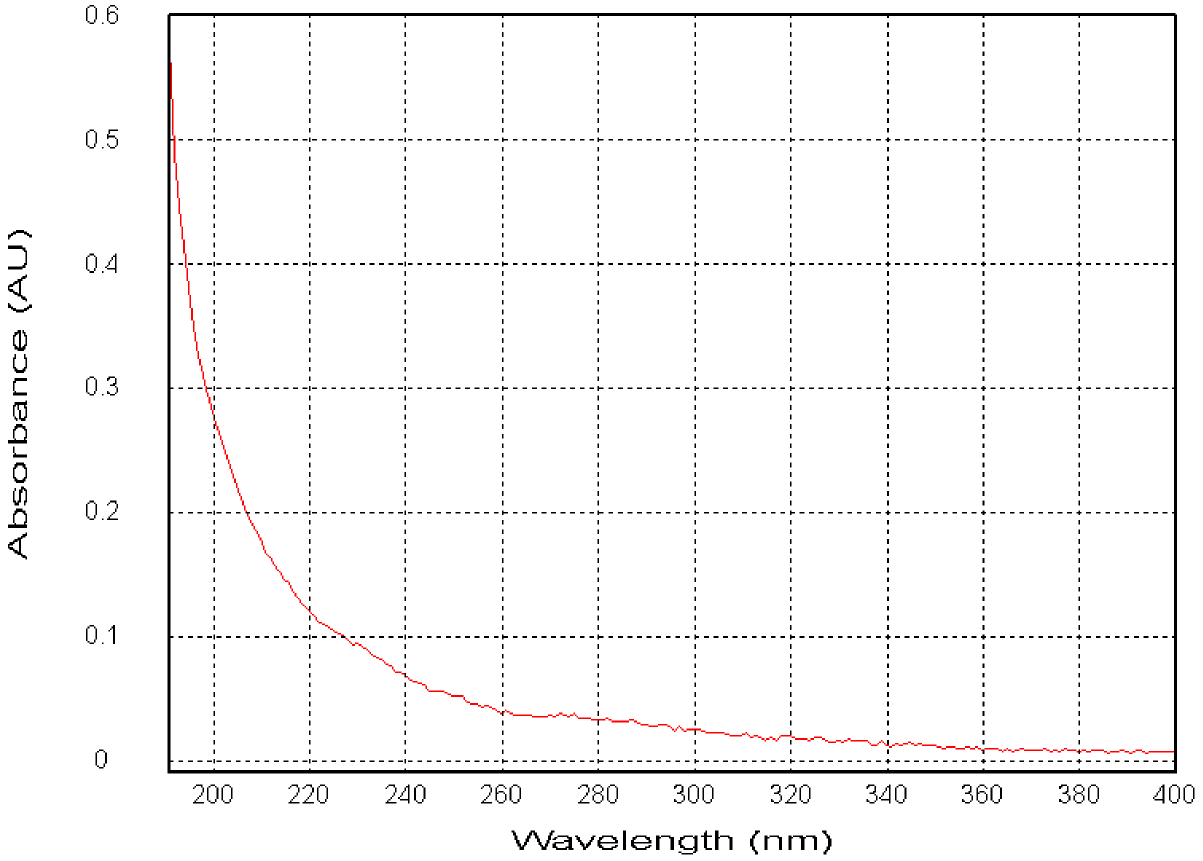


**Figure S37. CD spectrum of 5**

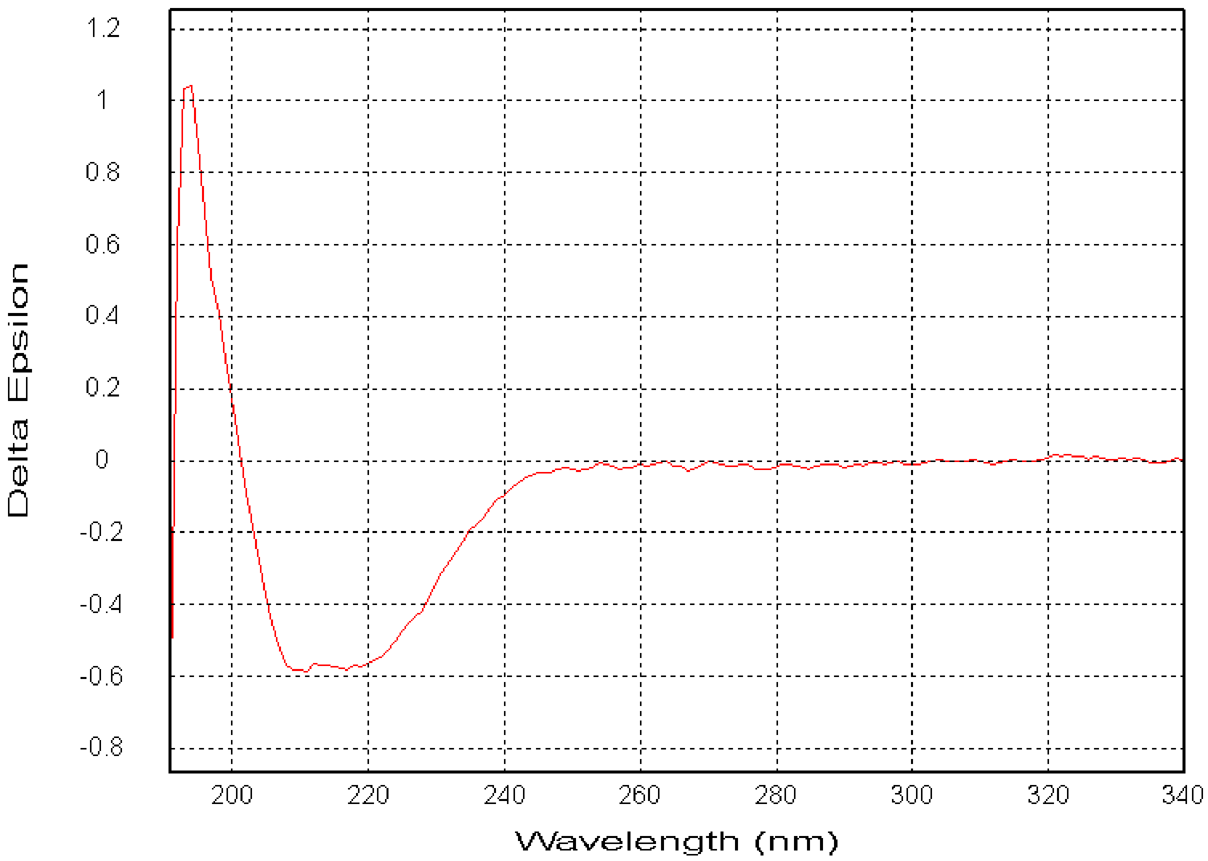

**Figure S38. HR (+)ESI MS spectrum of 1**

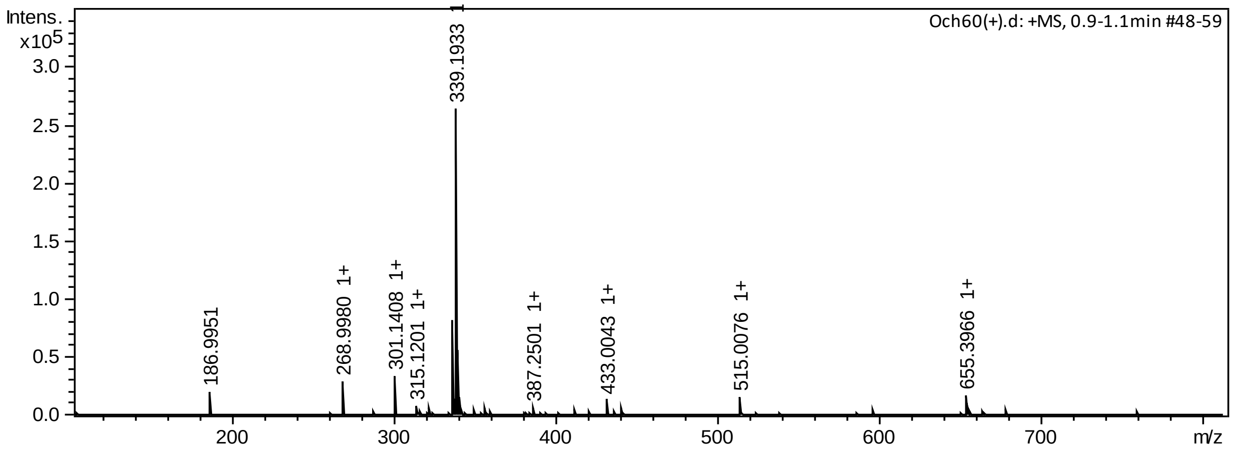


**Figure S39. HR (+)ESI MS spectrum of 2**

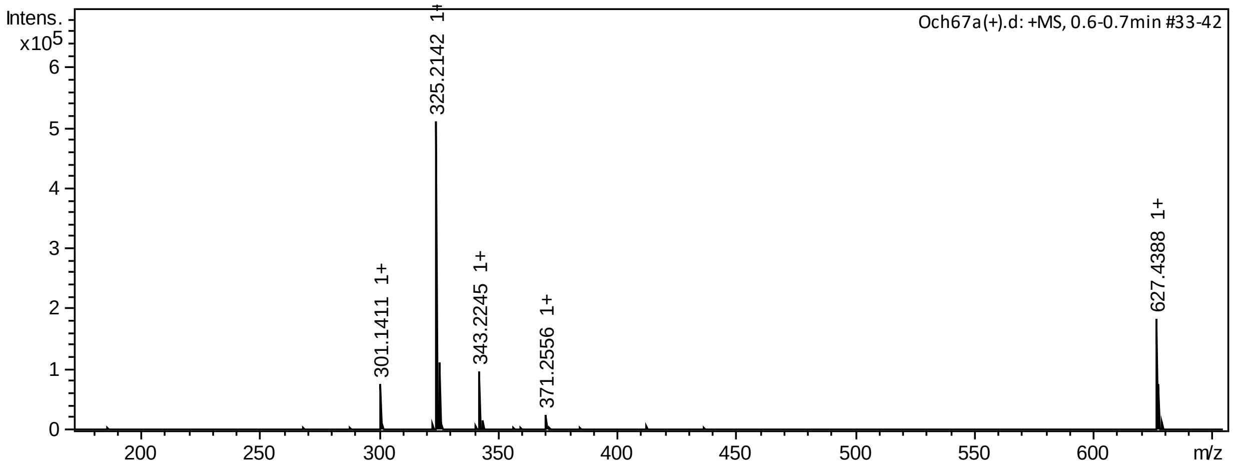


**Figure S40. HR (+)ESI MS spectrum of 3**

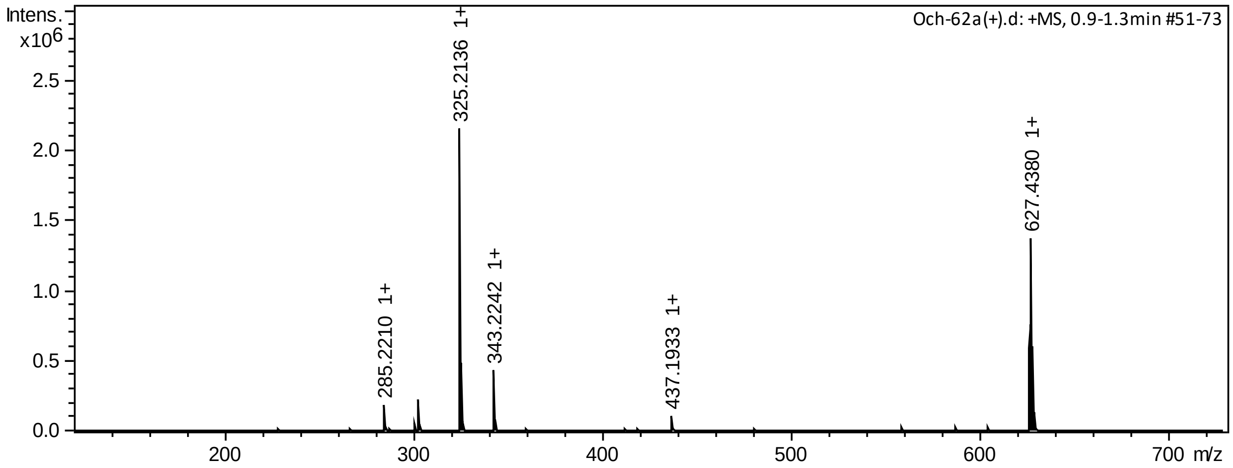

**Figure S41. HR (+)ESI MS spectrum of 4**

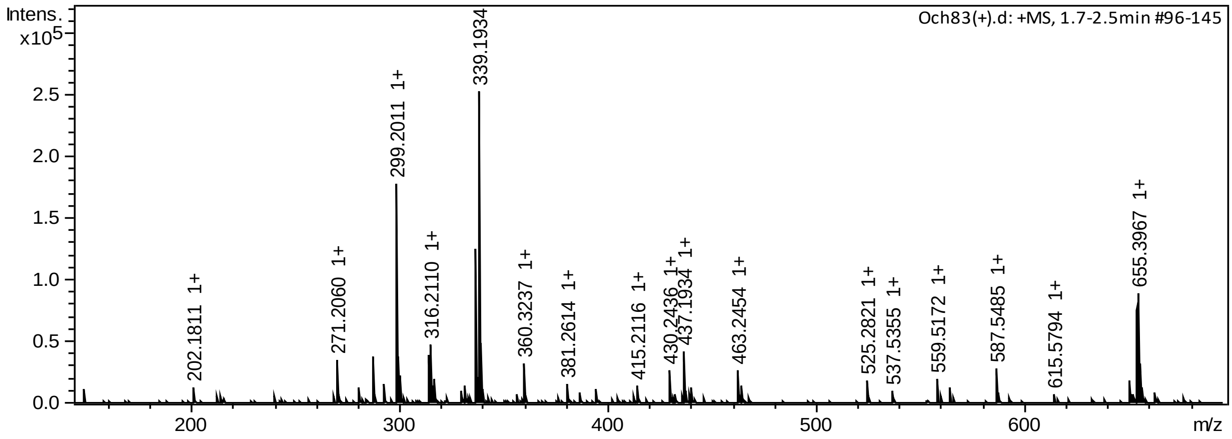


**Figure S42. HR (+)ESI MS spectrum of 5**

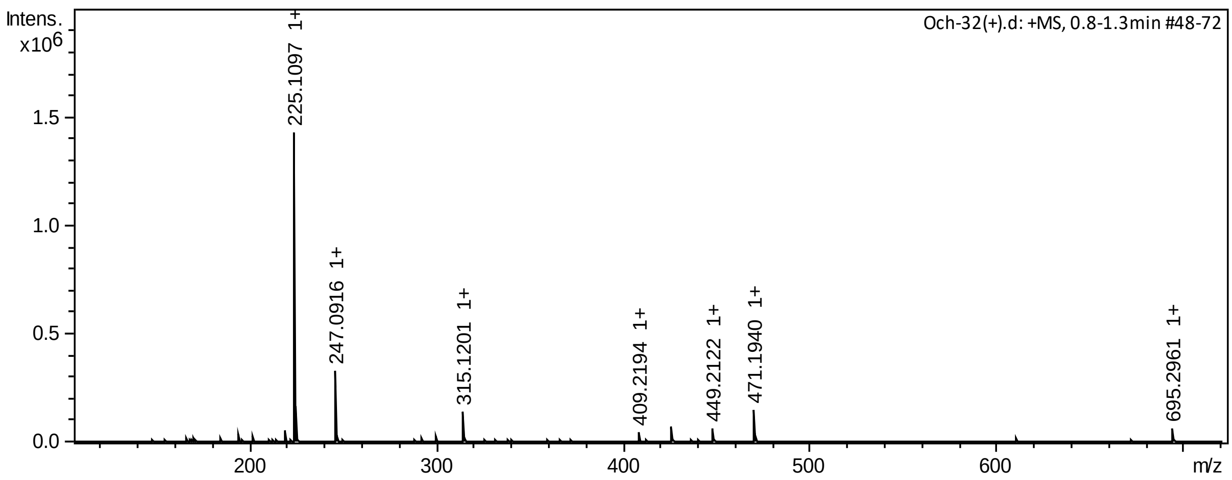


**Figure S43. HR (+)ESI MS spectrum of 6**

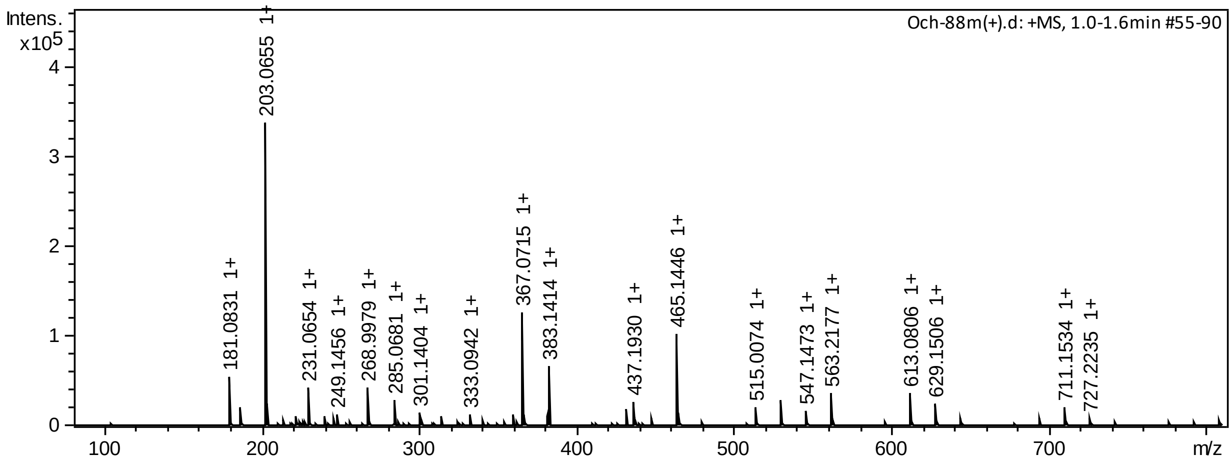

**Figure S44. ¹H NMR spectrum for (*R*)-MTPA esters (500 MHz, CDCl3) of (3b)**

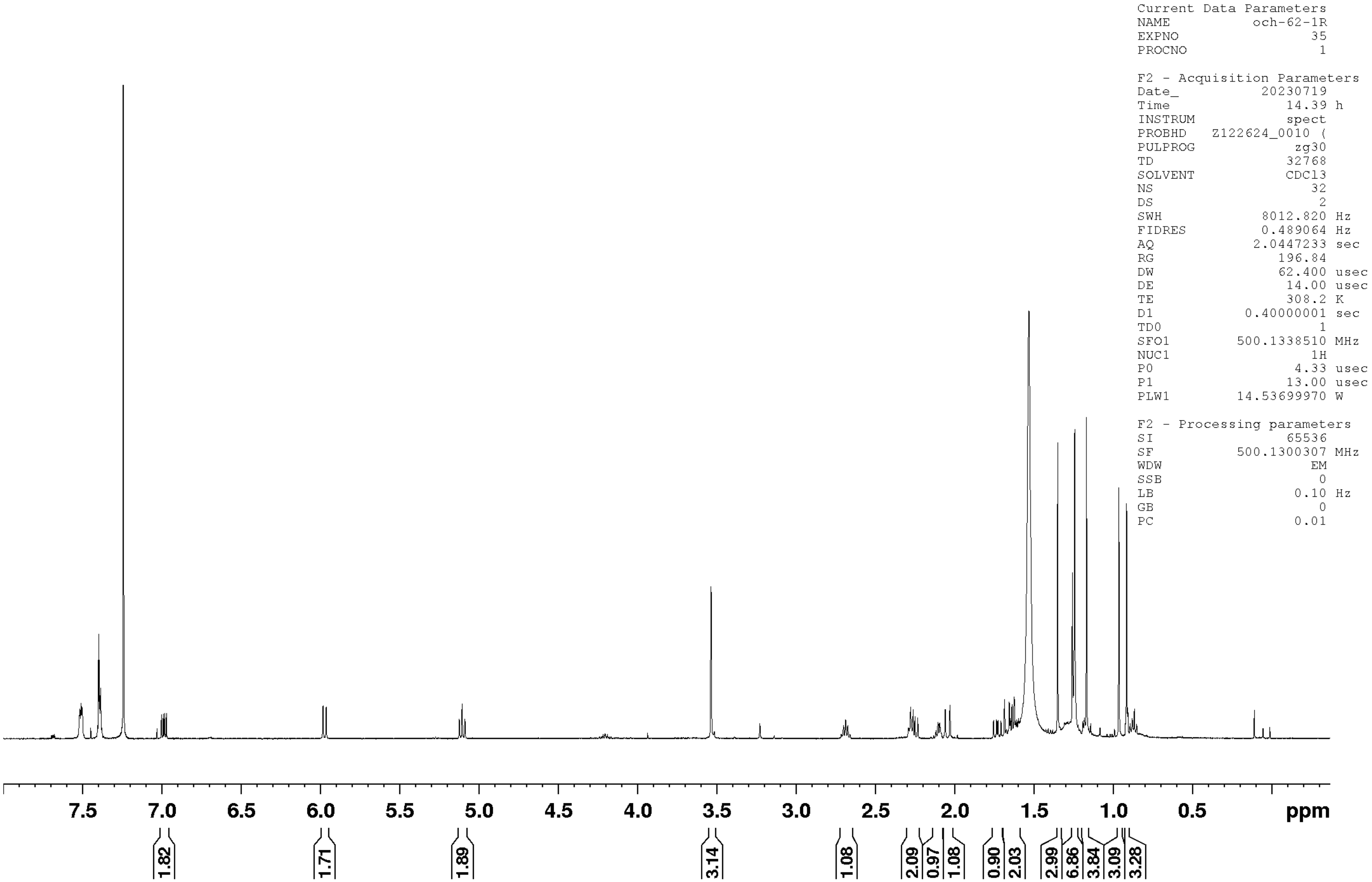

**Figure S45.** $^1$**H-**$^1$**H COSY NMR spectrum for (*R*)-MTPA esters (500 MHz, CDCl3) of (3b)**

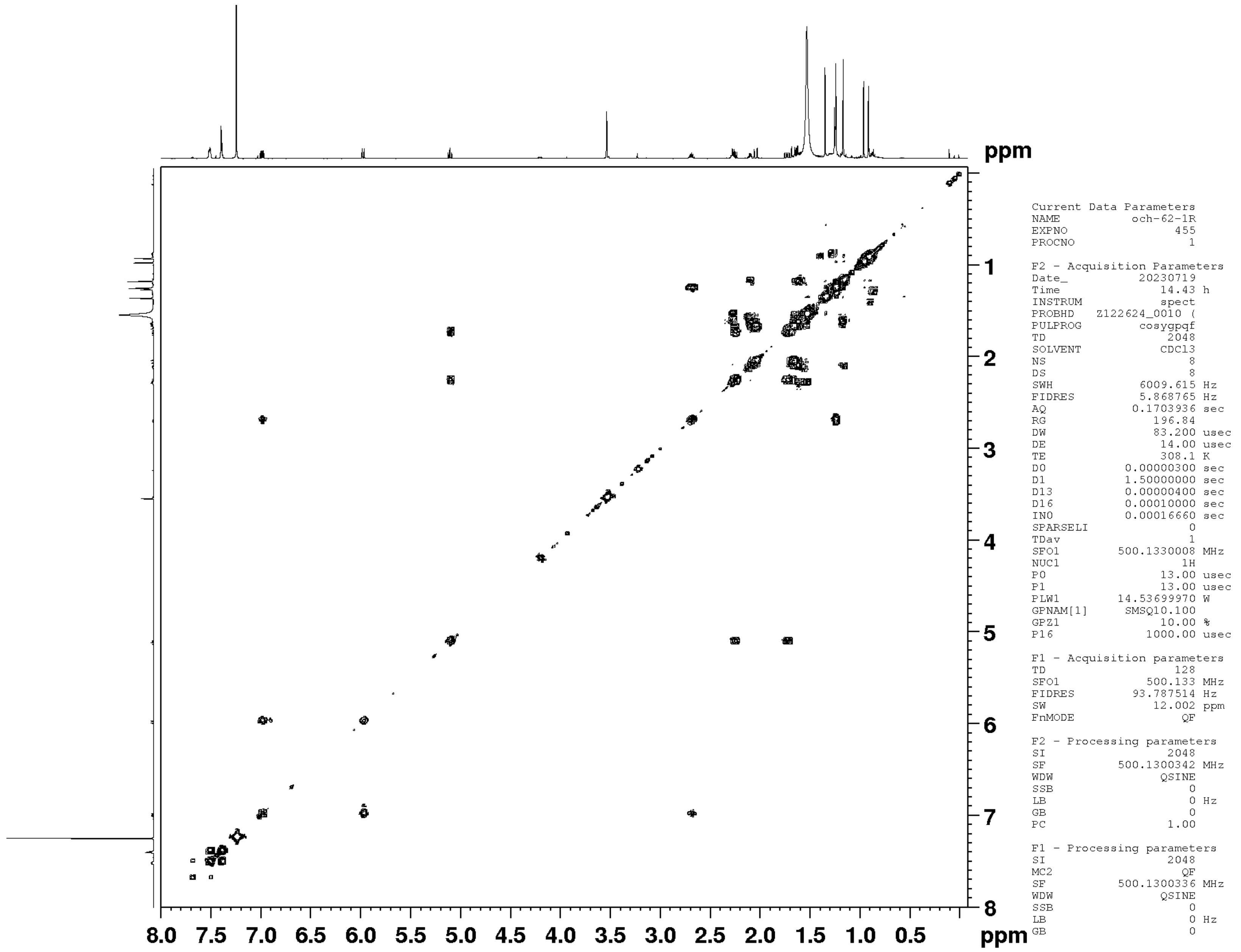

**Figure S46. HR (+)ESI MS spectrum of for (*R*)-MTPA esters of (3b)**

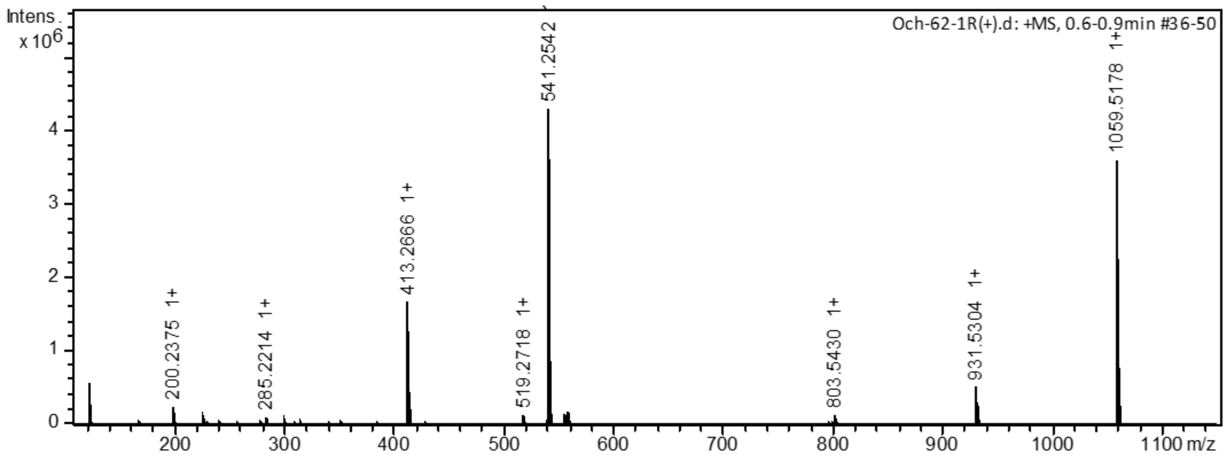

**Figure S47. ¹H NMR spectrum for (*S*)-MTPA esters (500 MHz, CDCl3) of (3a)**

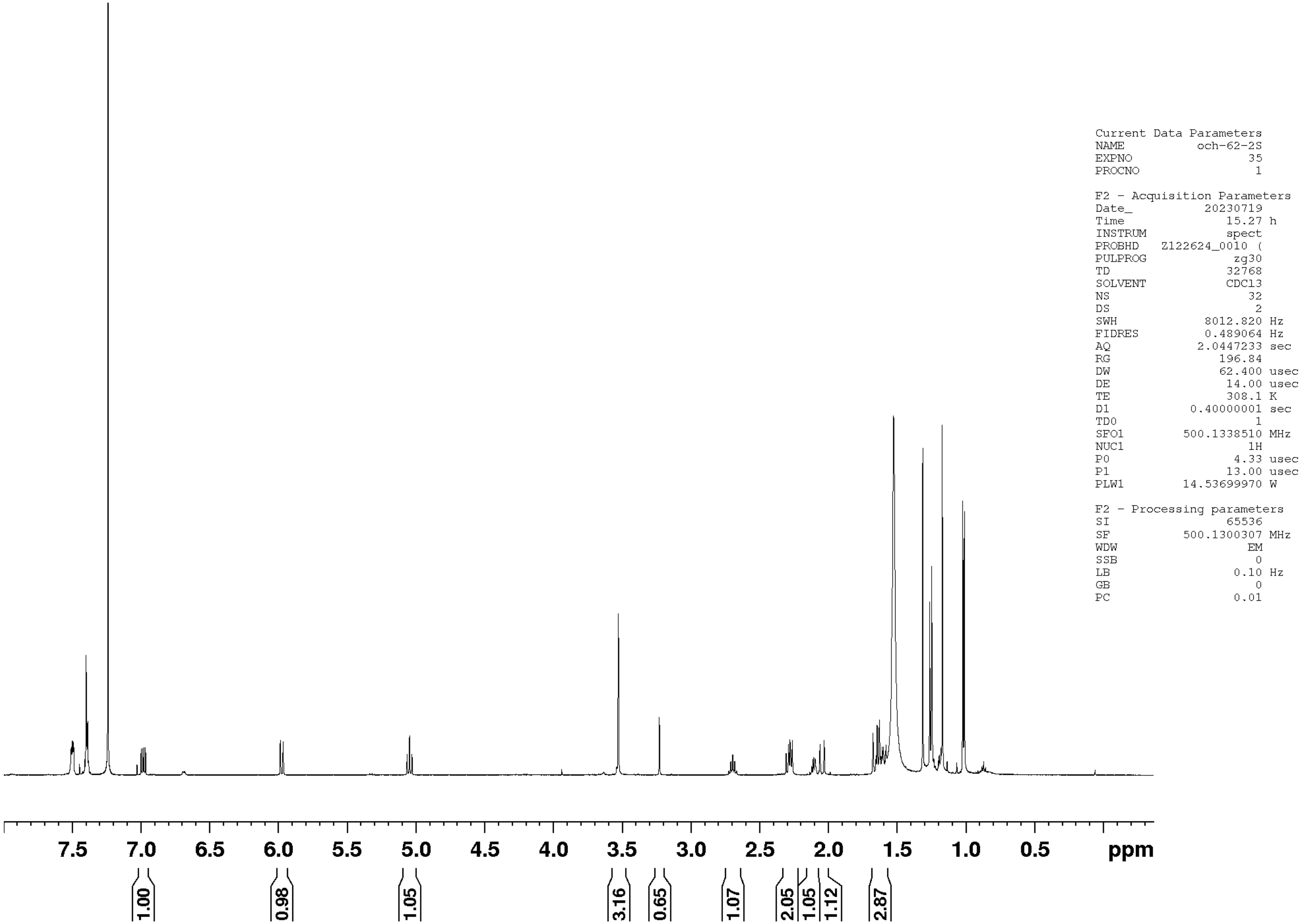

**Figure S48. $^{1}$H-$^{1}$H COSY NMR spectrum for (*S*)-MTPA esters (500 MHz, CDCl3) of (3a)**

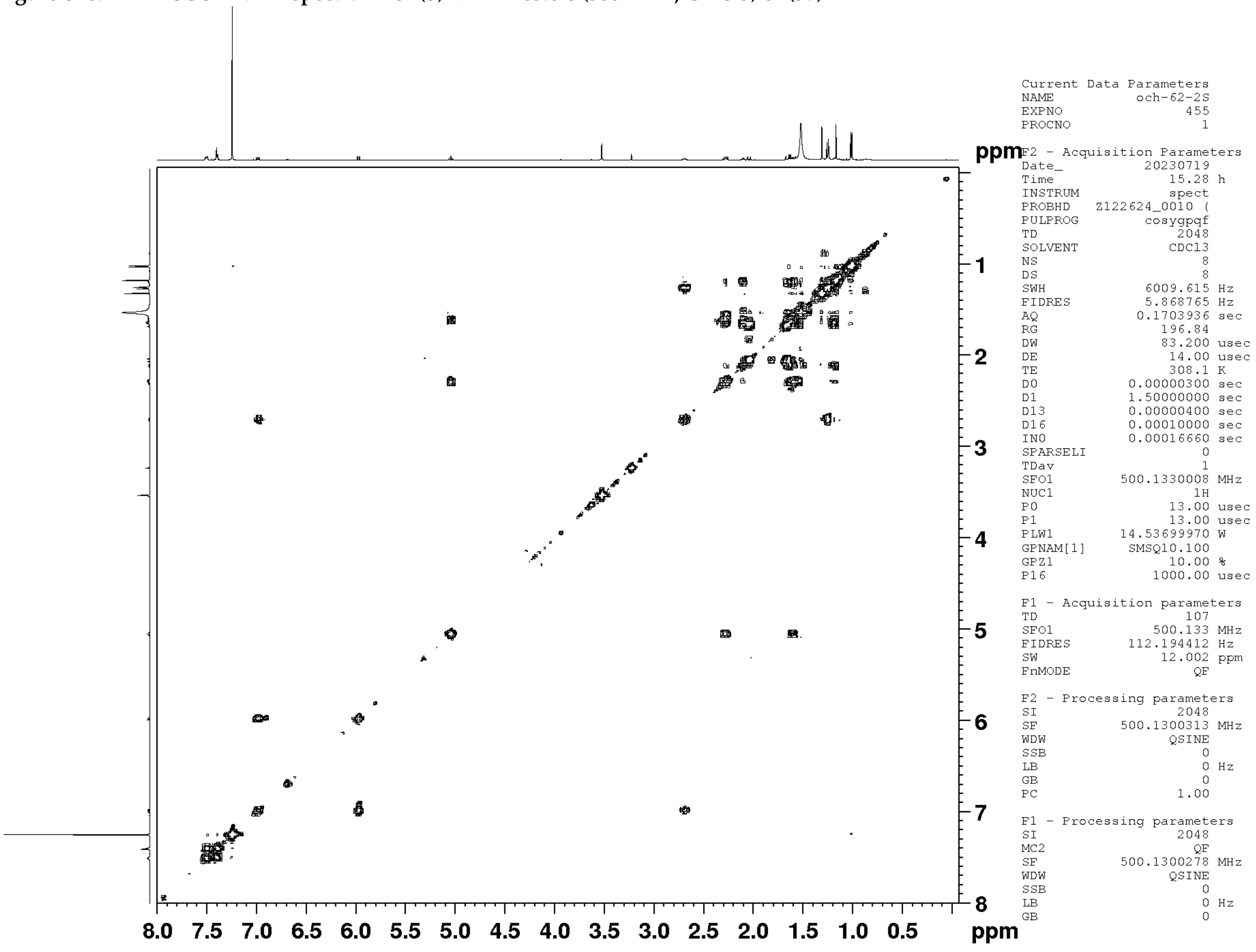

**Figure S49. HR (+)ESI MS spectrum of for (*S*)-MTPA esters of (3a)**

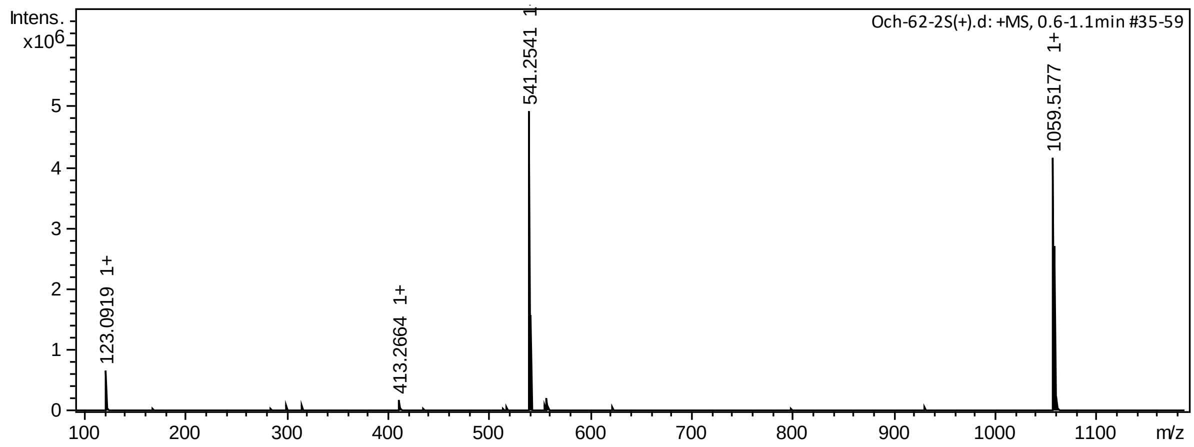


**Figure S50. Comparison of the experimental ECD spectra of compounds 1–4**

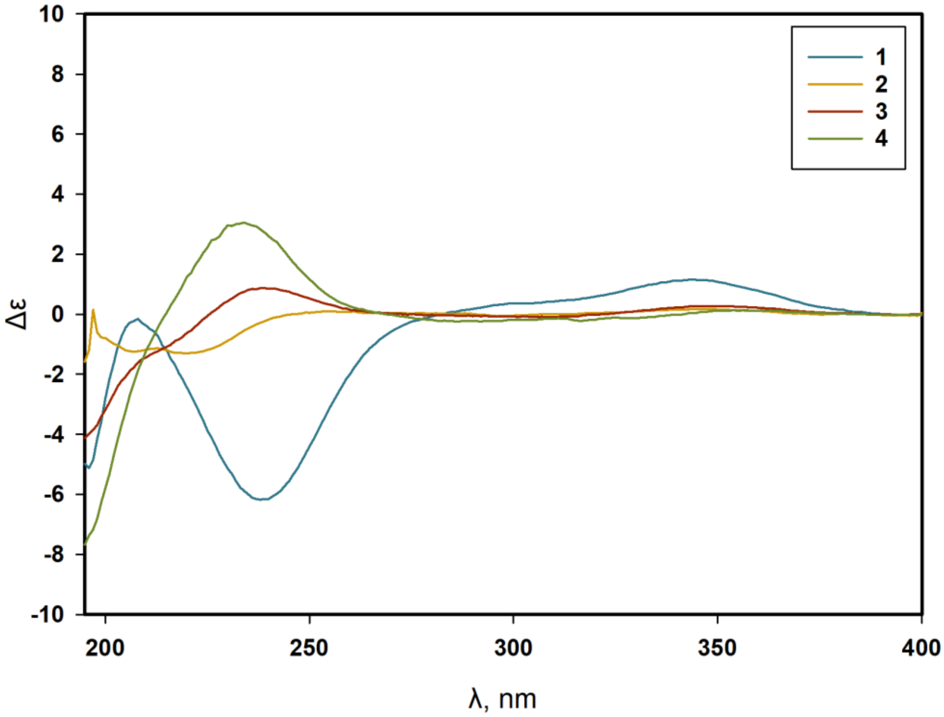

Supplement: Supplementary file 1 [file marinedrugs-21-00584-s001.zip › marinedrugs-2682986-supplementary.pdf]
